# Formation of excess dangling OH bonds during crystallization of amorphous solid water

**DOI:** 10.1038/s41467-026-73221-x

**Published:** 2026-05-14

**Authors:** Linbo Li, Min Lin, Yitian Cao, Haihong Zheng, Jianghui Liu, Yibin Wang, Jiani Hong, HongYing Mao, Haishan Cao, Jian-Qiang Zhong

**Affiliations:** 1 School of Physics, Hangzhou Normal University, Hangzhou, Zhejiang China; 2 Key Laboratory for Thermal Science and Power Engineering of Ministry of Education, Department of Energy and Power Engineering, Tsinghua University, Beijing, China; 3 International Center for Quantum Materials, School of Physics, Peking University, Beijing, China

**Keywords:** Thermodynamics, Surface spectroscopy, Phase transitions and critical phenomena

## Abstract

Dangling OH bonds on ice surfaces are thought to play a central role in surface reactions relevant to planetary, interstellar, and prebiotic environments, yet their direct characterization is hindered by the intrinsic fragility of surface hydrogen-bonding networks, particularly under phase transition conditions. Here, using time-resolved in situ infrared reflection-absorption spectroscopy, we track the evolution of dangling OH bonds during the isothermal crystallization of amorphous solid water into ice I. We observe a transient excess of dangling OH bonds that gradually diminishes as the surface hydrogen-bonding networks reorganize, reflecting competition between nucleation-driven crystallization and surface stabilization. These findings provide direct spectroscopic evidence for a metastable surface state formed during amorphous solid water crystallization and uncover a surface-restructuring pathway that may influence the reactivity of icy surfaces.

## Abstract (web summary)

Direct spectroscopic evidence shows that amorphous ice crystallization produces a transient excess of dangling OH bonds, revealing a nonequilibrium surface state that may enhance chemical reactivity in planetary and interstellar ices.

## Introduction

Water ice, ubiquitous in planetary bodies and the interstellar medium, exhibits structurally complex surface behavior that remains poorly understood^1,2^. At the ice-vacuum interface, incomplete hydrogen-bonding produces dangling OH bonds (dOH), which may serve as catalytically active sites in heterogeneous reactions relevant to atmospheric chemistry, astrochemical synthesis, and prebiotic molecular evolution^3^. In liquid and solid water systems, however, the catalytic roles and mechanistic significance of such dOH species remain subjects of continued debate^4^. Despite extensive studies, the molecular-scale insight into the structure and dynamics of ice and liquid water surfaces still lags behind that of the bulk^5,6^, owing to the structural fragility and extreme sensitivity of surface hydrogen-bonding networks (HBN) to electric fields^7,8^, and to fluctuations in temperature and pressure^9^.

Surface-sensitive probes such as helium atom scattering^10,11^, sum-frequency generation (SFG) spectroscopy^12–15^, and non-contact atomic force microscopy (NC-AFM)^16–18^ have begun to uncover structural motifs at ice surfaces. For example, NC-AFM studies at 4 K revealed a √19 × √19 superstructure on the basal face of hexagonal ice (I*h*) forming near 120 K, stabilized by minimization of electrostatic repulsion among neighboring dOH^16^. These measurements, however, capture only static configurations at cryogenic temperatures and offer limited insight into the dynamic processes relevant to the phase transitions^19^. By contrast, SFG spectroscopy has revealed pronounced temperature dependence of surface HBN on I*h*^20^. Within specific temperature ranges, the I*h* surface exhibits a minimal fraction of dOH, as thermal fluctuations at higher temperatures and recrystallization at lower temperatures disrupt the surface HBN^14^.

Although theoretical studies have refined our understanding of proton ordering and surface energetics in crystalline ice (CI)^21^, the surface structure of amorphous solid water (ASW)—the dominant form of astrophysical ice—remains less accessible^22^. While bulk crystallization of ASW has been extensively examined, the accompanying surface reorganization, particularly the evolution of dOH during phase transitions, is largely unexplored^23,24^.

Classical crystallization theory posits that the transition of ASW into CI is driven by the system’s tendency to minimize its free energy. A significant portion of this thermodynamic driving force arises from the reduction in surface free energy, necessitating molecular rearrangement at the interface^25,26^. Yet the establishment of a stable surface HBN is kinetically impeded by geometric frustration, grain boundary evolution, and cooperative molecular relaxation. As a result, near the crystallization temperature, the ice surface remains highly dynamic, characterized by continual rupture and reformation of the HBN^27^.

Here we employ time-resolved in situ infrared reflection-absorption spectroscopy (IRRAS) to track dOH evolution during isothermal crystallization of vapor-deposited ASW films under ultrahigh vacuum (UHV). By simultaneously probing bulk HBN and surface-specific dOH, IRRAS enables direct correlation between surface and bulk dynamics and uncovers a transient increase in dOH population that emerges during the crystallization process.

## Results

### Spectroscopic signatures of surface and bulk structure in ASW and CI films

Thick ASW and CI films were prepared by vapor deposition onto Ru(0001), Cu(111), and Au(100) substrates under UHV (base pressure ~1 × 10^−10^ torr)^28^. Using multiple substrates allows us to demonstrate that the transient formation of excess dangling OH bonds during ASW crystallization is a universal surface phenomenon, independent of substrate hydrophilicity or crystallographic orientation, as detailed below and in the **Supplementary Information**. Film growth protocols and structural properties followed established procedures^5^. To enhance signal intensity and suppress interference from residual H_2_O, we used deuterated water (D_2_O), whose OD stretching modes (*ν*_OD_, 2100–2800 cm^−1^) fall within the high-sensitivity range of our IRRAS setup^29,30^.

**Fig. 1:**
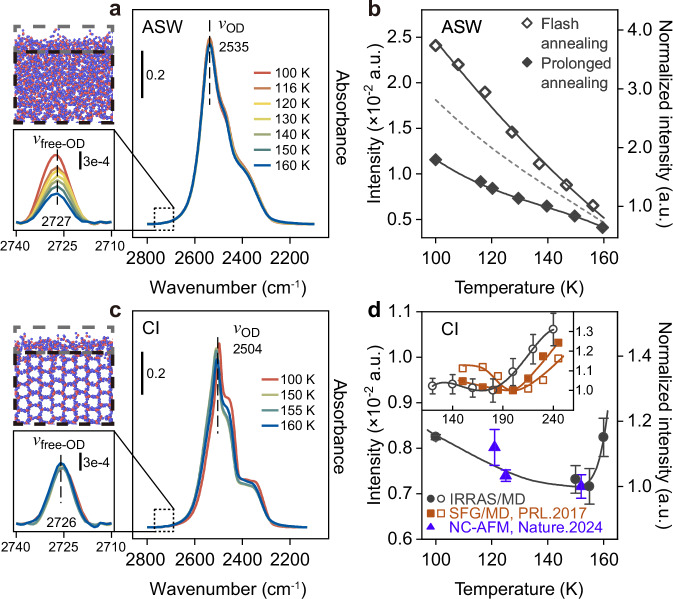
Static characterization of surface-specific free OD modes in ASW and CI films on Ru(0001). **a**, **c** Temperature-dependent IRRAS spectra in the *ν*_OD_ region for ASW and CI, respectively. The lower-left panels enlarge the *v*_free-OD_ region, and the upper-left schematic depicts the molecular structures of ASW and CI obtained from molecular dynamics simulations, with surface and bulk regions marked by gray and black dashed rectangles. Spectra in (**a**, **c**) were recorded at the indicated temperatures. For direct comparison, spectra are normalized (by peak area) to that at 100 K due to the strong thickness dependence of the bulk *ν*_OD_ signal, whereas the surface-specific *v*_free-OD_ signal is presented without normalization. **b**, **d** Corresponding *v*_free-OD_ intensities as a function of temperature for ASW (solid diamonds) and CI (solid circles), respectively. In **b**, **d**, solid lines represent polynomial fits to guide the eye, while the central dashed line in **b** indicates the average of the two polynomial fits (see **Supplementary Text**). In **d**, previously reported CI data (blue triangles) are included for comparison. The inset shows MD simulation results from this study (hollow circles) alongside earlier experimental and computational data (orange squares). Error bars in **d** represent statistical uncertainties derived from two independent measurements at 100 K and twenty independent measurements at 150 K, 155 K, and 160 K, whereas those in the inset are obtained from 2001-frame trajectories over 4 ns.

ASW and CI films exhibit distinct *ν*_OD_ absorbance features, reflecting fundamental differences in bulk HBN (Fig. 1a, c, Supplementary Fig. 1)^31–33^. Within these networks, water molecules primarily adopt configurations such as DDAA, DDA, DAA, and DA, depending on their roles as hydrogen-bond donor (D) and acceptor (A). Considerable efforts have been devoted to identifying the subcomponents contributing to the broad *ν*_OD_ stretching band observed in IRRAS spectra^34,35^; however, full deconvolution remains challenging, as proton disorder and variations in O–O distances or O–D bond lengths lead to substantial spectral broadening^34,35^. Nevertheless, upon crystallization, the dominant *ν*_OD_ band redshifts from ~2535 cm^−1^ in ASW to ~2504 cm^−1^ in CI, consistent with stronger, more ordered hydrogen bonding^36^. These most intense peaks are therefore used as characteristic signatures of ASW and CI, respectively^36^.

Whereas the main *ν*_OD_ band reflects bulk order, surface-specific information derives from the free OD mode (*v*_free-OD_, 2710–2740 cm^−1^), which corresponds to the dangling OD bonds (dOD) at the ice-vacuum interface^37^. As shown in the enlarged views in Fig. 1a, c, the temperature dependence of the *ν*_free-OD_ intensity is pronounced for ASW but much weaker for CI. In ASW films (Fig. 1b), spectra collected after prolonged annealing (~10 min, solid diamonds) differ noticeably from those obtained after flash annealing followed by rapid cooling (<1 min, hollow diamonds), with both protocols controlled to avoid bulk crystallization. The region between these two datasets defines the range of *v*_free-OD_ intensities accessible under different thermal histories. Overall, *v*_free-OD_ decreases with increasing temperature up to ~160 K, consistent with progressive reorganization of the surface HBN. In contrast, CI films (Fig. 1d) exhibit only a weak temperature dependence: the *v*_free-OD_ intensity displays a shallow minimum near 155 K, a behavior also reproduced by our molecular dynamics (MD) simulations (inset, Fig. 1d). A similar trend has been reported in SFG studies of ice I*h* at 200 K, likely due to differences in experimental conditions (UHV *versus* atmospheric pressure)^14^.

### Crystallization kinetics of ASW films

**Fig. 2:**
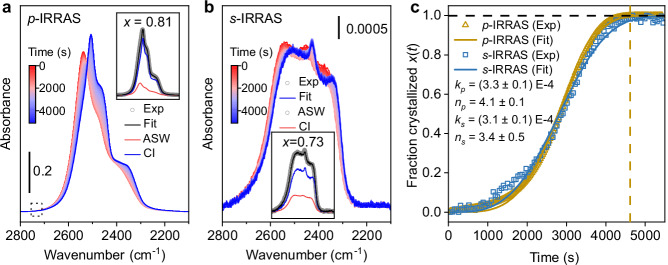
Time-resolved IRRAS analysis of ASW crystallization kinetics. **a** *p*-polarized and **b** *s*-polarized IRRAS spectra in the *v*_OD_ region during isothermal crystallization of ~110 nm-thick ASW films on Ru(0001) at 155 K. The initial spectrum (red) corresponds to the fully amorphous state ($x=0$), and the final spectrum (blue) to the crystalline state ($x=1$). Insets illustrate the spectral deconvolution, where each intermediate spectrum is modeled as a linear combination of ASW and CI components to extract the crystallinity fraction $x(t)$. **c** Time-dependent $x(t)$ derived from **a** and **b**, fitted with the Avrami equation, where $k$ and $n$ denote the effective crystallization rate constant and the Avrami exponent, respectively. The dashed vertical line marks the completion of crystallization.

Figure 2 shows the isothermal crystallization kinetics of thick ASW films on Ru(0001). Although the influence of film thickness^38,39^ and the mechanisms of surface *versus* bulk crystallization^40–43^ remain debated, we focus on structural evolution under controlled thermal conditions.

ASW films were deposited at 100 K and annealed rapidly to 155 K and held isothermally for time-resolved *p*-polarized IRRAS measurements (Fig. 2a). As crystallization progressed, the *ν*_OD_ bands gradually red-shifted, and all spectra intersected at an isosbestic point. This feature indicates that the overall absorbance can be approximated as a linear combination of two phases, ASW and CI, under the assumption of a two-state (ASW/CI) mixture model (Supplementary Fig. 2). Such analysis has been widely and successfully applied to characterize structural transitions in various amorphous and crystalline molecular films^24,37,40,44^. Using this approach, the crystallinity was extracted by decomposing each spectrum into ASW and CI components. For example, at $t=3450$ s, the best-fit yielded 19% ASW and 81% CI, corresponding to a crystallinity fraction $x(t)=0.81$ (inset, Fig. 2a). The kinetics were described by the Avrami equation:

1 $$x(t)=1-\exp \left[-({kt})^{n}\right]$$

where $k$ is the effective crystallization rate constant encompassing both nucleation and growth, and $n$ the Avrami exponent reflecting the crystallization dimensionality^45^ (Supplementary Fig. 3). Complementary analysis of absorbance in the 2460–2470 cm^−1^ region confirmed these results (Supplementary Fig. 4)^36,46–48^.

To probe the anisotropy in crystallization, we performed complementary *s*-polarized IRRAS measurements (Fig. 2b). To our knowledge, this is the first polarization-resolved IRRAS study of ASW crystallization^49,50^. At ~110 nm thickness, the ice films behave dielectrically like oxides, suppressing the surface-selection rule and enabling detection of in-plane dipoles^51^. Thus, *p*-polarized IRRAS probes out-of-plane modes, while *s*-polarized IRRAS is sensitive to in-plane modes. Fitting $x\left(t\right)$ yielded Avrami exponents of $n=4.1\pm 0.1$ (*p*-IRRAS) and $n=3.4\pm 0.5$ (*s*-IRRAS), indicating anisotropic three-dimensional nucleation and growth (Fig. 2c, Supplementary Fig. 5)^5^. Crystallization proceeded slightly faster along the surface normal than laterally. During early stages of crystallization, the experimental data deviate from the Avrami prediction, a phenomenon widely reported across multiple characterization methods^36,43,52^. The magnitude and even the direction of these deviations depend sensitively on crystallization temperature and on ASW film properties such as thickness and porosity^36,38,40,43^. These observations suggest the presence of a pre-ordering phase preceding bulk crystallization, a regime that merits further investigation^5,53^.

### Surface structure evolution during crystallization

**Fig. 3:**
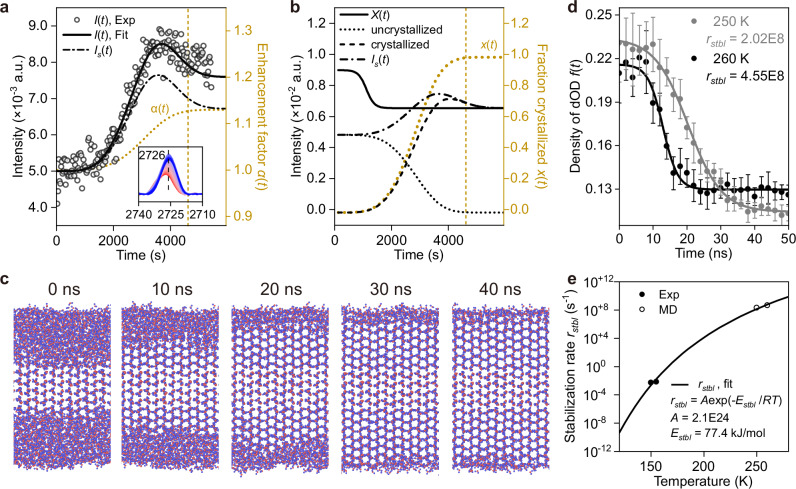
Surface dOD evolution during isothermal crystallization. **a** Time-dependent integrated intensity of the *v*_free-OD_ mode during isothermal crystallization at 155 K, fitted using $I\left(t\right)={I}_{{{\rm{s}}}}\left(t\right) \cdot {{\rm{\alpha }}}\left(t\right)$ as described in the main text. The inset shows representative *v*_free-OD_ spectra corresponding to the region highlighted by the dashed rectangle in Fig. 2a. **b** Surface dOD contribution ${I}_{{{\rm{s}}}}\left(t\right)$ extracted by accounting for surface stabilization, modeled using a logistic decay function $X(t)$. **c** Representative snapshots from MD simulations of ASW crystallization at 260 K, illustrating the evolution of the surface structure. **d** Temporal evolution of the surface dOD density from statistical analysis of MD simulations at 250 K and 260 K. Error bars are obtained from 11-frame trajectories over 200 ps centered around the indicated crystallization time. **e** Experimentally determined surface stabilization rates at 150 K and 155 K, alongside simulation-derived rates at 250 K and 260 K, fitted with a temperature-dependent stabilization function ${r}_{{stbl}}(T)$, showing consistent kinetics and a quantitative link between experiment and molecular-level simulations.

Surface dynamics during crystallization were monitored by the time-dependent IRRAS intensity $I\left(t\right)$ of the *v*_free-OD_ mode at ~2726 cm^−1^. As shown in Fig. 3a, bulk crystallization at 155 K was completed within ~4600 s. During this period, $I\left(t\right)$ exhibited a distinct transient peak: an initial gradual rise was followed by a sharp increase to a maximum, then decayed to a steady-state plateau. Notably, the peak occurred before bulk crystallization was complete and was also observed during the crystallization of ASW films on Cu(111) and Au(100) (Supplementary Figs. 6–9), albeit with different temperatures and timescales, reflecting variations in the initial amorphous structure.

The evolution of $I\left(t\right)$ is governed by two principal contributions: a surface-specific component ${I}_{{{\rm{s}}}}\left(t\right)$, proportional to the dOD population, and a bulk enhancement factor ${{\rm{\alpha }}}\left(t\right)$, accounting for the increased IR absorption due to structural ordering during crystallization:

2 $$I\left(t\right)={I}_{{{\rm{s}}}}\left(t\right)\cdot {{\rm{\alpha }}}\left(t\right)$$

with ${{\rm{\alpha }}}\left(t\right)=1+0.13\cdot x\left(t\right)$ (see Supplementary Text and Supplementary Fig. 10).

While $x\left(t\right)$ follows the expected sigmoidal profile governed by Avrami kinetics, the transient overshoot in $I\left(t\right)$ suggests that the dOD population does not scale linearly with crystallinity, and additional surface-specific processes must be considered.

We introduce a surface stabilization process, concurrent with nucleation and growth, that reduces the dOD population as crystallization proceeds (Fig. 3b). This non-equilibrium process is described by a logistic decay function:

3 $$\frac{{dX}\left(t\right)}{{dt}}={-r}_{{stbl}}X\left(t\right)\left(1-\frac{X\left(t\right)}{K}\right)$$

where $X(t)$ is the dOD contribution from a nucleus formed at time $t$, ${r}_{{stbl}}=A\exp (-{E}_{{stbl}}/{RT})$ is the stabilization rate constant with ${E}_{{stbl}}$ as the activation energy, and $K$ is the initial (maximum) intensity (Supplementary Table 1). This form captures the nonlinear relaxation of surface dOD driven by a competition between electrostatic repulsion among adjacent OD groups and thermal fluctuations^14,54,55^. MD simulations support this mechanism, showing a pronounced decrease in dOD density with increasing crystallite size (Fig. 3c, d, Supplementary Figs. 11, 12). The stabilization process is strongly temperature-dependent and governs the surface dOD population (Fig. 3e).

Assuming spatially random nucleation, the total surface dOD signal evolves as the cumulative contribution from all nuclei:

4 $${I}_{{{\rm{s}}}}\left(t\right)\propto \int _{0}^{t}\frac{{dx}}{d\tau }\cdot X\left(t-\tau \right)d\tau+{{\rm{\beta }}}\left[1-x\left(t\right)\right]$$

where $\frac{{dx}}{d\tau }$ is the crystallization rate at time $\tau$, and $X\left(t-\tau \right)$ describes the time-dependent decay of dOD from a nucleus formed at $\tau$. The first term integrates the dOD contributions from all crystallites, each modulated by its stabilization trajectory. The second term, ${{\rm{\beta }}}(1-x\left(t\right))$, accounts for residual dOD in the non-crystallized regions, with ${{\rm{\beta }}}=0.005$ as the experimentally determined baseline dOD intensity in pure ASW.

The initial rise in ${I}_{{{\rm{s}}}}\left(t\right)$ reflects the formation of numerous small crystalline nuclei (or domains) that collectively increase the dOD population. The subsequent decline results from the progressive dOD elimination via surface stabilization, occurring alongside the conversion of amorphous regions into crystalline domains (Fig. 3d). These findings are corroborated by complementary optical and Raman measurements (Supplementary Fig. 13, Supplementary Table 2). Overall, this integrated model reproduces the transient *v*_free-OD_ profile, providing a unified framework for understanding coupled surface and bulk structural dynamics within the Avrami crystallization paradigm (Fig. 3a).

### Formation of excess dOD

**Fig. 4:**
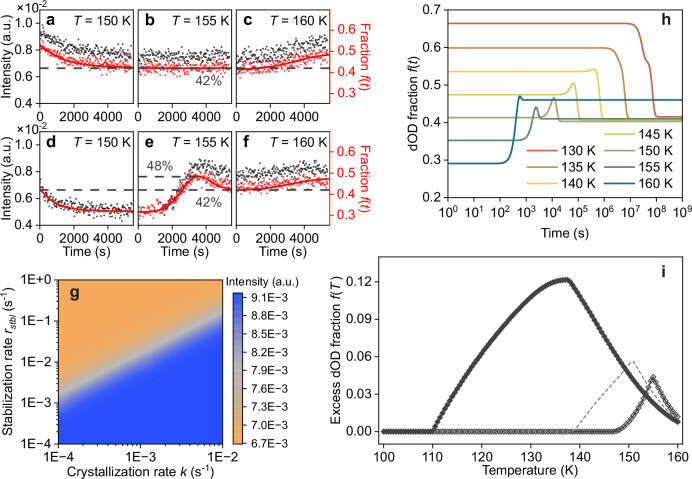
Quantifying excess dOD formation during isothermal crystallization. **a–c** Time-resolved evolution of the *v*_free-OD_ intensity and the dOD fraction in CI films subjected to isothermal annealing at 150 K, 155 K, and 160 K, respectively. **d–f** Corresponding evolution of the *v*_free-OD_ intensity and the dOD fraction in ASW films under identical thermal conditions. CI films were prepared at 145 K and ASW films at 100 K, each with a nominal thickness of ~110 nm. Dashed gray lines mark the minimum dOD fraction previously reported for CI surfaces, serving as reference baselines. **g** Simulated effect of the relative rates of surface stabilization (${r}_{{stbl}}$) and crystallization ($k$) on excess dOD formation during ASW crystallization at 155 K. **h** Simulated temperature dependence of excess dOD formation, using the initial and final dOD fractions taken from the central dashed line in Fig. 1b and the solid black line in Fig. 1d, respectively, and the rate constants ${r}_{{stbl}}$ and $k$ taken from Fig. 3e, Supplementary Fig. 3a. **i** Extracted excess dOD fraction as a function of temperature from the simulations in (**h**), shown as the central dashed curve. The solid curves, marked by solid and hollow diamonds, indicate the range of excess dOD values arising from variations in the initial surface configurations of ASW, as shown in Fig. 1b.

The surface stabilization function captures the time-dependent evolution of dOD following temperature perturbations, quantitatively describing structural reorganization toward new equilibrium states. It applies broadly to ASW and CI films, as well as to isothermal ASW-to-CI crystallization (Fig. 4a–f).

In a representative case (Fig. 4a), a CI film stabilized at 145 K was rapidly annealed to 150 K, resulting in a logistic decay in *v*_free-OD_ intensity from 9.3 × 10^−3^ to 7.5 × 10^−3^, reflecting progressive surface reconstruction and extended surface HBN formation. Further heating to 160 K reversed this trend, with *v*_free-OD_ intensity recovering to 8.5 × 10^−3^ (Fig. 4c), consistent with partial disruption of stabilized HBN and re-emergence of dOD at elevated temperatures. Notably, a minimum intensity near 155 K, together with little change between 150 K and 155 K (Fig. 4b), indicates a transition point in the surface stabilization dynamics.

On the ideal ice I*h* (0001) surface, the topmost bilayer consists of 50% DAA and 50% DDA molecules, with dOD contributed solely by DAA. This configuration is thermodynamically unstable, especially above ~120 K, driving surface reconstruction to minimize surface free energy^16,20^. While directly linking *v*_free-OD_ intensity ${I}_{{{\rm{s}}}}\left(t\right)$ to the absolute dOD fraction $f\left(t\right)$ remains challenging, the temperature dependence of ${I}_{{{\rm{s}}}}\left(t\right)$ aligns with earlier reports (Fig. 1d). Based on NC-AFM measurements under comparable conditions, we assign the minimum *v*_free-OD_ intensity (~7.5 × 10^−3^) to ~42% dOD fraction^16^. Similar minimum fraction has been reported in SFG studies on ice I*h*^14^.

Assuming a linear relation between $f\left(t\right)$ and ${I}_{{{\rm{s}}}}\left(t\right)$, the temporal evolution of dOD fraction can be estimated (red lines in Fig. 4a–c). These values are semi-quantitative, as the calibration relies on indirect referencing to NC-AFM and the linear approximation neglects possible effects of dipole orientation and intermolecular coupling on the IRRAS intensity. Nevertheless, this approach remains reasonable given the dynamic disorder and vibrational activity of surface water molecules. Similar to SFG, IRRAS probes time-averaged dOD populations. Peak position and full width at half maximum (FWHM) analyses indicate little change in dOD orientation (Supplementary Figs. 14, 15), supported by MD simulations (Supplementary Fig. 16). However, spectral broadening near 160 K suggests increased surface disorder^14^.

A similar analysis was applied to ASW film and its isothermal crystallization, but required correction for bulk enhancement, yielding ${I}_{{{\rm{s}}}}\left(t\right)=I\left(t\right)/{{\rm{\alpha }}}\left(t\right)$ (Fig. 4d–f). In Fig. 4d, annealing an ASW film at 150 K shows a rapid decrease in *v*_free-OD_ intensity, indicating that the initially disordered, dOD-rich surface reorganizes into extensive surface HBN in the absence of bulk crystallization (Supplementary Fig. 17). Upon heating to 155 K (Fig. 4e), crystallization accelerated, and *v*_free-OD_ stabilized near 7.5 × 10^−3^, matching the equilibrium value for CI. A transient maximum appears in Fig. 4e but not in Fig. 4b, despite identical temperatures, implying ~6% excess dOD formed during isothermal crystallization.

This excess arises from the competition between two thermally activated processes: the crystallization rate $k$, which promotes dOD formation through nucleation-driven surface restructuring, and the stabilization rate ${r}_{{stbl}}$, which suppresses dOD through the re-establishment of energetically favorable HBN. By neglecting the film-specific variables such as thickness and microscopic ASW morphology, excess dOD emerges only when $\frac{{r}_{{stbl}}}{k} < \sim 180$ (Fig. 4g, Supplementary Fig. 18), with similar behavior observed for the crystallization at 150 K and 160 K (Supplementary Fig. 19). This can be rationalized as follows: when ${r}_{{stbl}}\gg k$, stabilization rapidly eliminates dOD generated by nucleation, producing a quasi-static surface and preventing excess dOD.

To further examine the temperature dependence of this kinetic competition, we simulated the crystallization of 100 nm-thick ASW films subjected to abrupt heating from 100 K to various higher temperatures. The initial and final dOD intensities were referenced from the static measurements shown in Fig. 1b, d. Notably, the extent of transient excess dOD formation depends strongly on both the isothermal crystallization temperature and initial surface configuration of the ASW films (Fig. 4h, i, **Supplementary text)**.

## Discussion

Surface stabilization is thermodynamically driven by the minimization of surface free energy through molecular reorganization. In the absence of bulk crystallization (e.g., in pure ASW or fully CI), the process is governed by the free energy difference ($\Delta G$) between the initial and final surface configurations at a given temperature. Consequently, the stabilization rate ${r}_{{stbl}}$ depends on both $\Delta G$ and temperature. During the ASW-to-CI transition, however, surface restructuring occurs concurrently with bulk crystallization. The surface dynamics are therefore governed by two coupled processes: the crystallization rate $k$ and the stabilization rate ${r}_{{stbl}}$. For isothermal crystallization at 155 K following a rapid temperature jump from 150 K, the initial value of $\Delta G$ is estimated to be 973 J/mol from the measured desorption rates of ASW and CI films (see **Supplementary Text**, Supplementary Figs. 20, 21)^36^. As crystallization proceeds and the crystalline fraction increases, $\Delta G$ progressively decreases, reducing the thermodynamic driving force for further surface restructuring. Under UHV conditions, thermal desorption is unavoidable and becomes increasingly significant at elevated temperatures, potentially leading to a regime in which desorption dominates the surface dynamics. Consequently, the transient surface structure during the ASW-to-CI transformation is governed by the interplay among crystallization, surface stabilization, and thermal desorption, a regime requiring further exploration.

In conclusion, we present direct spectroscopic evidence for the transient formation of excess dOD during ASW crystallization. These non-equilibrium features alter the physicochemical landscape of ice surfaces by increasing the population of highly undercoordinated, and therefore highly reactive, surface sites^45,56,57^. Previous studies have also shown that introducing acids (HCl or HNO_3_) or salts (NaCl) into ASW can modify the crystallization rate $k$, and may therefore influence both the magnitude and duration of excess dOD formed during the ASW-to-CI transition^46,58^. These findings have important implications for astrochemical environments. In interstellar and planetary ices, where ASW is the dominant phase but episodic heating can trigger crystallization, transient increases in dOH may temporarily enhance the surface reactivity of icy grains, promoting the synthesis of key species such as H_2_, CO_2_, H_2_CO, CH_3_OH, and others, and potentially opening or accelerating reaction pathways that are inefficient on fully amorphous or fully crystalline ice surfaces^59,60^.

## Methods

### Materials and ice film preparation

Single-crystal Ru(0001), Cu(111), and Au(100) substrates (Mateck GmbH) were mounted via spot-welding or mechanical clamping to tantalum wires at their edges. The crystals were resistively heated and cryogenically cooled using liquid nitrogen. Sample temperature was monitored using a chromel-alumel (K-type) thermocouple spot-welded directly to the edge of the crystal, ensuring accurate thermal control throughout the experiments. Surface cleaning was achieved through repeated cycles of Ar^+^ sputtering (1 kV) followed by annealing under UHV conditions. Typical annealing temperatures were approximately 1300 K for Ru(0001), 770 K for Cu(111), and 800 K for Au(100), yielding atomically clean and well-ordered surfaces.

High-purity D_2_O (Sigma-Aldrich, > 99.96% D; Meryer, > 99.9% D) was used in all experiments and further purified via multiple freeze-pump-thaw cycles under vacuum. The purity of the introduced D_2_O vapor was verified using a quadrupole mass spectrometer (Hiden HAL 3 F/PIC) in the preparation chamber. Amorphous solid water (ASW) and crystalline ice (CI) films with a nominal thickness of ~100 nm were grown in situ on the clean substrates via controlled dosing through a precision leak valve. Deposition was carried out at a pressure of 2 × 10^−8^ Torr for 5000 s, corresponding to ~100 L exposure (see **Supplementary Text**). ASW films were deposited at 100 K on Ru(0001), at 105 K on Cu(111), and at 100–105 K on Au(100) substrates. CI films were grown at elevated substrate temperatures ranging from 140 K to 150 K. Isothermal crystallization experiments were performed in situ under UHV conditions. During crystallization, the chamber pressure increased slightly from ~1 × 10^−10^ torr to ~5 × 10^−10^ Torr, consistent with the slow, expected desorption behavior of thick ice films.

### Infrared reflection absorption spectroscopy (IRRAS)

In situ IRRAS experiments were conducted in a custom-designed UHV system with a base pressure of 1 × 10^−10^ torr. The system consists of a preparation chamber for sample cleaning and characterization, and a measurement chamber for IRRAS experiments. The preparation chamber is equipped with a four-grid low-energy electron diffraction (LEED) optic (OCI BDL 600IR) and a quadrupole mass spectrometer for surface structure and desorption analysis. The measurement chamber was equipped with a Bruker VERTEX 70 v Fourier-transform infrared spectrometer, connected via KBr windows. The infrared beam was incident on the sample at a grazing angle of 83°, and the reflected signal was detected using a liquid nitrogen-cooled mercury-cadmium-telluride (MCT) detector.

IRRAS spectra were recorded using both *p*- and *s*-polarized light. Time-resolved isothermal measurements were performed by rapidly annealing the ice films to the target temperatures (5 – 7 K/s) and maintaining them isothermally while continuously acquiring IRRAS spectra, with a 1 s interval between consecutive spectra, as set in the OPUS software. The spectral acquisition parameters were as follows: for Ru(0001), *p*-IRRAS spectra were collected using 30 scans at a 20 kHz scan speed and 2 cm^−1^ resolution (~27 s per spectrum), while *s*-IRRAS spectra were collected using 60 scans under the same speed and resolution (~54 s per spectrum). For Cu(111) and Au(100), the spectra were recorded using 256 scans at a 40 kHz scan speed and 4 cm^−1^ resolution (~61 s per spectrum). The sample temperature was stabilized within ± 0.05 K throughout all measurements. Analysis of the vibrational features, including baseline correction (see **Supplementary Text**), peak position, integrated intensity, and FWHM, was carried out using OPUS and Origin software. The high signal-to-noise ratio of our custom IRRAS setup (~10^−5^) enables reliable detection of the *v*_free-OD_ band, which is intrinsically extremely weak compared with the bulk *v*_OD_ stretching mode^30^.

### Optical and Raman microscopy

The morphological evolution during ASW-to-CI transition was tracked using optical microscopy (Leica DM2700 M), equipped with an Olympus LMPlanFLN objective lens and a Daheng MER-502-79U3M camera for real-time image acquisition. A cold stage (Linkam THMS600) was first evacuated, and the Si/SiO_2_ substrate was cooled to 83 K. Water vapor was then introduced into the chamber to deposit a thin, optically transparent ice film (Supplementary Fig. 13). Crystallization was induced by stepwise annealing at 123 K, 173 K, and 223 K with a ramp rate of 2.5 K/s. At each temperature, the film was held for 20 min, and the representative images were captured. Over time, dark-contrast crystalline domains emerged, marking the transition from amorphous to crystalline phases. After annealing, the sample was cooled back to 83 K for Raman analysis.

Raman spectroscopy was employed to identify the phase state of the ice films observed in optical images (Supplementary Fig. 13). Measurements were performed with a confocal Raman system (Zolix RTS2) offering a spatial resolution of 15 × 15 μm and a spectral resolution of 1 cm^−1^. A 532 nm laser operating at 36 mW served as the excitation source. The signal was detected with a Peltier-cooled detector (iVac 316 CCD, Andor Technology) maintained at 213 K.

### Molecular dynamics (MD) simulations

All MD simulations were conducted using LAMMPS^61^. TIP4P/Ice^62^ force field model was applied to water molecules, which reproduces a melting temperature of ~270 K at 1 bar, in reasonable agreement with the experimental value. Lennard-Jones potentials were calculated between atoms with a cutoff distance of 1 nm. Coulombic interactions within 1 nm were computed directly. Long-range interactions beyond this distance were treated using a particle-particle particle-mesh solver in reciprocal space with an accuracy of 1 × 10^−4^. Water molecules were kept rigid using the SHAKE algorithm. All simulation cells contained 3864 water molecules and employed periodic boundary conditions in all directions. Here, we constructed two systems as follows:

1. Pure ice surface system: A pure ice system was used to extract the free OH fraction on the ice surface at different temperatures. A random configuration of ice I*h* with dimensions of 4.05 × 4.4 × 8.0 nm^3^ was generated. To create the ice-vacuum interface, we included 5 nm high vacuum regions along the *z*-axis, resulting in a simulation cell of 4.05 × 4.4 × 18.0 nm^3^. NVT simulations were conducted from 120 K to 260 K in 20 K increments, using a Nose-Hoover thermostat for temperature control^63,64^. At each temperature, MD trajectories of 5 ns were performed, with the first 1 ns for system relaxation and the subsequent 4 ns for configuration analysis. By analyzing the probability distribution curves of water molecules along the *z*-axis, the locations of 18 ice bilayers can be determined, with the top and bottom layers defined as the ice surface regions (Supplementary Fig. 12c, d).
2. Ice-liquid coexistence system: An ice-liquid coexistence system was employed to monitor the evolution of the free OH fraction on the ice surface during crystallization. An ice slab containing 864 water molecules was placed at the center of the simulation cell. Liquid water structures containing 1500 water molecules were placed on both the top and the bottom of the ice slabs. Vacuum regions were added along the *z*-axis, resulting in a simulation cell of 4.05 × 4.4 × 18.0 nm^3^. (Fig. 3c, Supplementary Fig. 11). To rigorously determine the instantaneous position of the outer ice surface where free OH groups reside, we computed the probability density profile of oxygen atoms along the surface-normal direction and fitted it using a standard hyperbolic-tangent interface model^65^ (Supplementary Fig. 12a, b). NVT simulations at 250 K and 260 K were carried out using the Nose-Hoover thermostat and run for 50 ns until complete freezing occurred.

Hydrogen bonds (HBs) in the MD simulations were identified using a commonly accepted geometrical criterion (Supplementary Fig. 22)^66^: (i) the distance between the donor and acceptor oxygen atoms is less than 3.3 Å, and (ii) the angle between the donor O–H bond and the O···O vector (H–O···O) is less than 37°. From the trajectory data, each OH group was examined against all oxygen atoms within 3.3 Å. A free OH was defined as an OH group that did not satisfy the HB criterion with any neighboring molecule. The surface free OH fraction was calculated as the ratio of non-hydrogen-bonded OH groups to the total number of surface OH groups.

## Supplementary information

Supplementary Information

Transparent Peer Review file

## Data Availability

The data generated in this study have been deposited in Figshare (10.6084/m9.figshare.30939518).

## Code availability

All the codes are provided in this paper.
