# Supplementary material for: Formation of excess dangling OH bonds during crystallization of amorphous solid water
Source: Nat Commun. 2026 May 14;17:6420. doi: 10.1038/s41467-026-73221-x (PMC13376655; doi:10.1038/s41467-026-73221-x)
Supplement: Supplementary file 1 — Supplementary Information [file 41467_2026_73221_MOESM1_ESM.pdf]

Supplementary Information for

Formation of excess dangling OH bonds during crystallization of amorphous solid water

Linbo Li,[1†] Min Lin,[2†] Yitian Cao,[1] Haihong Zheng,[1] Jianghui Liu,[2] Yibing Wang,[1] Jiani Hong,[3] HongYing Mao,[1] Haishan Cao,[2*] Jian-Qiang Zhong[1*]

[1]School of Physics, Hangzhou Normal University, Hangzhou, Zhejiang, 311121, China
[2]Key Laboratory for Thermal Science and Power Engineering of Ministry of Education, Department of Energy and Power Engineering, Tsinghua University, Beijing, 100084, China
[3]International Center for Quantum Materials, School of Physics, Peking University, Beijing, 100871, China

[†]These authors contributed equally to this work
[*]Corresponding authors. Email: haishancao@tsinghua.edu.cn; zhong@hznu.edu.cn

Supplementary Information contains:

Supplementary Text. 1 – 9
Supplementary Figs. 1 – 22
Supplementary Tables. 1 – 2
Code used in MD simulations

Supplementary Text

1. Vibrational modes in IRRAS spectra and determination of their intensities

The IRRAS spectra of ice films exhibit three principal classes of vibrational modes: stretching, bending, and librational (rotational) (see **Supplementary Fig. T2a**). Among these, the OD stretching mode (ν_{OD}, 2100 – 2800 cm^{-1}) is the most intense and structurally informative [1]. This mode arises from the stretching vibrations of OD bonds in D_2O molecules and is highly sensitive to the local hydrogen-bonding environment. Consequently, the ν_{OD} region serves as a powerful probe of key structural features of the ice films, including the phase transitions from ASW to CI, the degree of bulk ordering, and the presence and evolution of surface dangling OD bonds (dOD). The pronounced structural dependence of the ν_{OD} mode originates from hydrogen bonding induced changes to both the OD bond strength and the associated transition dipole moment [2,3].

For quantitative analysis, we use the integrated band area rather than the peak height, as the integrated area is directly proportional to the total number of vibrating dipoles [4]. This approach enables a more reliable assessment of the molecular population and film thickness.

IRRAS spectra were acquired over a range of substrate temperatures. As the reflectivity of metal substrates such as Ru(0001), Cu(111), and Au(100) can exhibit slight temperature-dependent variations, it is important to assess whether such changes affect the measured signal intensity. To this end, we conducted a calibration of the substrate reflectivity as a function of temperature in the absence of ice films. The results show that the temperature-induced variation in reflectivity is minimal; for example, the difference in signal intensity between 100 K and 160 K is approximately 1.5%. This confirms that the observed changes in IRRAS intensity during temperature-dependent measurements are dominated by modifications in film thickness and molecular structure, rather than by artifacts arising from substrate reflectivity. Nevertheless, this small effect was explicitly taken into account in the analysis of the IRRAS intensities in the present study.

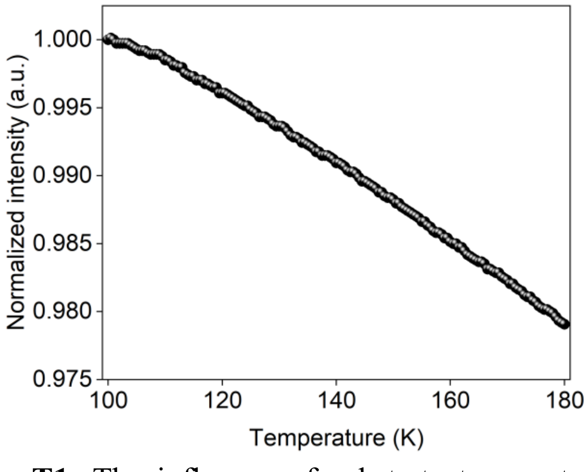

Supplementary Fig. T1. The influence of substrate temperature on the measured IRRAS signal intensity. All data are normalized to the corresponding intensity measured at 100 K.

2. Background correction procedures applied to the IRRAS spectra

The rubberband baseline correction used in this work is not a home-built routine but the standard implementation provided in the OPUS software package (Bruker Optics GmbH), which was used to process the IRRAS spectra. This method is widely employed in vibrational spectroscopy for removing slowly varying background signals [5-7].

The method estimates the baseline by constructing the lower convex envelope of the measured spectrum. Conceptually, the procedure can be visualized as stretching an elastic band underneath the spectrum such that it touches the lowest points of the data; the resulting curve defines the estimated baseline.

In the OPUS implementation, the parameter referred to as the “Number of baseline points” controls the density of baseline points used to construct the rubberband baseline across the spectrum. This parameter effectively determines how finely the spectral range is segmented when calculating the convex envelope. According to the OPUS documentation, recommended values typically range from 10 to 200, with a default value of 64. Increasing this value increases the density of baseline points and can provide a more detailed baseline representation for spectra with complex background variations.

However, the effective influence of this parameter also depends on both the number of data points in the spectrum and the spectral shape. First, setting a “Number of baseline points” value larger than 200 or smaller than 10 produces essentially the same baseline as using the boundary values of 200 or 10, respectively. Second, when the raw spectrum is relatively smooth, as is the case for the ν_{OD} band in our data, though not for the $\nu_{free\text{-}OD}$ band, the choice of “Number of baseline points” has only a minor influence on the resulting baseline. Importantly, the positions of these baseline points are determined automatically by the algorithm based on the spectral shape, rather than being manually selected by the user.

An advantage of this approach is that the baseline is determined from the global geometric structure of the spectrum, rather than from local fitting of individual peak shapes. Consequently, slowly varying background contributions (e.g., broad absorption bands or instrumental drift) are captured by the baseline envelope, while sharper vibrational features remain largely unaffected. This reduces the risk of distorting narrow spectral peaks compared with polynomial or aggressive automated fitting procedures.

To further validate the robustness of the method, we performed additional tests by varying the number of baseline points used in the convex-hull construction. These tests show that the resulting baseline changes only slightly (see **Supplementary Fig. T2d-e**). This confirms that the conclusions drawn from the spectral analysis are not sensitive to the specific parameters used in the baseline correction.

Further details of the rubberband baseline method can be found in Refs. 8 and 9.

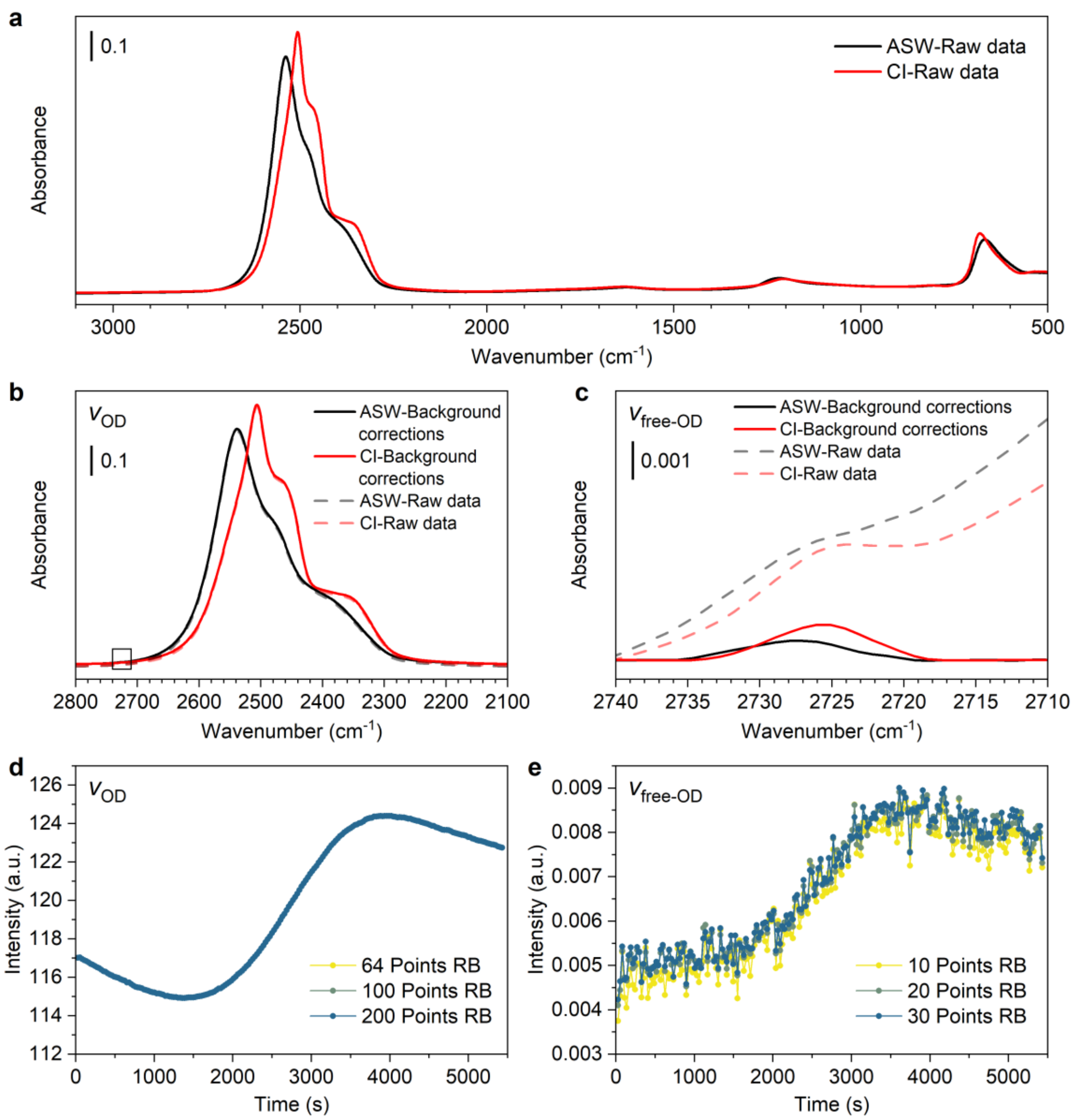

Supplementary Fig. T2. (**a**) Full IRRAS spectra of thick ASW and CI films, showing the stretching, bending and librational vibrational regions. (**b, c**) IRRAS spectra of the ν_{OD} and $\nu_{\text{free-OD}}$ regions for thick ASW and CI films before and after background corrections using the rubberband algorithm with 64 and 10 baseline points, respectively. (**d, e**) Comparison of the integrated peak areas of the ν_{OD} and $\nu_{\text{free-OD}}$ bands obtained using the rubberband algorithm with different numbers of baseline points. The spectra used in (**d**) and (**e**) are identical to those shown in **Fig. 2a** and **Fig. 3a** of the main manuscript.

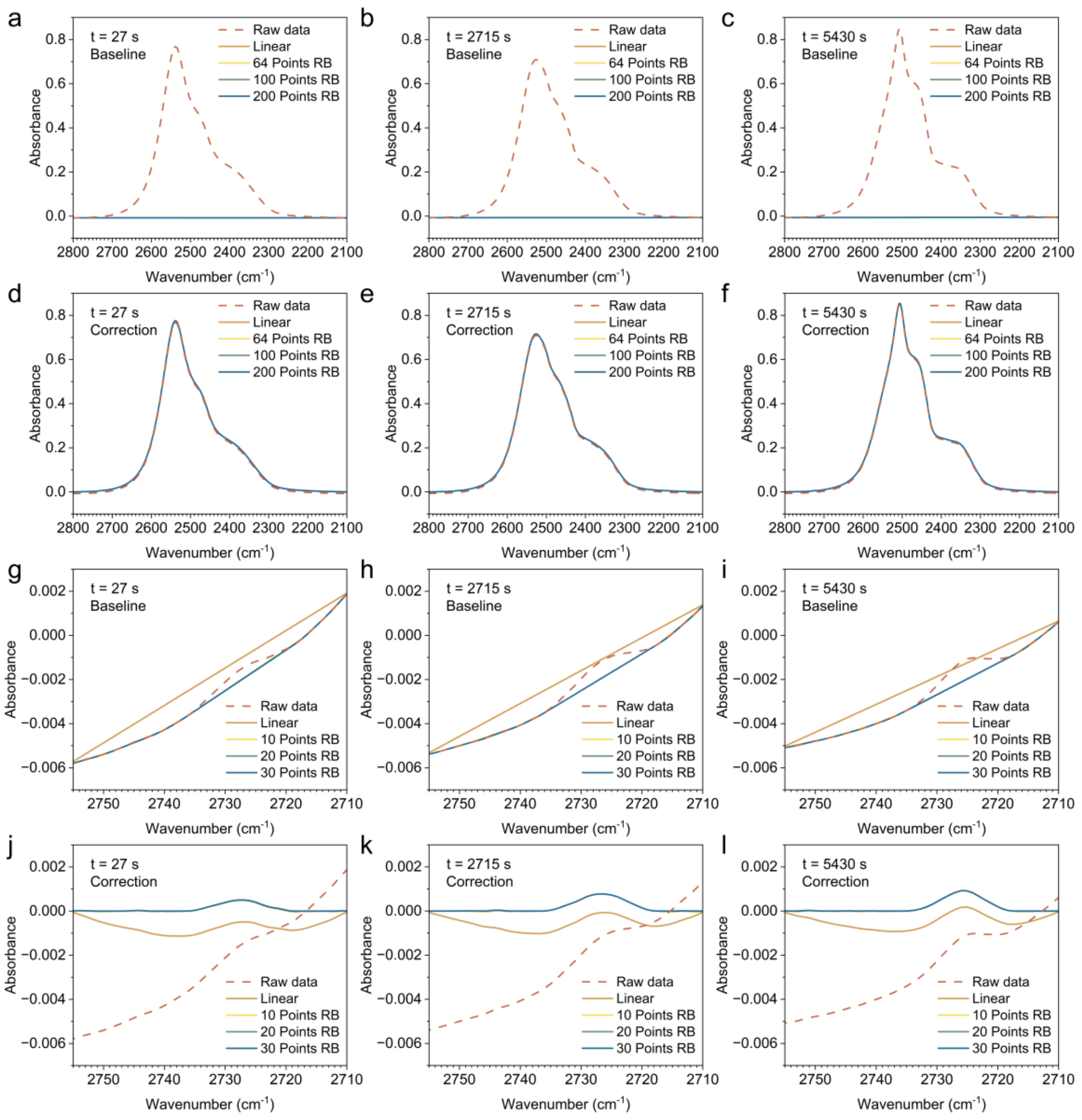

Supplementary Fig. T3. Raw and baseline-corrected IRRAS spectra of ice films at three representative phase-transition time points (before, during, and after crystallization). (**a** – **c**) Raw spectra in the ν_{OD} region with the different baseline treatments. (**d** – **f**) Baseline-corrected spectra in the ν_{OD} region. (**g** – **i**) Raw spectra in the $\nu_{free\text{-}OD}$ region with the different baseline treatments. (**j** – **l**) Baseline-corrected spectra in the $\nu_{free\text{-}OD}$ region.

3. Calibration of ice film thickness deposited under UHV conditions

The thickness of ice films deposited under UHV conditions at cryogenic temperatures was calibrated according to the procedure outlined in our previous studies [1,10]. Film deposition was quantified in Langmuir units (1 L = 1×10^{-6} torr·s), derived from the integration of pressure-time curves recorded using pressure control software. D_2O vapor was introduced via a backfilling method, and pressure measurements were obtained using an Agilent UHV-24p ion gauge positioned ~130 mm downstream from,

and directly behind, the single-crystal substrate. No gas correction factor was applied, making the exposure values specific to our experimental setup.

As shown in the figure below, the measured IRRAS signal intensity increases linearly with D_2O exposure and can be accurately described by two linear regimes intersecting at ~0.24 L. This inflection point indicates a structural transition between the first and second adlayers, likely arising from a change in the average orientation of water molecules beyond the initial contact layer. Accordingly, an exposure of ~0.24 L corresponds to the formation of one ice bilayer (BL) of D_2O on Ru(0001), assuming a sticking coefficient of unity across all coverages. Using a nominal bilayer thickness of 0.28 nm [11], an exposure of 100 L yields an estimated ice film thickness of ~417 BL, corresponding to approximately 117 nm.

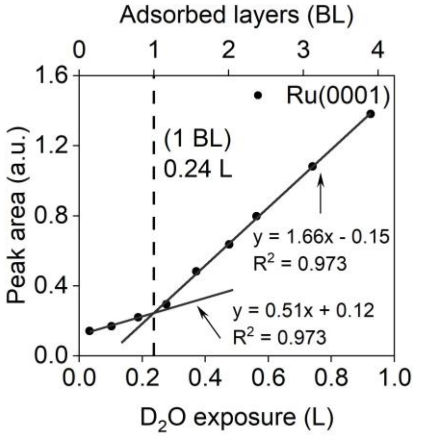

Supplementary Fig. T4. Integrated peak area of the OD stretching mode (ν_{OD}) as a function of D_2O coverage on Ru(0001). The bottom axis represents D_2O exposure in Langmuir (L), while the upper axis shows the corresponding number of adsorbed bilayers.

4. Use of different substrates in the study of excess dangling OD bonds during ASW crystallization

To establish the universality of excess dangling OD bond formation during ASW crystallization, ASW films were also prepared on Cu(111) and Au(100) substrates (**Supplementary Figs. 1** and **6 – 9**). All substrates exhibit the same qualitative behavior, demonstrating that the phenomenon is independent of substrate hydrophilicity or crystallographic orientation.

Nevertheless, different substrates generate ASW films with different structural properties, such as the morphology, porosity, and interfacial order, due to differences in substrate-water interactions and deposition dynamics [12-14]. As extensively summarized by Tonauer et al.,[15] crystallization kinetics in vapor-deposited ASW are highly sensitive to these structural properties. Consequently, both the crystallization temperature and the characteristic timescale vary across substrates and deposition conditions. Although these substrate-induced differences influence nucleation and growth rates, they do not alter the central qualitative conclusion of this work: a transient excess of surface

dangling OD bonds consistently emerges during ASW crystallization, provided that the competition between the crystallization rate k and the stabilization rate r_{stbl} satisfies the conditions discussed in the main text.

5. Desorption rates on ASW and CI films, and the desorption behavior during isothermal crystallization

All IRRAS experiments were conducted under UHV conditions, where thermal desorption of water molecules from ice films is unavoidable, especially at elevated temperatures. A key challenge is to distinguish whether spectral intensity changes arise from physical mass loss (desorption) or from structural transformations such as crystallization. This separation is particularly critical during crystallization, where both processes occur simultaneously.

To decouple these contributions, we analyzed the time-dependent ν_{OD} intensity during isothermal annealing for pure ASW and CI films (**Supplementary Figs. 20** and **21**). The intensity decay associated with desorption was used to determine the desorption rates for ASW and CI films, denoted as $R_{des-ASW}$ and R_{des-CI}, respectively.

During isothermal crystallization, the overall desorption rate (R_{des}) was modeled as a weighted combination of the desorption rates for pure ASW and CI [16]:

$$R_{des} = \left(1 - x(t)\right) * R_{des-ASW} + x(t) * R_{des-CI} \quad (1)$$

where $x(t)$ is the bulk crystallinity at time t. The cumulative IRRAS intensity loss, proportional to the total amount of water desorbed, is then expressed as:

$$I_{des} = \int \left[\left(1 - x(t)\right) * R_{des-ASW} + x(t) * R_{des-CI}\right] dt \quad (2)$$

This framework enables a quantitative description of desorption kinetics as a function of dynamically evolving crystallinity and provides a rigorous means to disentangle mass loss from structural transformations during ice phase transitions (**Supplementary Fig. 10a**).

6. Effect of bulk crystallinity on IRRAS intensity

Bulk crystallinity significantly influences IRRAS signal intensity. In this study, we examined how the phase transition from ASW to CI influences the IRRAS signals under isothermal conditions. The observed enhancement in signal intensity during crystallization is primarily attributed to structural reorganization within the film, including reorientation of vibrational dipoles and changes in the film's dielectric properties as molecular ordering increases. These structural modifications alter the film's optical response to incident infrared light, thereby affecting the measured signal.

To quantitatively describe this effect, we introduce an enhancement factor, $\alpha(t)$, as the ratio of the IRRAS intensity of an ice film with a crystallinity of $x(t)$ at time

t to that of amorphous ice with equivalent thickness:

$$I_{CI}(t) = \alpha(t) \cdot I_{ASW} \qquad (3)$$

To isolate structural effects from thickness-related signal loss due to desorption, we corrected the experimentally measured IRRAS intensities using the desorption profile derived from **Supplementary Eq. 2**. This correction ensures that changes in intensity reflect intrinsic structural transformations rather than mass loss.

As shown in **Supplementary Fig. 10b**, we derive an empirical relationship between the enhancement factor and the bulk crystallinity $x(t)$ for ice films grown on the Ru(0001) substrate:

$$\alpha_{\mathrm{Ru}}(t) = 1 + 0.13 \cdot x(t) \qquad (4)$$

This relationship indicates that, for films of constant thickness, complete crystallization from ASW to CI results in a ~13% increase in IRRAS signal intensity.

Analogous empirical relationships were obtained for ice films grown on Cu(111) and Au(100) substrates, yielding $\alpha_{\mathrm{Cu}}(t) = 1 + 0.08 \cdot x(t)$ and $\alpha_{\mathrm{Au}}(t) = 1 + 0.10 \cdot x(t)$, respectively.

7. Estimation of the free energy difference from desorption rates

During isothermal crystallization, ΔG can be quantitatively estimated using the measured thermal desorption rates of ASW ($R_{des-ASW}$) and CI films (R_{des-CI}) according to the relation [16]:

$$\Delta G = RT\mathrm{Ln}\left(\frac{R_{des-ASW}}{R_{des-CI}}\right) \qquad (5)$$

where R is the gas constant and T is the absolute temperature. Using this approach, ΔG at 155 K was determined to be 973 J mol^{-1} (**Supplementary Figs. 20** and **21**).

8. Identification of the water-vacuum interface

To rigorously determine the instantaneous position of the outer surface where free OH groups reside, we computed the probability density profile of oxygen atoms along the surface-normal direction, i.e., along z-axis, yielding $\rho(z)$ (**Supplementary Fig. 12a** and **12b**). The $\rho(z)$ profile was fitted using a standard hyperbolic-tangent interface model [17]:

$$\rho(z) = \rho_0\left(\tanh\left(\frac{z - z_1}{\delta_1}\right) - \tanh\left(\frac{z - z_2}{\delta_2}\right)\right) \qquad (6)$$

where ρ_0 is the average probability density of bulk liquid water, z_1 and z_2 are the center positions of the bottom and top water-vacuum interface, δ_1 and δ_2 represent the corresponding interfacial thicknesses. Water molecules with z-coordinates less than $z_1 + \delta_1$ or greater than $z_2 - \delta_2$ were identified as surface molecules. Free OH groups were counted exclusively within this surface region.

This procedure provides a well-defined, instantaneous surface that fluctuates dynamically and is appropriate for quantifying free OH groups exposed to vacuum.

9. General conditions for the formation of excess dOD during the ASW-to-CI transition

The surface structure of ASW films can vary significantly depending on their thermal history, whereas the surface structure of fully crystallized CI films is considerably more defined and reproducible. This distinction has been demonstrated both by our IRRAS measurements and by previous SFG studies on the temperature-dependent surface structure of CI films [18].

The excess dOD population is defined as the maximum transient dOD intensity observed during the ASW-to-CI transition relative to an appropriate reference dOD intensity. Specifically, when the initial dOD population of the ASW film is lower than that of the final CI surface at the isothermal crystallization temperature, the excess dOD is defined as the difference between the maximum transient dOD intensity and the final CI dOD intensity. Conversely, when the initial ASW dOD population exceeds that of the final CI surface, the excess dOD is defined as the difference between the maximum transient dOD intensity and the initial ASW dOD intensity.

As discussed in the main text, the emergence of excess dOD during the ASW-to-CI transition is governed by the kinetic competition between surface stabilization and crystallization. Pronounced excess dOD formation occurs when the ratio of the surface stabilization rate to the crystallization rate, $\frac{r_{stbl}}{k}$, falls below a critical threshold. Specifically, excess dOD formation is observed when $\frac{r_{stbl}}{k} < \sim 260$ at 150 K (**Supplementary Fig. 19a**), $\frac{r_{stbl}}{k} < \sim 180$ at 155 K (**Fig. 4g**), and $\frac{r_{stbl}}{k} < \sim 80$ at 160 K (**Supplementary Fig. 19b**). These differences arise from the distinct temperature dependences of the stabilization rate $r_{stbl}(T)$ and the crystallization rate $k(T)$, which are further influenced by the detailed structural properties of the initial ASW films.

Several key features can be extracted from **Fig. 4i** (see also **Supplementary Fig. T5**). First, the magnitude of excess dOD formation during the ASW-to-CI transition spans a broad range, primarily bounded by the solid and hollow diamonds. This wide range mainly reflects variations in the initial surface configurations of the ASW films.

Second, for a given initial ASW surface configuration, the maximum excess dOD occurs at temperatures for which the initial dOD fraction of the ASW film is closest to that of the final CI surface.

Third, when examined at a fixed isothermal crystallization temperature, the magnitude of the transient excess dOD depends strongly on the initial surface configuration of the ASW film. Based on the relative magnitudes of the initial ASW and final CI surface dOD populations, three temperature regimes can be identified. Approximate boundaries at ~138 K and ~155 K are inferred from **Supplementary Fig. T5** as empirical divisions between distinct regimes of excess dOD behavior; these temperatures correspond to the maxima in the excess dOD fraction associated with the ASW films exhibiting specific surface configurations, as indicated by the solid and hollow diamonds. In the low-temperature regime (< ~138 K), where the initial ASW dOD population always exceeds that of the final CI surface, ASW films with higher

initial dOD fractions exhibit smaller excess dOD formation during crystallization. In the intermediate temperature regime (138 – 155 K), where the initial ASW dOD population becomes comparable to that of the final CI surface, the maximum excess dOD occurs when the initial ASW and final CI dOD populations are approximately equal. In the high-temperature regime (> ~155 K), where the initial ASW dOD population is always lower than that of the final CI surface, ASW films with higher initial dOD fractions exhibit larger excess dOD formation during crystallization.

Finally, at very low temperatures crystallization becomes exceedingly slow; for example, at ~130 K crystallization may require up to about 10^8 s, and below ~110 K crystallization is kinetically suppressed, resulting in the absence of excess dOD formation. At the opposite extreme, at sufficiently high temperatures both crystallization and surface stabilization proceed rapidly, again suppressing the formation of excess dOD.

In conclusion, the formation of excess dOD during the ASW-to-CI transition can be regarded as a universal phenomenon, provided that the kinetic competition between crystallization and surface stabilization satisfies the conditions outlined in the main text.

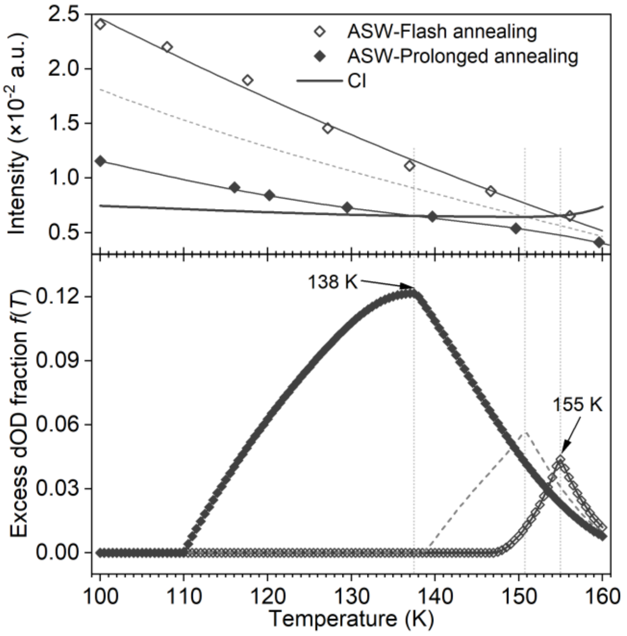

Supplementary Fig. T5. Illustration of the magnitude of transient excess dOD formed during the ASW-to-CI transition as a function of isothermal crystallization temperature and the detailed initial surface configuration of the ASW film. The curves in the upper panel are polynomial fits to guide the eye, given by:

$y_{ASW-Flash} = 1.044 \times 10^{-6} \cdot x^2 - 5.957 \times 10^{-4} \cdot x + 7.376 \times 10^{-2}$,

$y_{ASW-Prolonged} = -2.682 \times 10^{-8} \cdot x^3 + 1.110 \times 10^{-5} \cdot x^2 - 1.627 \times 10^{-3} \cdot x + 9.013 \times 10^{-2}$,

$y_{CI} = 1.309 \times 10^{-15} \cdot x^8 - 1.297 \times 10^{-12} \cdot x^7 + 5.599 \times 10^{-10} \cdot x^6 - 1.378 \times 10^{-7} \cdot x^5 + 2.111 \times 10^{-5} \cdot x^4 - 2.065 \times 10^{-3} \cdot x^3 + 1.259 \times 10^{-1} \cdot x^2 - 4.370 \times 10^0 \cdot x + 6.618 \times 10^1$

The dashed line in the upper panel represents the average of the two polynomial fits, while that in the lower panel shows the corresponding extracted excess dOD fraction as a function of temperature, as discussed in the main text.

Supplementary Figures and Tables

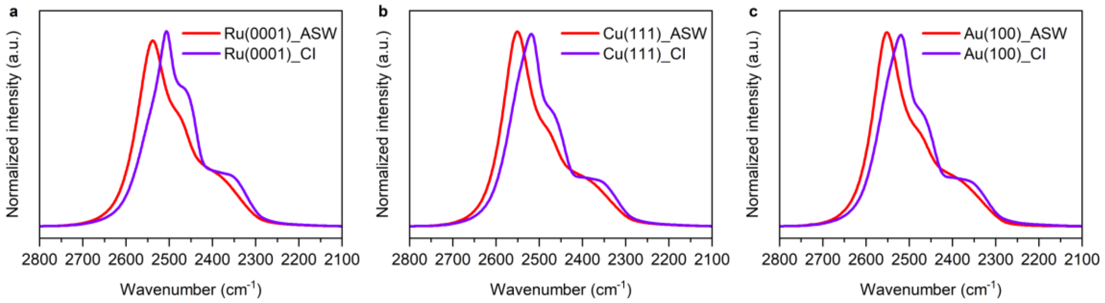

Supplementary Fig. 1. IRRAS spectra in the OD stretching region (ν_{OD}) for ASW and CI films. The spectra were recorded after depositing ~100 L D_2O onto (**a**) Ru(0001), (**b**) Cu(111), and (**c**) Au(100) substrates. ASW films were deposited at 100 K on Ru(0001) and Au(100), and at 105 K on Cu(111), respectively. CI films were obtained by annealing the ASW films at elevated temperatures to induce crystallization.

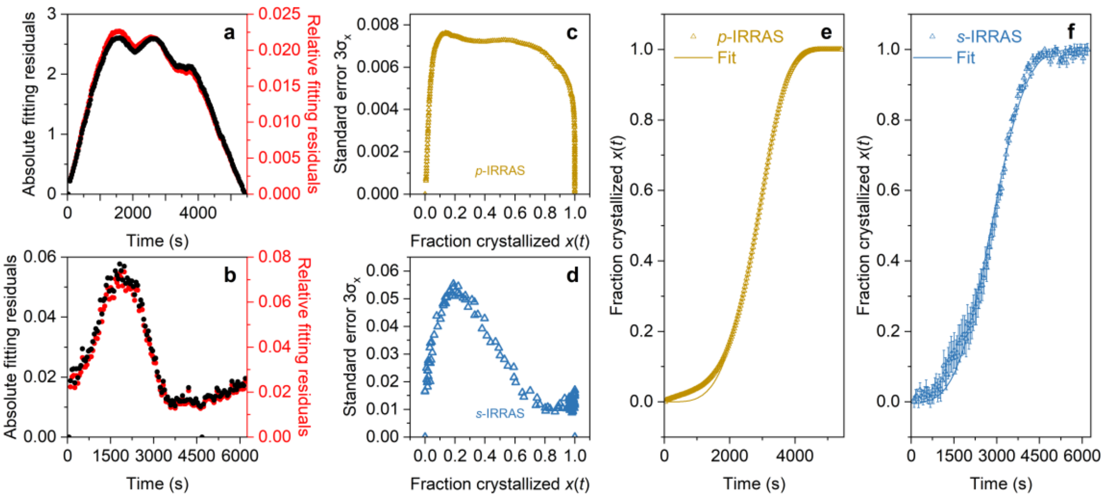

Supplementary Fig. 2. Estimation of the uncertainties in $x(t)$ and n shown in Fig. 2 of the main text. (**a, b**) Absolute and relative fitting residuals of $x(t)$ for the spectra shown in **Fig. 2a** and **2b**, respectively. (**c, d**) Standard errors of $x(t)$ obtained from fitting each intermediate spectrum using the two-state (ASW/CI) mixture model. (**e, f**) Time-dependent fraction crystallized $x(t)$ with error bars determined from the corresponding standard errors, together with the Avrami fits used to extract the Avrami exponent n.

Each intermediate spectrum was modeled as a linear combination of the ASW and CI reference spectra, i.e., $(1 - x(t))$ASW + $(x(t))$CI, where the first and last spectra in the crystallization sequence were taken as the ASW and CI references, respectively. The first spectrum corresponds to the fully amorphous state ($x = 0$), while the last spectrum corresponds to the fully crystalline state ($x = 1$). For each intermediate spectrum, the crystallinity $x(t)$ was determined by least-squares regression. The uncertainty of $x(t)$ (reported as a 3σ standard error) was calculated from the variance of the fitting residuals. It is important to note that the signal-to-noise ratio of the IRRAS measurements is sufficiently high such that the additional uncertainty associated with numerical integration of the ν_{OD} peak area is negligible. The uncertainty of the Avrami exponent n was determined by fitting the upper and lower bounds of the crystallinity, $x(t) \pm 3\sigma(t)$.

The decomposition of the ν_{OD} spectra into ASW and CI components inevitably introduces uncertainties. Nevertheless, this fitting protocol has been widely and successfully applied to analyze structural transitions in various amorphous and crystalline molecular films, including water [19-23] and other cryogenic films such as amorphous acetonitrile [24].

The primary limitation of this approach stems from the assumption of a two-state (ASW/CI) mixture model, in which each intermediate spectrum is represented as a linear combination of ASW and CI reference spectra. This assumption inherently neglects the possible existence of intermediate structures that may form during nucleation and crystal growth. According to classical crystallization theory, the interfacial region surrounding a growing crystalline nucleus cannot be strictly

categorized as either ASW or CI. Consequently, such transition structures are not captured by the two-state model, leading to increased fitting residuals, particularly during the periods of rapid crystallization. As shown in **Supplementary Fig. 2a** and **2b**, the relative fitting residuals can reach ~2.3 % for *p*-IRRAS and ~7.0% for *s*-IRRAS during these stages, reflecting the influence of such intermediate structures.

We also note that Geissler and co-workers demonstrated that the presence of isosbestic points does not necessarily imply a two-state transformation, as similar features may emerge in systems dominated by temperature-dependent continuous spectral broadening [25,26]. However, these theoretical considerations were developed for liquid water over a broad temperature range (275 – 355 K). In the present study, crystallization is performed isothermally, where temperature-induced inhomogeneous broadening is negligible. Under these conditions, the two-state approximation remains appropriate and has been extensively validated in previous analyses of ASW crystallization.

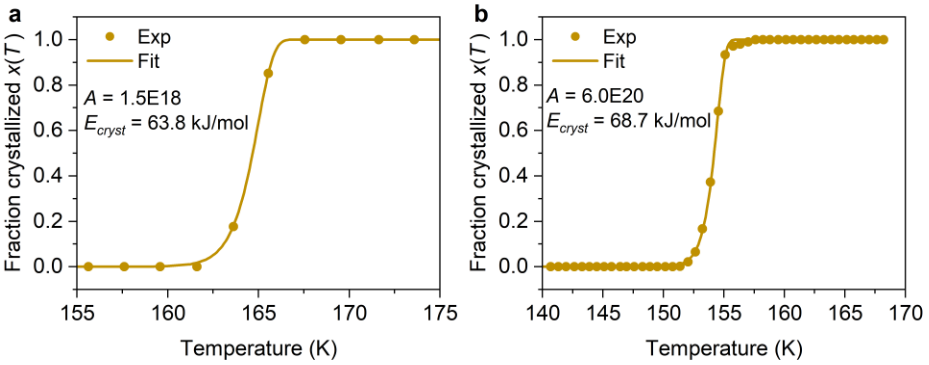

Supplementary Fig. 3. Fraction crystallized as a function of temperature for ASW films. (**a**) An ASW film prepared by depositing ~90 L of D_2O onto Ru(0001) at 100 K, followed by linear heating at 2 K/min. (**b**) An ASW film prepared by depositing ~100 L of D_2O onto Au(100) at 100 K, followed by linear heating at 0.6 K/min. The solid lines represent fits based on the Avrami equation adapted for non-isothermal crystallization.

The standard Avrami equation describes crystallization under isothermal (constant temperature) conditions. To extend this model to non-isothermal crystallization – where temperature continuously changes with time – the time-dependent term kt is replaced with a time-averaged rate constant $\langle k(t)\rangle$, yielding:

$$x(t) = 1 - \exp[-(\langle k(t)\rangle)^n] \tag{7}$$

Here, $\langle k(t)\rangle$ is defined as: $\langle k(t)\rangle = \int_0^t k(t')dt'$. This approximation is particularly appropriate for slow heating rates, where the system remains near quasi-equilibrium during crystallization.

Assuming a constant heating rate $\beta = \frac{dT}{dt}$, the time integral can be converted to a temperature integral:

$$\langle k(t)\rangle = \frac{\langle k(T)\rangle}{\beta} \tag{8}$$

With a $\langle k(T)\rangle$ expressed in Arrhenius form:

$$\langle k(T)\rangle = \int_{T_0}^{T} k(T')dT' = \int_{T_0}^{T} A\exp\left(-\frac{E_{cryst}}{RT'}\right)dT' \tag{9}$$

where A is the pre-exponential factor, E_{cryst} is the activation energy for crystallization, and R is the gas constant. Substituting this expression into the generalized Avrami equation gives the non-isothermal Avrami expression:

$$x(T) = 1 - \exp\left\{-\left[\frac{1}{\beta}\int_{T_0}^{T} A\exp\left(-\frac{E_{cryst}}{RT'}\right)dT'\right]^n\right\} \tag{10}$$

This formulation provides a quantitative framework for describing phase transitions under linear temperature ramps and serves as a foundation for kinetic analysis of non-isothermal crystallization processes.

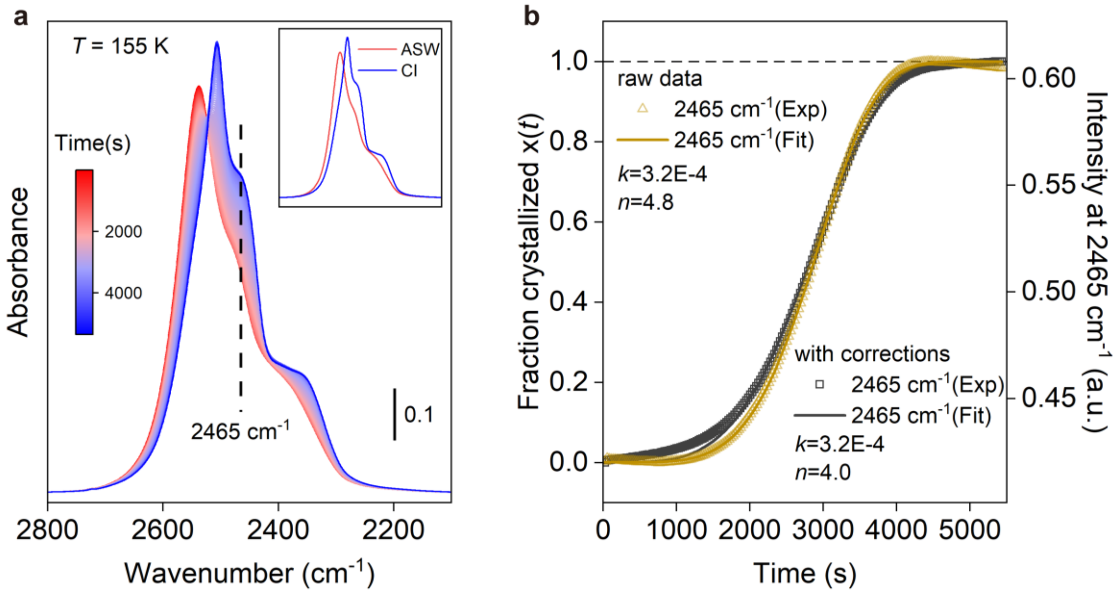

Supplementary Fig. 4. Estimation of ice film crystallinity during isothermal crystallization based on the absorbance at 2465 cm^{-1}. (**a**) Evolution of *p*-polarized IRRAS spectra in the OD stretching modes (ν_{OD}) for ~110 nm-thick ASW films on Ru(0001) during isothermal crystallization at 155 K. The red spectrum represents the initial amorphous state (100% ASW), while the blue spectrum corresponds to the fully crystallized state (100% CI). The vertical dashed line in (**a**) at 2465 cm^{-1} marks the absorbance value used to track the evolution of crystallinity over time. (**b**) Time-dependent fraction crystallized extracted from the spectra in (**a**), fitted using the Avrami equation. Both raw data and desorption-corrected data are shown for comparison.

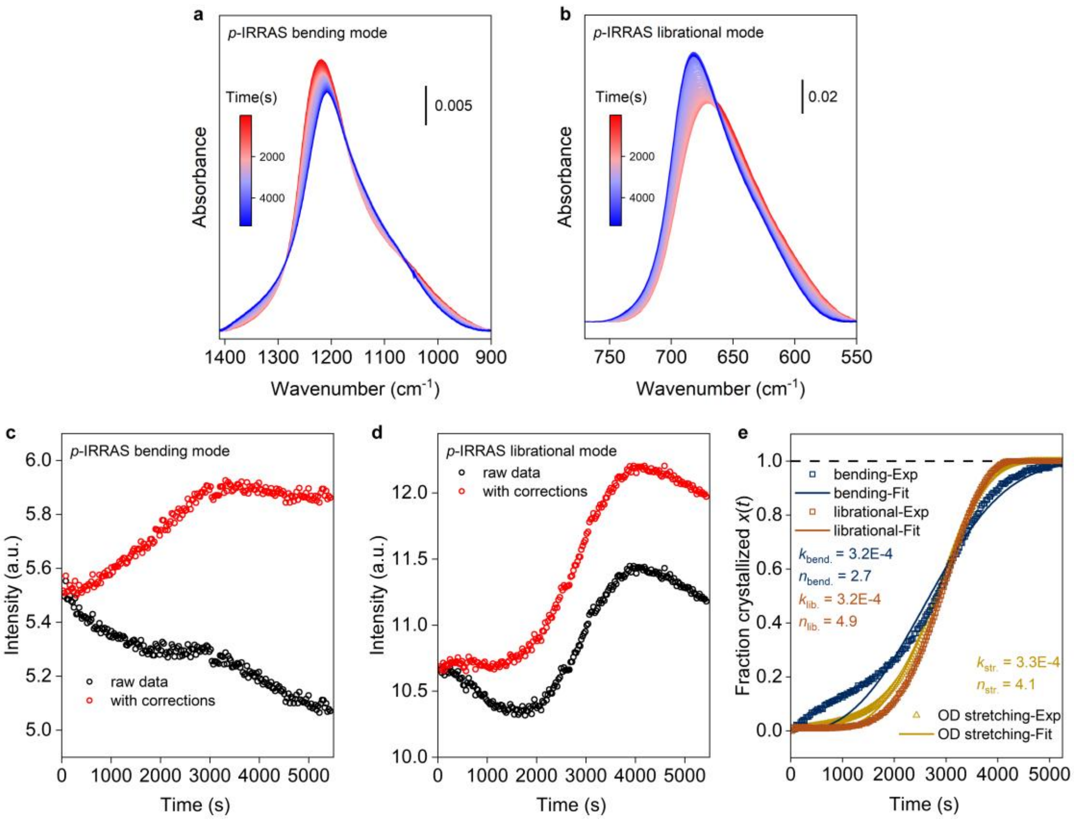

Supplementary Fig. 5. Fraction crystallized $x(t)$ extracted from IRRAS spectra of ~110 nm-thick ASW films on Ru(0001), fitted using the Avrami equation. Time-resolved IRRAS spectra of the (**a**) bending and (**b**) librational modes were recorded during isothermal crystallization. (**c**) and (**d**) show the corresponding intensity changes of these modes. (**e**) The fraction crystallized $x(t)$ was derived from the spectral evolution of each vibrational mode (stretching, bending and librational) and quantitatively fitted using the Avrami model, providing a comprehensive description of crystallization kinetics across different vibrational signatures.

These vibrational modes provide distinct yet complementary insights into the structure and dynamics of the hydrogen-bond network (HBN). The OD stretching mode primarily probes the strength and orientation of hydrogen bonds involving both surface and bulk D_2O molecules. Its lineshape and peak position are highly sensitive to the orientational distribution of OD dipoles, offering a direct measure of hydrogen-bond ordering. The bending mode, associated with angular deformations of the D-O-D bond, reflects local hydrogen-bond geometries. While less sensitive to long-range order, changes in its frequency and intensity can reveal local structural asymmetries. The librational (rotational) mode corresponds to hindered molecular reorientation and is highly responsive to the rigidity and collective interactions within the HBN. In CI phases, librational features appear sharper and more anisotropic, whereas in ASW, they are broader, indicating dynamic disorder and rotational freedom. Together, these analyses enable a detailed assessment of crystallization kinetics and reveal potential anisotropies in hydrogen-bond dynamics during the ASW-to-CI transition.

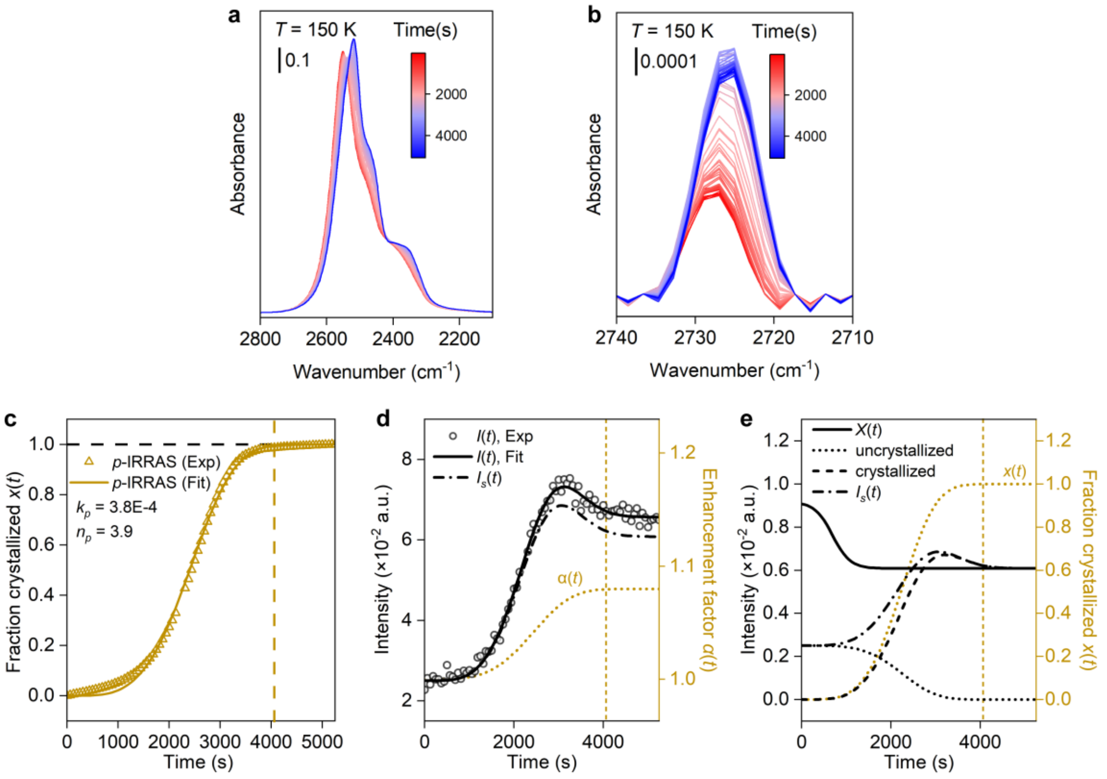

Supplementary Fig. 6. Time-resolved IRRAS analysis of crystallization kinetics for ~100 L ASW films deposited on Cu(111) at 105 K. (**a**) *p*-polarized IRRAS spectra in the OD stretching region (ν_{OD}) recorded during isothermal crystallization at 150 K. The initial (red) and final (blue) spectra correspond to the fully amorphous ($x = 0$) and fully crystalline states ($x = 1$), respectively. (**b**) Evolution of the surface-specific free OD mode ($\nu_{free\text{-}OD}$), associated with dOD, during the crystallization process. (**c**) Time-dependent fraction crystallized $x(t)$ extracted from the spectral deconvolution of (**a**), and fitted with the Avrami equation. The dashed vertical line marks the completion of bulk crystallization. (**d**) Temporal evolution of the integrated intensity of the $\nu_{free\text{-}OD}$ mode at 150 K, fitted with the model $I(t) = I_s(t) \cdot \alpha(t)$, as described in the main text. (**e**) Extraction of the surface dOD contribution $I_s(t)$ by accounting for surface stabilization effects, modeled by a logistic decay function $X(t)$.

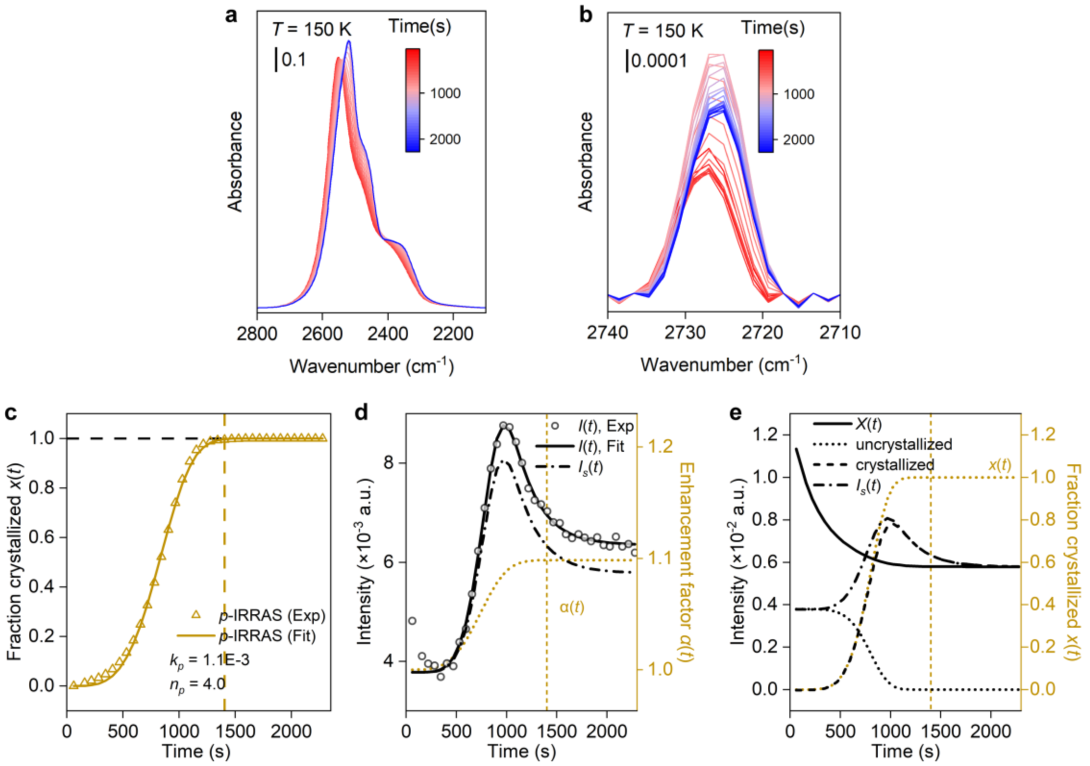

Supplementary Fig. 7. Time-resolved IRRAS analysis of crystallization kinetics for ~100 L ASW films deposited on Au(100) at 100 K. (**a**) p-polarized IRRAS spectra in the OD stretching region (ν_{OD}) recorded during isothermal crystallization at 150 K. The initial (red) and final (blue) spectra correspond to the fully amorphous ($x = 0$) and fully crystalline states ($x = 1$), respectively. (**b**) Evolution of the surface-specific free OD mode ($\nu_{free\text{-}OD}$), associated with dOD, during the crystallization process. (**c**) Time-dependent fraction crystallized $x(t)$ extracted from the spectral deconvolution of (**a**) and fitted with the Avrami equation. The dashed vertical line marks the completion of bulk crystallization. (**d**) Temporal evolution of the integrated intensity of the $\nu_{free\text{-}OD}$ mode at 150 K, fitted with the model $I(t) = I_s(t) \cdot \alpha(t)$, as described in the main text. (**e**) Extraction of the surface dOD contribution $I_s(t)$ by accounting for surface stabilization effects, modeled by a logistic decay function $X(t)$.

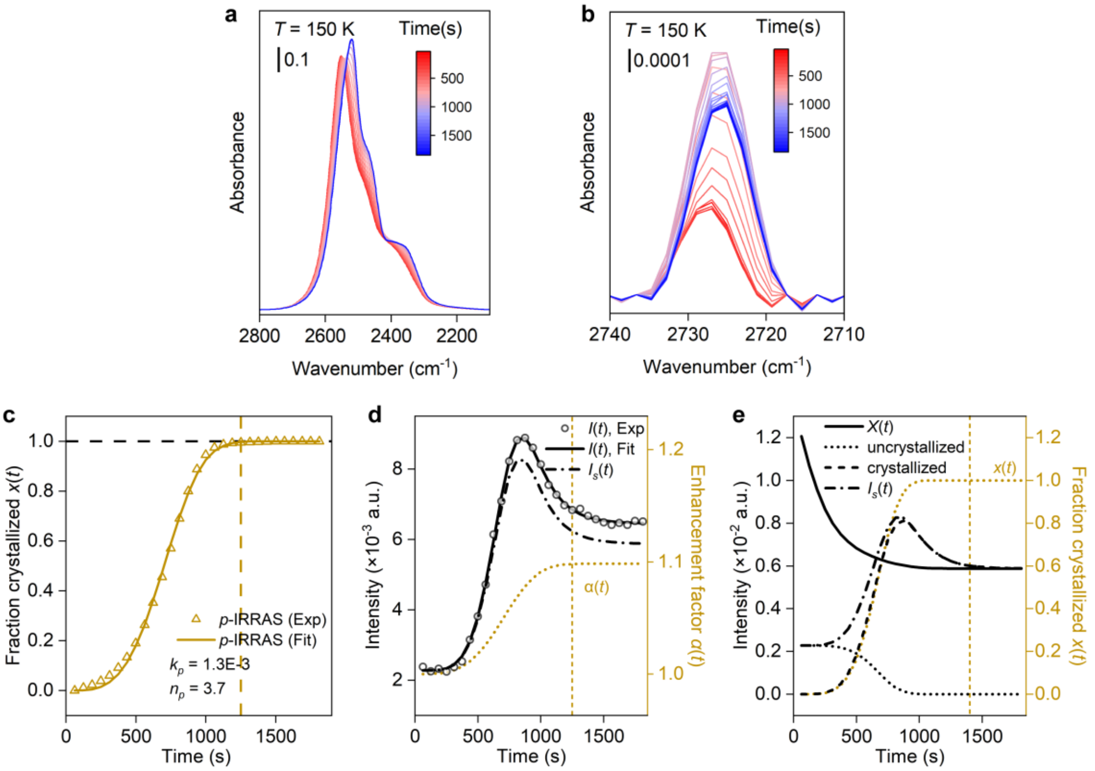

Supplementary Fig. 8. Time-resolved IRRAS analysis of crystallization kinetics for ~100 L ASW films deposited on Au(100) at 105 K. (**a**) *p*-polarized IRRAS spectra in the OD stretching region (ν_{OD}) recorded during isothermal crystallization at 150 K. The initial (red) and final (blue) spectra correspond to the fully amorphous ($x = 0$) and fully crystalline states ($x = 1$), respectively. (**b**) Evolution of the surface-specific free OD mode ($\nu_{free\text{-}OD}$), associated with dOD, during the crystallization process. (**c**) Time-dependent fraction crystallized $x(t)$ extracted from the spectral deconvolution of (**a**) and fitted with the Avrami equation. The dashed vertical line marks the completion of bulk crystallization. (**d**) Temporal evolution of the integrated intensity of the $\nu_{free\text{-}OD}$ mode at 150 K, fitted with the model $I(t) = I_s(t) \cdot \alpha(t)$, as described in the main text. (**e**) Extraction of the surface dOD contribution $I_s(t)$ by accounting for surface stabilization effects, modeled by a logistic decay function $X(t)$.

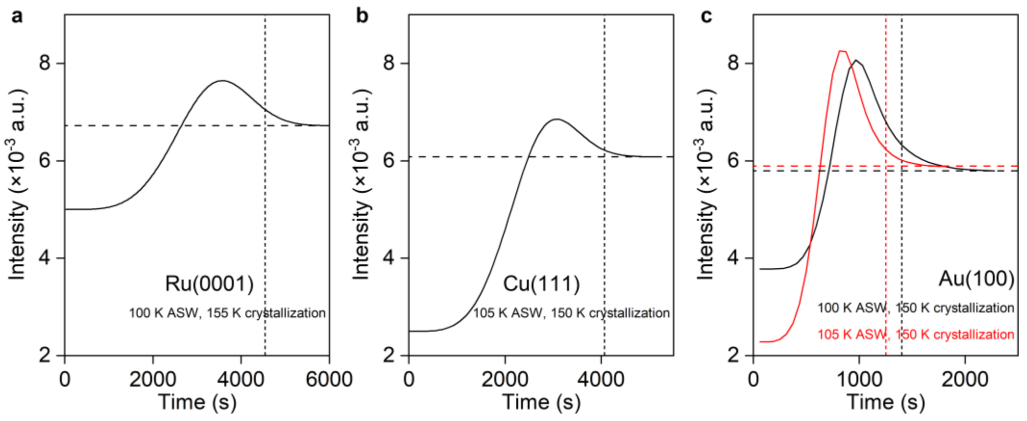

Supplementary Fig. 9. Summary of the experimentally derived surface-specific component $I_s(t)$, which is directly proportional to the dOD population, during bulk crystallization. (**a**) ASW film grown on Ru(0001) at 100 K and rapidly annealed to 155 K for isothermal IRRAS crystallization measurements. (**b**) ASW film grown on Cu(111) at 105 K and rapidly annealed to 150 K for isothermal IRRAS crystallization measurements. (**c**) ASW films grown on Au(100) at 100 K and 105 K and rapidly annealed to 150 K for isothermal IRRAS crystallization measurements. The dashed vertical line indicates the completion of bulk crystallization.

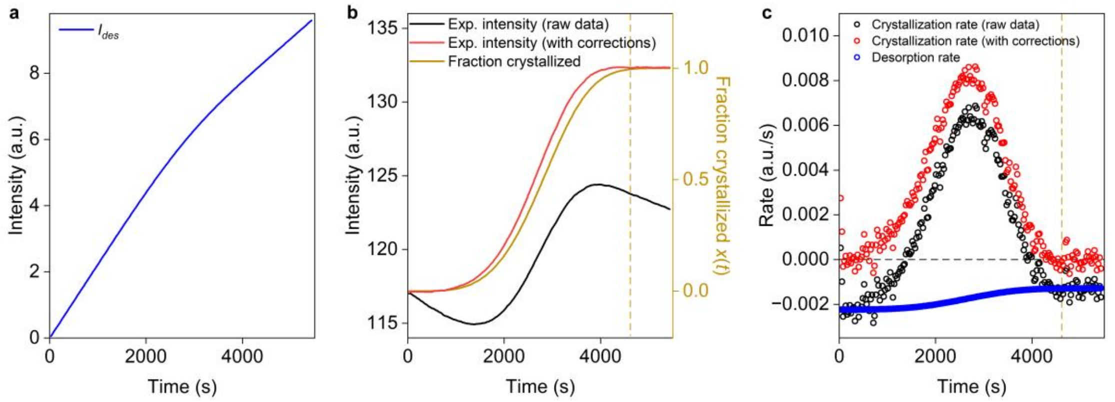

Supplementary Fig. 10. Water desorption during isothermal crystallization and its effect on IRRAS intensities for the ice film prepared on Ru(0001). (**a**) Calculated loss of IRRAS intensity in the OD stretching region (ν_{OD}) due to water desorption during isothermal crystallization, obtained from **Supplementary Eq. 2**. (**b**) Time evolution of the ν_{OD} intensity, shown together with the fraction crystallized of the ice film. The ν_{OD} intensity has been corrected for film thickness loss caused by desorption. The crystallized fraction curve is identical to that presented in **Fig. 2c**. (**c**) Temporal derivatives of the ν_{OD} intensity, representing the instantaneous rate of change in the IRRAS signal during crystallization.

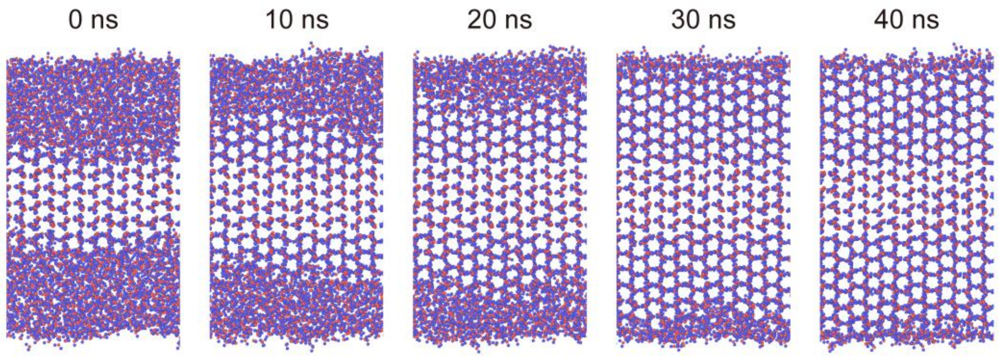

Supplementary Fig. 11. Molecular dynamics simulation of dOH evolution during ASW crystallization. Representative snapshots from MD simulations of ASW crystallizing at 250 K, illustrating the structural evolution from the amorphous to the crystalline state.

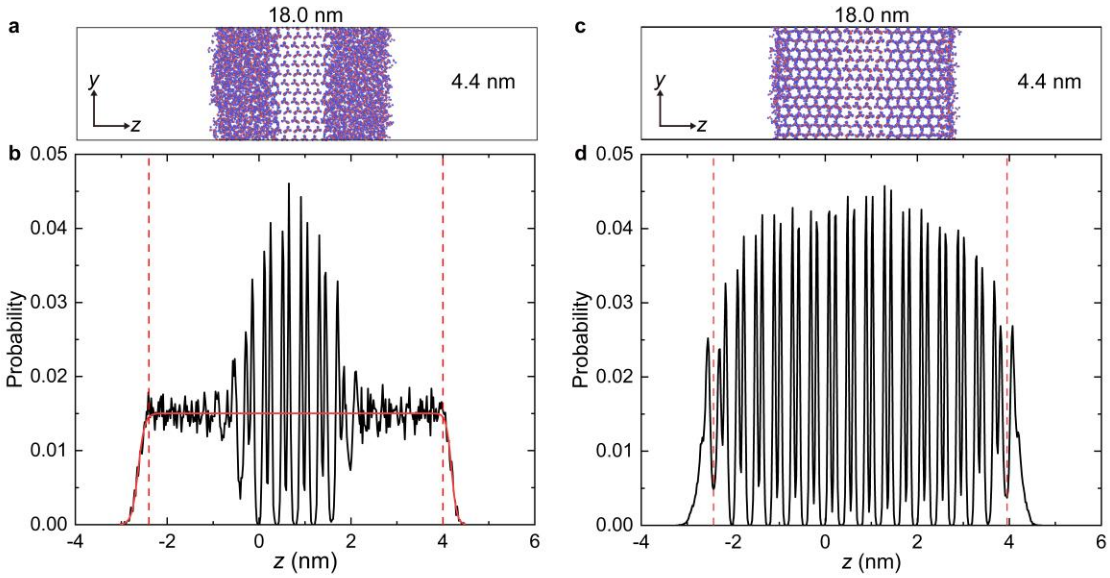

Supplementary Fig. 12. System configuration and identification of the water-vacuum (a, b) and ice-vacuum (c, d) interfaces. (**a**) Representative simulation snapshot of the liquid-ice coexistence system at 260 K. (**b**) Probability density profile of oxygen atoms along the *z*-axis. The red solid line shows the fit to the density profile, and the red dashed lines denote the positions of the bottom and top surfaces. (**c**) Representative simulation snapshot of the ice slab at 260 K. (**d**) Probability density profile of oxygen atom positions along the *z*-axis. The red dashed lines denote the boundaries of the bottom and top ice layers, used to define the extent of the surface region.

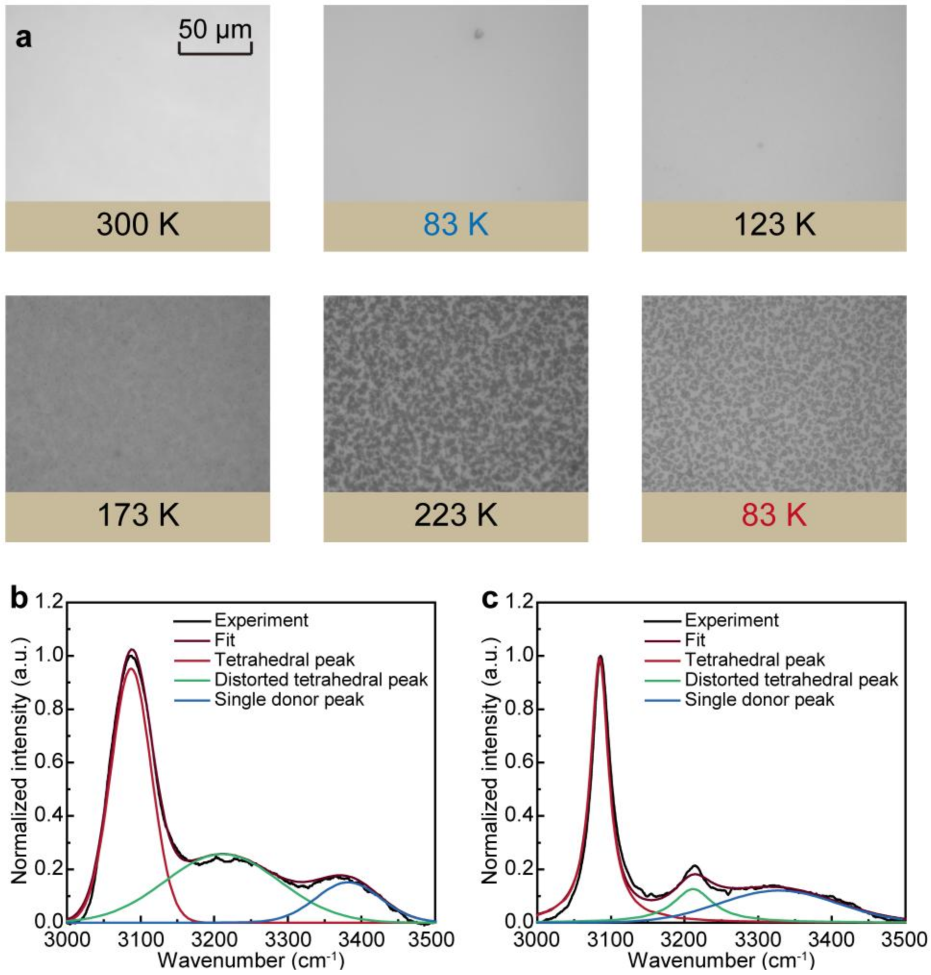

Supplementary Fig. 13. Optical and Raman characterization of the ASW-to-CI transition. (**a**) Representative optical images showing the crystallization of ASW into CI. (**b**) Raman spectrum of the ice film at 83 K before annealing (i.e., ASW). (**c**) Raman spectrum of the same ice film at 83 K after annealing (i.e., CI).

The ice film initially formed at 83 K was optically featureless. Upon annealing, distinct dark-contrast crystalline domains emerged. To identify the phase of both the featureless and dark-contrast ice, Raman spectra were acquired before and after annealing. Spectra were deconvoluted using Voigt fitting into three characteristic components: tetrahedral, distorted tetrahedral, and single-donor modes. As summarized in **Supplementary Table 2**, the pre-annealing spectrum was dominated by the distorted tetrahedral component, with a broad full width at half maximum (FWHM), consistent with the short-range disorder characteristic of ASW. Following annealing, the increased intensity of the tetrahedral peak and the concomitant narrowing of its FWHM indicate enhanced structural order and crystallinity, consistent with the formation of CI. The concurrent rise in the single-donor contribution is attributed to the increased density of grain boundaries among the emerging crystalline domains.

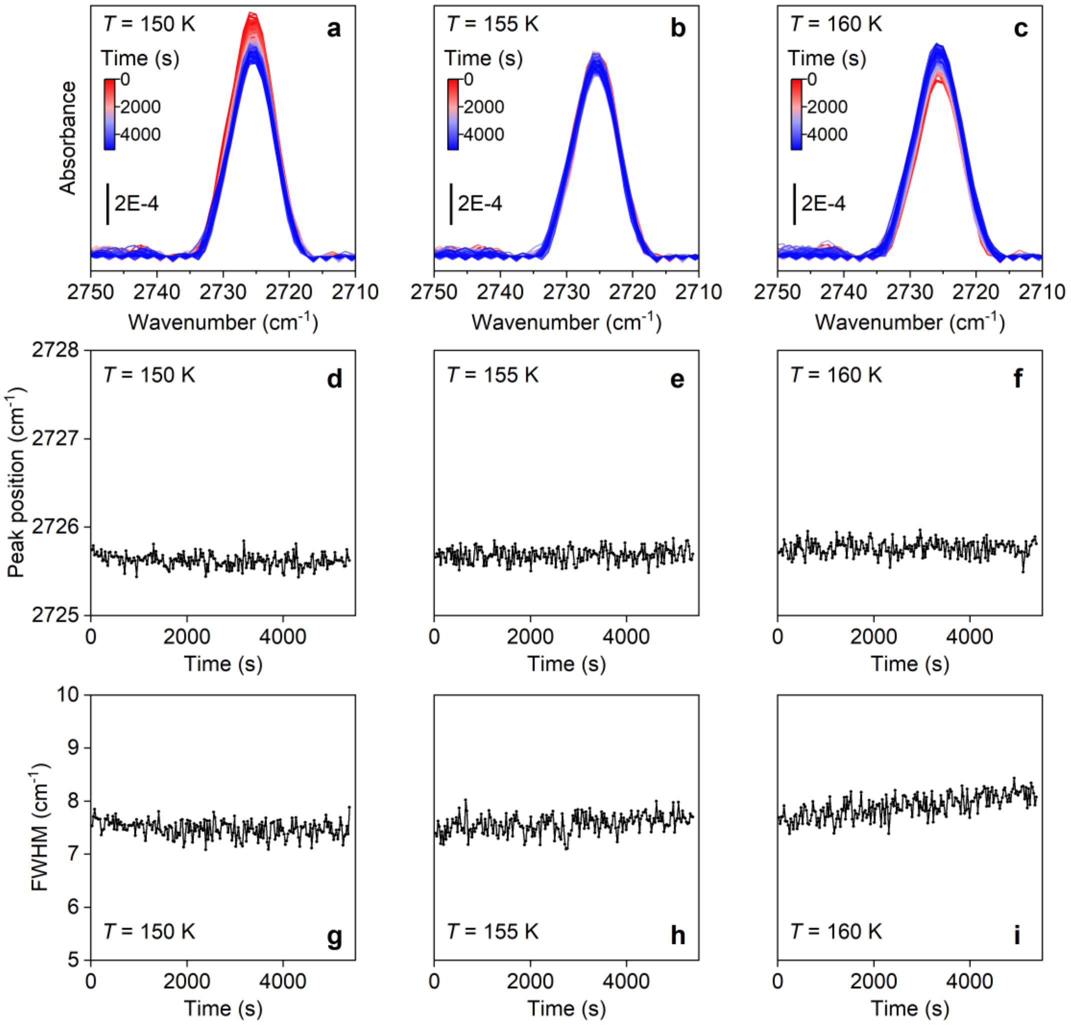

Supplementary Fig. 14. Temperature-dependent evolution of surface-specific free OD modes ($\nu_{\text{free-OD}}$) in CI films prepared on Ru(0001). (**a**) – (**c**) Time-resolved *p*-polarized IRRAS spectra of the $\nu_{\text{free-OD}}$ modes recorded at 150 K (**a**), 155 K (**b**), and 160 K (**c**). These modes correspond to surface OD groups not engaged in hydrogen bonding (i.e., dOD). (**d**) – (**f**) Temporal evolution of the $\nu_{\text{free-OD}}$ peak position at each temperature. No significant peak shift is observed within the experimental resolution, suggesting that the local vibrational environment of dOD remains relatively stable despite ongoing surface restructuring. (**g**) – (**i**) Temporal evolution of FWHM of the $\nu_{\text{free-OD}}$ modes. While the linewidth remains narrow and stable at lower temperatures, it progressively broadens at higher temperatures, especially at 160 K. This broadening is attributed to increased structural heterogeneity, reflecting enhanced surface mobility and disorder under elevated thermal conditions.

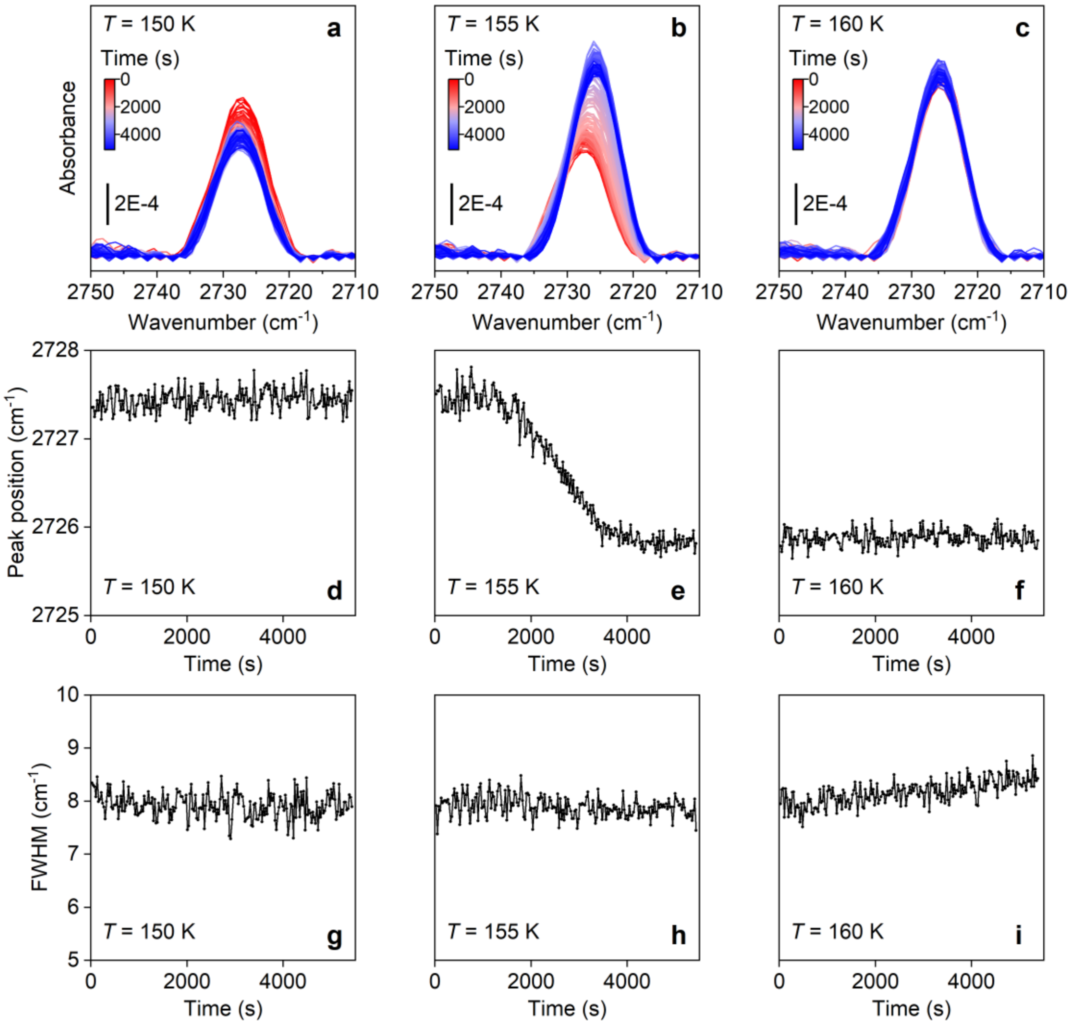

Supplementary Fig. 15. Temperature-dependent evolution of surface-specific free OD modes ($\nu_{\text{free-OD}}$) in ASW films on Ru(0001). (**a**) – (**c**) Time-resolved *p*-polarized IRRAS spectra of the $\nu_{\text{free-OD}}$ modes recorded at 150 K (**a**), 155 K (**b**), and 160 K (**c**). These modes correspond to dOD and serve as sensitive probes of the surface environment and molecular dynamics during thermal annealing and crystallization. (**d**) – (**f**) Temporal evolution of the $\nu_{\text{free-OD}}$ peak positions at each temperature. At 155 K, a pronounced redshift is observed over time, indicative of the progressive crystallization of ASW into CI. This redshift reflects the increasing influence of the evolving surface HBN on the vibrational environment of dOD. (**g**) – (**i**) Temporal evolution of the FWHM of the $\nu_{\text{free-OD}}$ modes at each temperature. At 155 K, a slight narrowing of the peak is observed, suggesting the formation of more ordered and homogeneous surface structures as crystallization proceeds. In contrast, at 160 K, the $\nu_{\text{free-OD}}$ peak exhibits progressive broadening, indicating increasing structural heterogeneity and dynamic disorder at the surface due to enhanced molecular mobility. This behavior parallels the trend observed in CI films under similar thermal conditions (see **Supplementary Fig. 14i**).

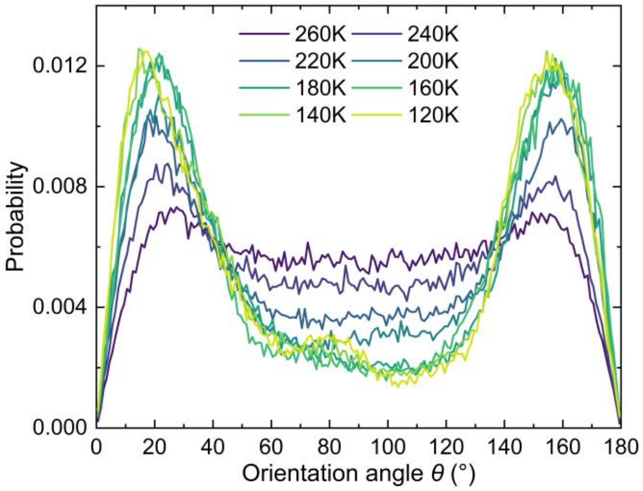

Supplementary Fig. 16. Orientation distribution of free OH groups (i.e., dOH) at the ice surface.

The orientation distribution of dOH was also analyzed. The orientation angle θ, defined as the angle between the OH bond vector and the surface normal (z-axis), narrows toward 0° and 180° at lower temperatures, suggesting an increased alignment of dOH perpendicular to the surface.

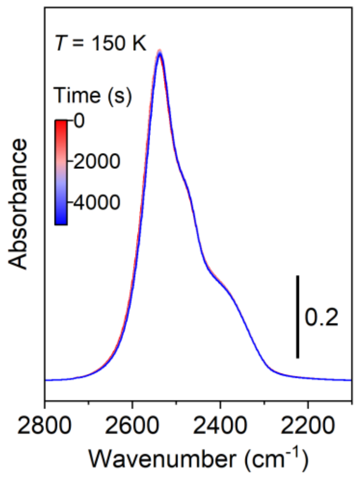

Supplementary Fig. 17. Evolution of the OD stretching modes (ν_{OD}) in thick ASW films on Ru(0001) during annealing at 150 K.

IRRAS spectra were recorded for the ASW films deposited at 100 K and subsequently annealed isothermally at 150 K for ~5000 s. No significant changes in peak intensity or spectral shape were observed, indicating that the film remains predominantly amorphous, with no substantial crystallization or mass loss over the experimental timescale.

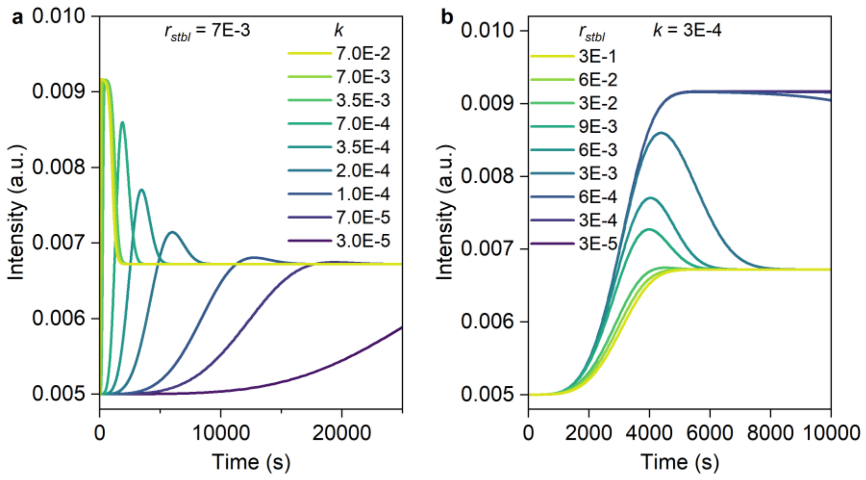

Supplementary Fig. 18. Simulated impact of the relative rates of surface stabilization (r_{stbl}) and crystallization (k) on excess dOD formation during isothermal crystallization of ASW films at 155 K. (**a**) Simulations were performed with r_{stbl} fixed at 7×10^{-3} s^{-1} while varying the crystallization rate constant k from 7×10^{-2} to 3×10^{-5} s^{-1}. (**b**) Simulations were performed with k fixed at 3×10^{-4} s^{-1}, and r_{stbl} varied from 3×10^{-5} to 3×10^{-1} s^{-1}. Excess dOD emerges only when the ratio $\frac{r_{stbl}}{k}$ falls below ~180, underscoring the competition between surface relaxation and crystallization kinetics in governing the transient appearance of surface dOD.

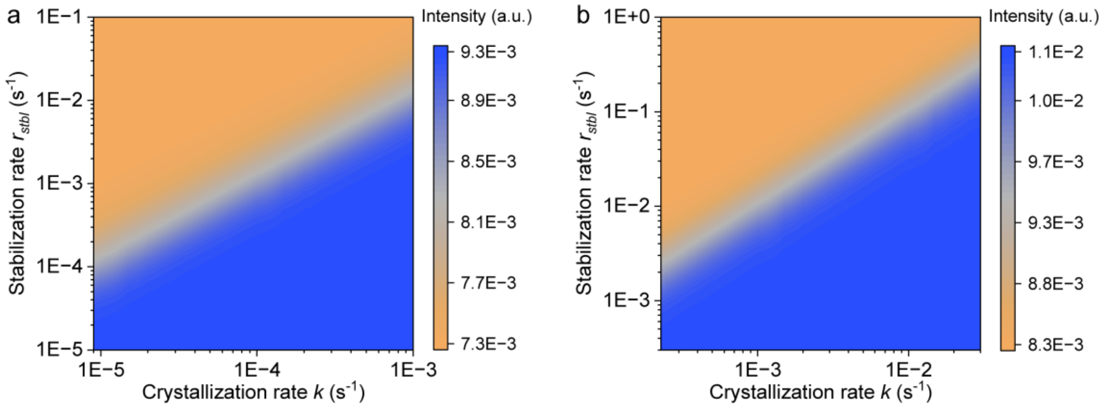

Supplementary Fig. 19. Simulated influence of the relative rates of surface stabilization (r_{stbl}) and crystallization (k) on excess dOD formation during isothermal crystallization of ASW films. (**a**) Simulations performed at 150 K. (**b**) Simulations performed at 160 K.

At both temperatures, the emergence of excess dOD is governed by the competition between surface stabilization and crystallization kinetics. Significant excess dOD formation occurs when the ratio $\frac{r_{stbl}}{k}$ falls below a critical threshold, specifically, when $\frac{r_{stbl}}{k} < \sim 260$ at 150 K and $\frac{r_{stbl}}{k} < \sim 80$ at 160 K.

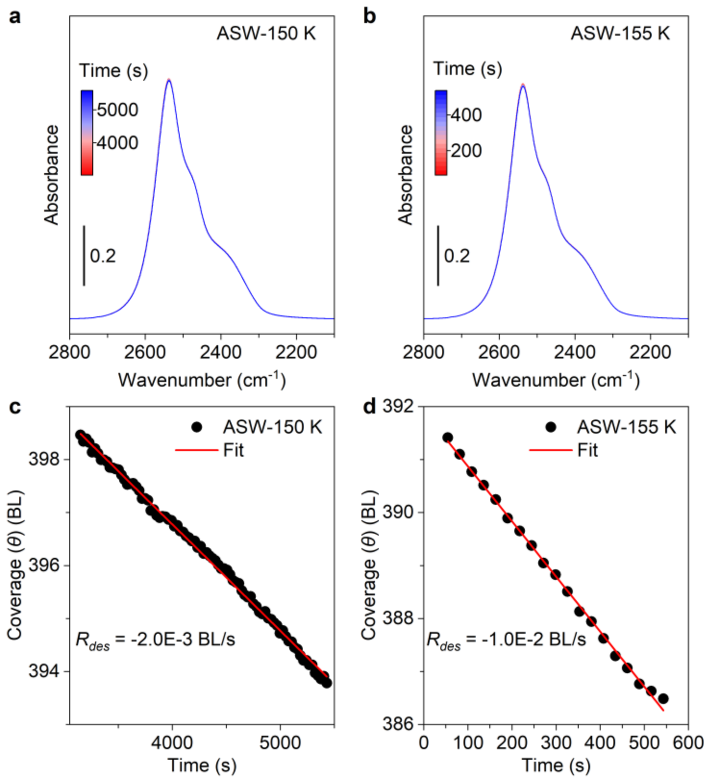

Supplementary Fig. 20. Time-resolved *p*-IRRAS spectra and desorption analysis of the OD stretching modes (ν_{OD}) in thick ASW films on Ru(0001). (**a, b**) Time-dependent *p*-polarized IRRAS spectra in the ν_{OD} region for ASW films recorded during isothermal annealing at 150 K and 155 K, respectively. (**c, d**) Corresponding time-dependent intensity profiles at 150 K and 155 K, obtained by integrating the absorbance in the ν_{OD} region from the spectra in (**a**) and (**b**).

At both temperatures, a continuous decrease in the overall ν_{OD} intensity is observed, indicating progressive desorption of D_2O molecules from the film surface. The black dots represent experimental data, while the solid red lines are fits used to extract desorption rates. As expected, the desorption rate increases with temperature, consistent with thermally activated desorption being more pronounced at 155 K as compared to 150 K.

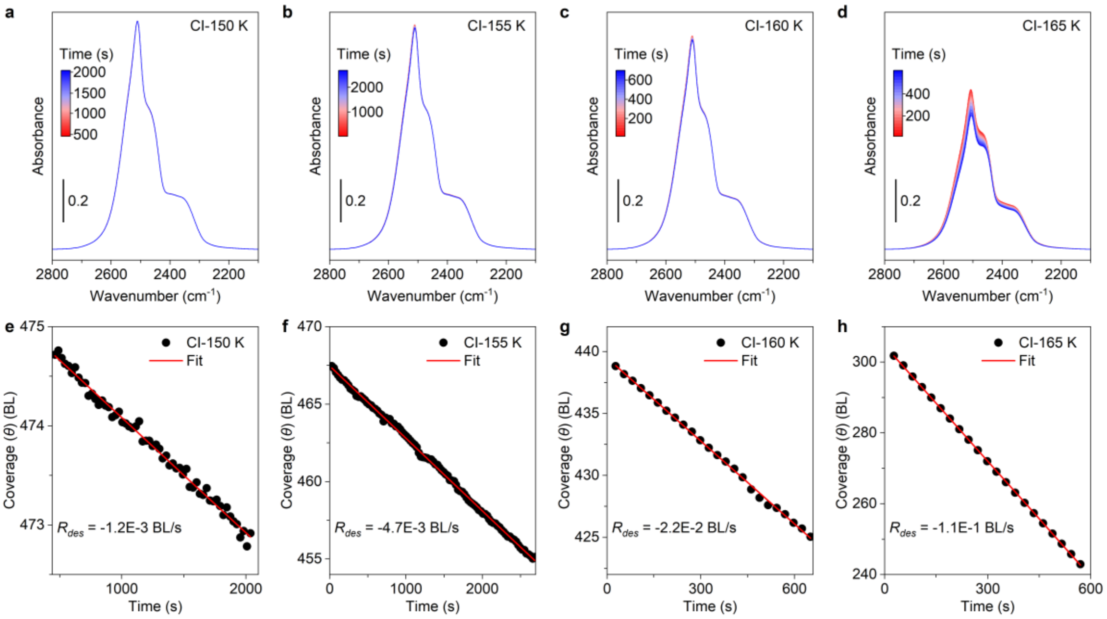

Supplementary Fig. 21. Time-resolved *p*-IRRAS spectra and desorption analysis of the OD stretching modes (ν_{OD}) in thick CI films on Ru(0001). (**a** – **d**) Time-dependent *p*-polarized IRRAS spectra in the ν_{OD} region for CI films recorded during isothermal annealing at 150 K, 155 K, 160 K and 165 K, respectively. (**e** – **h**) Corresponding time-dependent intensity profiles obtained by integrating the absorbance in the ν_{OD} region from the spectra in (**a** – **d**).

At all temperatures, a continuous decrease in the overall ν_{OD} intensity is observed, indicating progressive desorption of D_2O molecules from the film surface. The black dots represent experimental data, while the solid red lines are fits used to extract desorption rates.

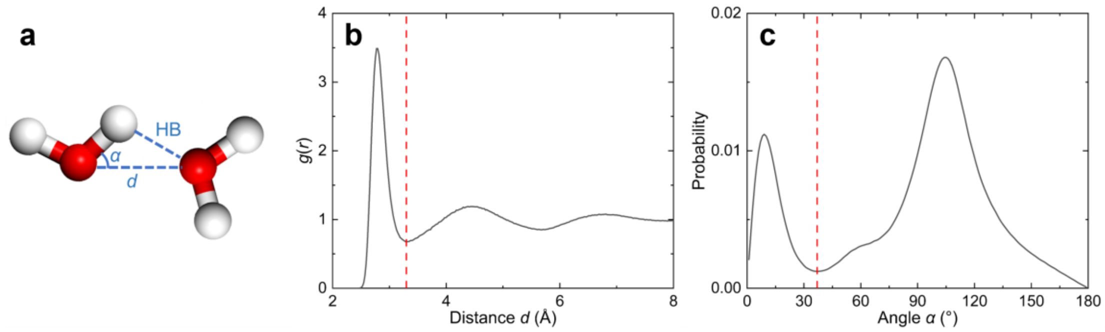

Supplementary Fig. 22. Definition of hydrogen bonds (HB). (**a**) Schematic illustration of a hydrogen bond. (**b**) and (**c**) Criteria used for HB identification in the MD simulations. (**b**) O-O radial distribution function. (**c**) H-O…O angle distribution function. The red dashed lines mark the cutoff values used for HB identification.

Supplementary Table 1. Experimentally derived values of the surface stabilization rate (r_{stbl}) and the associated surface stabilization energy barrier (E_{stbl}), and the stabilization intensity or the initial values (*K*) for the ice films prepared on Ru(0001).

		Temperature (*T*) (K)	Stabilization rate (r_{stbl}) (s^{-1})	Stabilization energy barrier (E_{stbl}) (kJ/mol)	Stabilization intensity or the initial (maximum) intensity (*K*) (a.u.)
**CI films**	CI	150 (145→150 K)	0.0008	79.9	0.0097
	CI	155 (150→155 K)	0	/	/
	CI	160 (155→160 K)	0.0011	84.9	0.0012
**ASW films**	ASW	150 (145→150 K)	0.0011	79.6	0.1000
	Crystallization	155 (150→155 K)	0.0071	79.8	0.0025
	CI	160 (155→160 K)	0.0011	84.9	0.0009

Supplementary Table 2. Relative intensities and FWHM of tetrahedral, distorted tetrahedral, and single-donor Raman subpeaks before and after annealing. Intensity ratios and FWHM reflect the structural evolution from amorphous to crystalline ice and the development of grain boundaries.

Relative intensities of Subpeaks	Tetrahedral	Distorted tetrahedral	Single donor
**Before annealing**	49.4%	37.8%	12.8%
**After annealing**	54.5%	15.2%	30.3%

FWHM of Subpeaks	Tetrahedral/cm^{-1}	Distorted tetrahedral/cm^{-1}	Single donor/cm^{-1}
**Before annealing**	64.3	182.8	103.8
**After annealing**	29.4	65.7	187.9

Code used in MD simulations

LAMMPS input file:

```
# initiallization
units               real
atom_style          full
boundary        p p p
# set temperature
variable        T equal 260
read_data           /data/relax.data
# force field parameter for Tip4p/ice
pair_style          lj/cut/tip4p/long 1 2 1 1 0.1577 10
kspace_style  pppm/tip4p 1.0e-4
pair_coeff          1 1 0.21084 3.1668
pair_coeff          1 2 0.0 0.0
pair_coeff          2 2 0.0 0.0
bond_style          harmonic
bond_coeff          1 1000.0 0.9572
angle_style         harmonic
angle_coeff         1 100.0 104.52
velocity            all create $T 1000
# keep water molecules rigid
fix             fixshake all shake 0.0001 20 0 b 1 a 1
# set timestep as 2 fs
timestep        2.0
# set NVT ensemble
fix             1 all nvt temp $T $T $(100*dt)
# output thermo file and dump file
thermo              1000
dump                1 all custom 10000 dump/$T.*.dump id type x y z
run             30000000
```

HB identification file implemented in Matlab:

```
clear;clc;
% HB criterion
R = 3.3;        % O-O distance
alpha = 37;    % H-O...O angle
% read LAMMPS output files
filepath='/data/HBdefinefiles/';
dumpall=dir(strcat(filepath,'260.*.dump'));
N = length(dumpall);
file_numbers = zeros(1, N);
% sort the output files by time
for i = 1:N
    file_numbers(i) = str2double(regexp(dumpall(i).name,'260\.(\d+)\.dump', 'tokens',
'once'));
end
[~, sort_indices] = sort(file_numbers);
sorted_files = dumpall(sort_indices);

for k = 1:N
    % read the data from the output files
    file=strcat(filepath,sorted_files(k).name);
    dump = fopen(file,'r');
    while feof(dump) == 0
        id = fgetl(dump);
        if (strncmpi(id,'ITEM: TIMESTEP',numel('ITEM: TIMESTEP')))
            timestep=str2num(fgetl(dump));
        else
            if (strncmpi(id,'ITEM: NUMBER OF ATOMS',numel('ITEM: NUMBER OF
ATOMS')))
                Natoms=str2num(fgetl(dump));
            else
                if (strncmpi(id,'ITEM: BOX BOUNDS',numel('ITEM: BOX
BOUNDS')))
                    x_bound(:)=str2num(fgetl(dump));
                    y_bound(:)=str2num(fgetl(dump));
                    z_bound(:)=str2num(fgetl(dump));
                else
                    if (strcmpi(id(1:11),'ITEM: ATOMS'))
                        for i=1:1:Natoms
                            atom_data(i,:)=str2num(fgetl(dump));
                        end
                    end
```

```
                    end
                end
            end
        end
        atom_data=sortrows(atom_data);
        xl=x_bound(2)-x_bound(1);
        yl=y_bound(2)-y_bound(1);
        zl=z_bound(2)-z_bound(1);

        % select data of oxygen and hydrogen atoms
        data_Oxygen=atom_data(atom_data(:,2)==1,1:5);
        data_Hydrogen=atom_data(atom_data(:,2)==2,1:5);
        n_O=size(data_Oxygen,1);
        n_H=size(data_Hydrogen,1);

        % determine all potential oxygen acceptor around each oxygen atom
        donor_A=zeros(10,5,n_O);
        for i=1:n_O
            nO=0;
            for j=1:n_O
                if (j~=i)
                    d_x = data_Oxygen(i,3) - data_Oxygen(j,3);
                    d_y = data_Oxygen(i,4) - data_Oxygen(j,4);
                    d_z = data_Oxygen(i,5) - data_Oxygen(j,5);
                    [d_x,d_y,d_z]=PBC(d_x,d_y,d_z,xl,yl,zl);
                    distance_0=sqrt(d_x^2+d_y^2+d_z^2);
                    if (distance_0<R)
                        nO=nO+1;
                        donor_A(nO,:,i)=data_Oxygen(j,:);
                    end
                end
            end
        end

        % calculate the angle between hydroxyl group and potential acceptor oxygen
        n_hbonds=0;
        for i=1:n_O
            Donor(i,1)=0;
            for ii=1:2
                dx_OH=data_Hydrogen(2*i+ii-2,3)-data_Oxygen(i,3);
                dy_OH=data_Hydrogen(2*i+ii-2,4)-data_Oxygen(i,4);
                dz_OH=data_Hydrogen(2*i+ii-2,5)-data_Oxygen(i,5);
                [dx_OH,dy_OH,dz_OH]=PBC(dx_OH,dy_OH,dz_OH,xl,yl,zl);
                O_H=[dx_OH, dy_OH, dz_OH];
```

```
        n_OO(i)=sum(donor_A(:,1,i)~=0);
        for jj=1:n_OO(i)
            dx_OO=donor_A(jj,3,i) - data_Oxygen(i,3);
            dy_OO=donor_A(jj,4,i) - data_Oxygen(i,4);
            dz_OO=donor_A(jj,5,i) - data_Oxygen(i,5);
            [dx_OO,dy_OO,dz_OO]=PBC(dx_OO,dy_OO,dz_OO,xl,yl,zl);
            O_O = [dx_OO, dy_OO, dz_OO];
            sita = (180/pi)*acos(dot(O_H,O_O)/(norm(O_H)*norm(O_O)));
            % determine whether the angle is less than 37°
            if (sita < alpha)
                n_hbonds= n_hbonds+ 1;
                Donor(i,1)=Donor(i,1)+1;
                dx_HO=donor_A(jj,3,i)-data_Hydrogen(2*i+ii-2,3);
                dy_HO=donor_A(jj,4,i)-data_Hydrogen(2*i+ii-2,4);
                dz_HO=donor_A(jj,5,i)-data_Hydrogen(2*i+ii-2,5);
                [dx_HO,dy_HO,dz_HO]=PBC(dx_HO,dy_HO,dz_HO,xl,yl,zl);
                length=sqrt(dx_HO^2+dy_HO^2+dz_HO^2);
                % output the HB information
                Hbonds_info(n_hbonds,1) = data_Oxygen(i,1);
                Hbonds_info(n_hbonds,2) = data_Hydrogen(2*i+ii-2,1);
                Hbonds_info(n_hbonds,3) = donor_A(jj,1,i);
                Hbonds_info(n_hbonds,4) = length;
                Hbonds_info(n_hbonds,5) = sita;
            end
        end
    end
end

% count the HB number formed of each hydroxyl group
for i=1:n_H
    HB(i,1)=0;
    for j=1:n_hbonds
        if (Hbonds_info(j,2)==data_Hydrogen(i,1))
            HB(i,1)=HB(i,1)+1;
        end
    end
end

% output matrix
D=zeros(n_H,2);
D(:,1)=data_Hydrogen(:,1);
D(:,2)=HB;
% output filepath
filename = ['/results/',sorted_files(k).name];
```

```
    [fid,message] = fopen(filename,"wt");
    for ii=1:n_H
        for jj=1:2
            fprintf(fid,'%f\t',D(ii,jj));
        end
        fprintf(fid,'\r\n');
    end
    fclose(fid);
    clearvars -except k R alpha filepath sorted_files N;
end

% periodic boundary function
function [dx,dy,dz] = PBC(dx,dy,dz,xl,yl,zl)
    dx=dx-xl*round(dx/xl);
    dy=dy-yl*round(dy/yl);
    dz=dz-zl*round(dz/zl);
end
```

Supplementary References

1 P. Yi *et al.* Intact water adsorption on Co(0001) at 100 K: transition from ordered bilayer to amorphous ice structures. *Phys. Chem. Chem. Phys.* **26**, 29724-29731 (2024).

2 Nojima, Y., Shioya, Y., Torii, H. & Yamaguchi, S. Hydrogen order at the surface of ice Ih revealed by vibrational spectroscopy. *Chem. Commun.* **56**, 4563-4566 (2020).

3 Gunkel, L. *et al.* Dynamic anti-correlations of water hydrogen bonds. *Nat. Commun.* **15**, 10453 (2024).

4 Sánchez, M. A. *et al.* Experimental and theoretical evidence for bilayer-by-bilayer surface melting of crystalline ice. *Proc. Natl Acad. Sci. USA* **114**, 227-232 (2017).

5 Pinna, A. *et al.* Release of Ceria Nanoparticles Grafted on Hybrid Organic–Inorganic Films for Biomedical Application. *ACS Appl. Mater. Interfaces* **4**, 3916-3922 (2012).

6 Carboni, D. *et al.* Enhanced Photocatalytic Activity in Low-Temperature Processed Titania Mesoporous Films. *J. Phys. Chem. C* **118**, 12000-12009 (2014).

7 Meyns, M., Primpke, S. & Gerdts, G. Library based identification and characterisation of polymers with nano-FTIR and IR-sSNOM imaging. *Anal. Methods* **11**, 5195-5202 (2019).

8 Sawatzki, M. P. Method and device for correcting a spectrum. *US Patent*, 7359815B7359812 (2008).

9 Wartewig, S. *IR and Raman Spectroscopy: Fundamental Processing*. (Wiley-VCH, 2003).

10 Peng, W. *et al.* Observation of Ice-Like Two-Dimensional Flakes on Self-Assembled Protein Monolayer without Nanoconfinement under Ambient Conditions. *Nano-Micro Lett.* **17**, 187 (2025).

11 Xu, K., Cao, P. & Heath, J. R. Graphene Visualizes the First Water Adlayers on Mica at Ambient Conditions. *Science* **329**, 1188-1191 (2010).

12 Lee, D. H. & Kim, K. Crystallization Mechanisms of Porous and Compact Amorphous Solid Water Films. *Cryst. Growth Des.* **25**, 978-985 (2025).

13 Harada, K., Sugimoto, T., Kato, F., Watanabe, K. & Matsumoto, Y. Thickness dependent homogeneous crystallization of ultrathin amorphous solid water films. *Phys. Chem. Chem. Phys.* **22**, 1963-1973 (2020).

14 Kondo, T., Kato, H. S., Bonn, M. & Kawai, M. Morphological change during crystallization of thin amorphous solid water films on Ru(0001). *J. Chem. Phys.* **126**, 181103 (2007).

15 Tonauer, C. M., Fidler, L.-R., Giebelmann, J., Yamashita, K. & Loerting, T. Nucleation and growth of crystalline ices from amorphous ices. *J. Chem. Phys.* **158**, 141001 (2023).

16 Smith, R. S., Matthiesen, J., Knox, J. & Kay, B. D. Crystallization Kinetics and Excess Free Energy of H2O and D2O Nanoscale Films of Amorphous Solid

Water. *J. Phys. Chem. A* **115**, 5908-5917 (2011).
17 Luo, S. *et al.* Molecular understanding of ion rejection in the freezing of aqueous solutions. *Phys. Chem. Chem. Phys.* **23**, 13292-13299 (2021).
18 Smit, W. J. *et al.* Excess Hydrogen Bond at the Ice-Vapor Interface around 200 K. *Phys. Rev. Lett.* **119**, 133003 (2017).
19 Backus, E. H. G., Grecea, M. L., Kleyn, A. W. & Bonn, M. Surface Crystallization of Amorphous Solid Water. *Phys. Rev. Lett.* **92**, 236101 (2004).
20 Yamauchi, T., Mine, K., Nakashima, Y., Izumi, A. & Namiki, A. Crystallization of D2O thin films on Ru(001) surfaces. *Appl. Surf. Sci.* **256**, 1124-1127 (2009).
21 Xu, Y. *et al.* Complete Wetting of Pt(111) by Nanoscale Liquid Water Films. *J. Phys. Chem. Lett.* **7**, 541-547 (2016).
22 Kringle, L., Thornley, W. A., Kay, B. D. & Kimmel, G. A. Reversible structural transformations in supercooled liquid water from 135 to 245 K. *Science* **369**, 1490-1492 (2020).
23 Kringle, L., Thornley, W. A., Kay, B. D. & Kimmel, G. A. Structural relaxation and crystallization in supercooled water from 170 to 260 K. *Proc. Natl Acad. Sci. USA* **118**, e2022884118 (2021).
24 Smith, R. S., Tylinski, M., Kimmel, G. A. & Kay, B. D. Crystallization kinetics of amorphous acetonitrile nanoscale films. *J. Chem. Phys.* **154** (2021).
25 Geissler, P. L. Temperature Dependence of Inhomogeneous Broadening: On the Meaning of Isosbestic Points. *J. Am. Chem. Soc.* **127**, 14930-14935 (2005).
26 Smith, J. D. *et al.* Unified description of temperature-dependent hydrogen-bond rearrangements in liquid water. *Proc. Natl Acad. Sci. USA* **102**, 14171-14174 (2005).
