# Supplementary material for: Formation of excess dangling OH bonds during crystallization of amorphous solid water
Source: Nat Commun. 2026 May 14;17:6420. doi: 10.1038/s41467-026-73221-x (PMC13376655; doi:10.1038/s41467-026-73221-x)
Supplement: Supplementary file 2 — Transparent Peer Review file [file 41467_2026_73221_MOESM2_ESM.pdf]

Peer Review File

Formation of excess dangling OH bonds during crystallization of amorphous solid water

Corresponding Author: Professor Jian-Qiang Zhong

This file contains all reviewer reports in order by version, followed by all author rebuttals in order by version.

Version 0:

Reviewer comments:

Reviewer #1

(Remarks to the Author)
This paper presents a very careful study of the crystallisation of amorphous solid water and the observation of an excess of dangling OH (dOH) bonds at the ice surface during that process. The work describes the use of isothermal measurements of the evolution of the RAIR spectrum of the ice with time. The observations are clearly described and their interpretation thorough and convincing.

The paper itself is well written and the figures are of a quality suitable for publication. My only minor concerns are

(1) "dOH" is already widely used in the ice spectroscopy literature to represent the dangling OH bond. Could the author replace their "DOB" with the more common descriptor usage throughout the manuscript.

(2) I would not hyphenated "in situ".

I am convinced that this paper is worthy of publication in Nature Communications.

Reviewer #2

(Remarks to the Author)
Cao, Zhong and co-workers, by combining experimental and computational approaches, present an interesting investigation on the formation of excess dangling OH bonds (DOB) during the crystallization process of amorphous solid water (ASW). Overall, the results and the evidences reported are convincing toward the observation of those DOB in the phase transition and the subject is certainly of broad interest. However, before reconsidering the paper for eventual publication several clarifications are in order, as I'll try to list in the following.

1. Since the abstract and until the final Discussion section, the Authors state that DOB serve as highly reactive sites in planetary and prebiotic environments. However, the realistic role of DOB and, in general, of species charaterizing the interfacial behavior in aqueous systems for catalysis is a highly debated topic [Angew. Chem. 64, e202418593 (2025); JACS 146, 31585 (2024); Chem. Sci. 13, 2574 (2022)] with highly contrasting explanations throughout the literature [JACS 147, 6299 (2025); JACS 146, 31585 (2024)]. Thus, I suggest to soften a bit the potential role of those species as catalytic agents and to mention recent literature highlighting controversies in the catalytic role of water interfaces.
2. Abstract: Rather than mentioning CI, the specific ice phase (Ice Ih) should be stated for clarity.
3. The Authors use deconvolution techniques to attribute vibrational features to donating (D) and accepting (A) hydrogen-bonded H_2O molecules. Although this might be -- to some extent -- justified, configurations like DDAA, DDA, DAA, and DA are oversimplifications and more complex interchanging H-bond phenomena occur. The limitations of deconvolution processes might be transparently stated and eventually discussed.
4. Are there evidences that the bands associated with 2500 and 2700 cm-1 are not coupled?
5. line 92: "...intensity is strongly temperature dependent...". This is only true for ASW and not for CI.
6. Crystallinity was extracted by decomposing spectra into ASW and CI components using fitting protocols. Please state limitations and eventual drawbacks of those fitting protocols. To the best of my knowledge, all fits have a non-negligible associated error.

7. In the same lines, when fitting x(t) via the Avrami eq. the Authors find n=4.1 and 3.2 from p- and s-IRRAS experiments. Which are the error bars?
8. Line 141: "experimental data exeeded the Avrami predictions,..." this should be better explained with reference to the respective data/plots.
9. The Authors employ different substrates (i.e., Ru(0001) and Cu(111)), which have different impacts on the temperature and timescales. Why? This should be clarified more extensively.
10. Is there any pH-dependent effect on the process, as it might be expected?
11. Line 238: DOB fractions are estimated (Fig. 4a-c). Please indicate the limitations and potential draback of those indirect estimates.
12. MD simulations. How free OH groups were identified/labeled rigorously?
13. MD simulations. More importantly, the rigorous identification of the ice-water instantaneous surface is everything but trivial! How the ice surface was identified to count the free OH groups?
14. Supplementary Fig. 3c does not report any data from MD simulation, as erronously stated in the Methods section.
15. If possible, please provide a template LAMMPS input file to ensure reproducibility.

Reviewer #3

(Remarks to the Author)
The manuscript "Formation of excess dangling OH bonds during crystallization of amorphous solid water" by authors Li et al describes an experimental and molecular dynamics study on the crystallization dynamics of deuterated amorphous water ice. The dangling OH (OD) bond, as measured in IRRAS spectra of ices grown on a cold substrate under UHV conditions, is used to probe crystallization, as is highly standard in such experiments. While the subject of the study would potentially be interesting for the physical chemistry of ices, a lack of coherence among results presented in the manuscript, lack of experimental detail provided, and an incoherence of the conclusions drawn compared to the extensive literature in the field mean that this manuscript would require major revisions, including reinterpretation of the dataset, before it could be reviewed for its scientific conclusions and potential novelty.

Decades of spectroscopic studies of water (H2O) ices deposited under HV and UHV conditions have shown that the transition from metastable amorphous ice to crystalline ice CI occurs rapidly and at temperatures below 150 K [see e.g. Hagen, Tielens & Greenberg Chem Phys 1981 56 367]. Additional information can be obtained from techniques including mass spectrometry. For example, the exact measured temperature of the 'volcano desorption' from mixed ices, measured via gas phase mass spectrometry and attributed to molecules ejected from the ice upon crystallization, varies as a function of the temperature ramp rate, but is generally observed around 140 K for slow heating [Smith et al PRL 1997 79 909, Collings et al MNRAS 2004 354 1133]. The energies of phase changes of deuterated water (D2O, used in this study) are very similar [Smith et al J Phys Chem A 2011 115 5908].

Unless some crucial detail is missing from the Methods section, such as the various experiments on ASW and CI being performed at orders of magnitude different pressures or deposition times, an ice deposited at 100 K and heated to 155 K will crystallize on a timescale significantly more rapid than the >1hour long experiments presented here. The authors have stated that under their experimental conditions, an ice deposited at 140 to 150 K has CI structure. Thus, the conclusion that ices remain amorphous upon timescales on the order of 30-60 minutes at 155 K, and therefore that their crystallization can be measured via IRRAS on that timescale, is not well supported by the data in the current manuscript.

It is recommended that authors revisit their interpretation in the light of studies such as those cited above and others such as [Dohnalek et al J Chem Phys 2000 112 5932]. Some issues that would need to be addressed include the following. The IR absorption band upon which the conclusions are drawn is of very low intensity. Experimental data, including full IR spectra, background correction procedures, and local correction around the DOB peak position should be exhaustively illustrated. The use of the term "time-resolved" with respect to the repeated measurement of IR spectra is undefined in the methods section. Details such as how many scans per spectrum are acquired and how long each spectrum takes to acquire are not provided. Full details of the method and the methodologies applied must be given. From the discussion and references provided, it is unclear how the authors draw the conclusion that ice surface restructuring at 155 K "may govern the reactivity of icy surfaces in astrophysical and planetary environments" as stated in the abstract.

Version 1:

Reviewer comments:

Reviewer #2

(Remarks to the Author)
The Authors, in my opinion, made a thorough review of the article and SI. Thus, the manuscript can now be accepted as it stands.

(Remarks on code availability)
The codes the Authors provided correctly run on Code Ocean.

Reviewer #4

(Remarks to the Author)
I was asked to review the authors responses to previous reviewer 3.

I am an IR spectroscopist with a particular focus on liquid water and its interactions with other materials. As such, I am familiar with the liquid water literature, but not as familiar with the extensive ice literature. It seems that most of the comments from all reviewers concerns particular disagreements of the present manuscript and this extensive literature.

Since I am not as familiar with this literature, I will refrain from commenting on this. Although I would say that it seems like the authors have done an extensive review of the literature. This includes the papers of Geissler on the isosbestic point of water, where I will agree with the authors that it is likely different for the present study.

However, although the authors now have provided further specific details on how the experiments were performed, the background subtraction method is not adequately described.

The added text now simply states that the “The rubberband algorithm was employed for background correction because it treats the baseline as a convex “envelope” that follows the lower edge of the spectrum, making it robust against broad overlapping absorption bands and largely insensitive to variations in peak shape. This procedure yields clean, baseline-flattened spectra and improves the reliability of peak integration and quantitative analysis. To assess the influence of the rubberband parameters, we additionally provide comparisons using a larger number of anchor points, which yield consistent trends and conclusions.”

However, this is not a description of what the method is or how it affects the data. I am not familiar with the method and there is no reference for it.

Is this an in-build function in the spectrometer or a home-built routine? In either way the method needs to be adequately described and validated.

Simply stating that “This procedure yields clean, baseline-flattened spectra and improves the reliability of peak integration and quantitative analysis.”, does not make it true.

However, from the plot in Figure T2(c), it does look like the method works well by eye, but this can be misleading. How does the method distinguish the smooth underlying absorption band of the hydrogen-bonded OH from the free-OH? What does the number of dots mean? How are these dots chosen and how does this affect the data?

Would you get a similar result with a simple two-point linear baseline subtraction? This would be more convincing. Is this the same as 2 dots in the rubberband method?

I am in general very skeptical about automated and/or complex baseline subtractions as these can significantly perturb the data.

Reviewer 3 also had comments on the “lack of coherence among the results” and “incoherence of the conclusions”. The authors ascribe this to specific observations, but I would think they were mean general in nature. When I read the short discussion starting line 312, I find the second paragraph starting line 326 rather clear but I cannot follow the first paragraph. The authors need reword this discussion to make it clearer.

(Remarks on code availability)

Version 2:

Reviewer comments:

Reviewer #4

(Remarks to the Author)
The authors have satisfactorily addressed my concerns, and I now recommend the manuscript for publication.

(Remarks on code availability)

Open Access This Peer Review File is licensed under a Creative Commons Attribution 4.0 International License, which permits use, sharing, adaptation, distribution and reproduction in any medium or format, as long as you give appropriate credit to the original author(s) and the source, provide a link to the Creative Commons license, and indicate if changes were made.
In cases where reviewers are anonymous, credit should be given to 'Anonymous Referee' and the source.
The images or other third party material in this Peer Review File are included in the article's Creative Commons license, unless indicated otherwise in a credit line to the material. If material is not included in the article's Creative Commons license and your intended use is not permitted by statutory regulation or exceeds the permitted use, you will need to obtain permission directly from the copyright holder.
To view a copy of this license, visit https://creativecommons.org/licenses/by/4.0/

Point-by-point responses to the reviewers' comments

Reviewer comments are shown in **blue**; author responses are shown in **black**; revisions to the manuscript are highlighted in **red**.

Reviewer #1 (Remarks to the Author):

This paper presents a very careful study of the crystallisation of amorphous solid water and the observation of an excess of dangling OH (dOH) bonds at the ice surface during that process. The work describes the use of isothermal measurements of the evolution of the RAIR spectrum of the ice with time. The observations are clearly described and their interpretation thorough and convincing.

Author Reply:

We thank the reviewer for the positive and encouraging comments.

The paper itself is well written and the figures are of a quality suitable for publication. My only minor concerns are

(1) "dOH" is already widely used in the ice spectroscopy literature to represent the dangling OH bond. Could the author replace their "DOB" with the more common descriptor usage throughout the manuscript.

Author Reply:

We thank the reviewer for this helpful suggestion. We have replaced "DOB" with the more commonly used term "dOH". To avoid confusion with the deuterated hydroxyl (OD), we now consistently use "dOH" for H_2O and "dOD" for D_2O throughout the manuscript.

(2) I would not hyphenated "in situ".

Author Reply:

We thank the reviewer for this kind suggestion. We have replaced "*in-situ*" with "*in situ*" throughout the manuscript.

I am convinced that this paper is worthy of publication in Nature Communications.

Author Reply:

We thank the reviewer again for the positive and encouraging comments.

Reviewer #2 (Remarks to the Author):

Cao, Zhong and co-workers, by combining experimental and computational approaches, present an interesting investigation on the formation of excess dangling OH bonds (DOB) during the crystallization process of amorphous solid water (ASW). Overall, the results and the evidences reported are convincing toward the observation of those DOB in the phase transition and the subject is certainly of broad interest. However, before reconsidering the paper for eventual publication several clarifications are in order, as I'll try to list in the following.

Author Reply:

We thank the reviewer for the positive and encouraging comments.

1. Since the abstract and until the final Discussion section, the Authors state that DOB serve as highly reactive sites in planetary and prebiotic environments. However, the realistic role of DOB and, in general, of species charaterizing the interfacial behavior in aqueous systems for catalysis is a highly debated topic [Angew. Chem. 64, e202418593 (2025); JACS 146, 31585 (2024); Chem. Sci. 13, 2574 (2022)] with highly contrasting explanations throughout the literature [JACS 147, 6299 (2025); JACS 146, 31585 (2024)]. Thus, I suggest to soften a bit the potential role of those species as catalytic agents and to mention recent literature highlighting controversies in the catalytic role of water interfaces.

Author Reply:

We appreciate the reviewer's thoughtful suggestion. We have revised the corresponding sections to moderate the discussion regarding the catalytic role of dOH and to acknowledge that this topic remains highly controversial in the literature.

Revision in the manuscript:

Page 1, line 18-19:

Dangling OH bonds (dOH) on ice surfaces are thought to play a central role in surface reactions relevant to planetary, interstellar, and prebiotic environments, ……

Page 1, line 27-28:

……and uncover a surface-restructuring pathway that may influence the reactivity of icy surfaces.

Page 1, line 37-39:

……and prebiotic molecular evolution [3]. In liquid and solid water systems, however, the catalytic roles and mechanistic significance of such dOH species remain subjects of continued debate [4]. Despite extensive studies, the molecular-scale insight into the structure and dynamics of ice and liquid water surfaces still lags behind that of the bulk [5,6], owing to the structural fragility and extreme sensitivity of surface hydrogen-bonding networks (HBN) to electric fields [7,8], and to fluctuations in temperature and pressure [9].

2. Abstract: Rather than mentioning CI, the specific ice phase (Ice Ih) should be stated for clarity.

Author Reply:

We thank the reviewer for this valuable suggestion. We note that whether amorphous solid water (ASW) crystallizes into purely hexagonal ice (Ice I*h*) remains an open question. Several studies have shown that ASW crystallizes into stacking-disordered ice (Isd), which is characterized by a mixture of cubic and hexagonal stacking sequences (***Proc. Natl. Acad. Sci.,*** *109, 1041 – 1045* (***2012***)*;* ***Nature****, 551, 218 – 222* (***2017***)*;* ***J. Phys. Chem. Lett.,*** *13, 8601 – 8606* (***2022***)). To ensure accuracy and consistency with current understanding, we therefore describe the crystallized phase using the more general term "crystalline ice (ice I)" rather than explicitly assigning it to pure ice I*h*. We have revised the description and specified the detailed phase of the crystalline ice accordingly.

Revision in the manuscript:

Page 1, line 23:

……we tracked the evolution of dOH during the isothermal crystallization of amorphous solid water (ASW) into crystalline ice (CI), i.e., ice I.

3. The Authors use deconvolution techniques to attribute vibrational features to donating (D) and accepting (A) hydrogen-bonded H2O molecules. Although this might be -- to some extent -- justified, configurations like DDAA, DDA, DAA, and DA are oversimplifications and more complex interchanging H-bond phenomena occur. The limitations of deconvolution processes might be transparently stated and eventually discussed.

Author Reply:

We thank the reviewer for this helpful suggestion, and we completely agree that configurations such as DDAA, DDA, DAA, and DA are indeed oversimplifications. In reality, much more complex and dynamic hydrogen-bonding phenomena occur in both ice and water structures (***Nature****, 365, 327 – 329* (***1993***); ***J. Phys. Chem. Lett.****, 8, 3656 – 3660* (***2017***)*;* ***Nature****, 596, 531 – 535* (***2021***)).

Numerous IRRAS studies have been performed on various ice films, including low-density amorphous (LDA) ice, high-density amorphous (HDA) ice, hyperquenched glassy water (HGW), and crystalline ice (CI, ice I*h*). However, detailed interpretation of the broad OD stretching bands, ν_{OD}, remains challenging. Even for well-defined ice bilayer (***J. Am. Chem. Soc.****, 131, 12838 – 12844* (***2009***)), deconvolution of the ν_{OD} band is difficult because the OD stretching strength is highly sensitive to local hydrogen-bonding environments. For instance, even within DDAA-type configurations, both the peak position and bandwidth may vary depending on subtle structural and electronic differences (***Phys. Chem. Chem. Phys.*** *19, 8288 – 8299* (***2017***)).

Considerable efforts have been made to clarify the components of the broad ν_{OD} feature observed in IRRAS spectra (***Chem. Phys. Lett.,*** *389, 92 – 95* (***2004***)*;* ***J. Phys. Chem. A****, 111, 10119 – 10122* (***2007***)*;* ***J. Chem. Phys.****, 148, 244107* (***2018***)). For

example, Feibelman proposed that the stretching frequency primarily reflects the O-O distance distribution within the hydrogen-bonded network of a water wetting layer on metal surfaces. Nevertheless, the precise origin of the experimentally observed peak shapes remains an open question.

Currently, the widely accepted assignment of the ν_{OD} band for thick ice films (D_2O) is that amorphous solid water (ASW) and crystalline ice (CI) exhibit characteristic peaks at approximately ~2535 cm^{-1} and ~2500 cm^{-1}, respectively. These assignments should be regarded as descriptors of the dominant vibrational environments within each phase, which give rise to the most recognizable absorption features. The dangling OD features appear at higher frequencies: for instance, bands around ~2750 cm^{-1} and ~2726 cm^{-1} are generally attributed to two- and three-coordinated surface water molecules, respectively (***J. Phys. Chem. B**, 109, 5816 – 5823 (**2005**)*).

In our work, we did not attempt a detailed deconvolution of the broad ν_{OD} band for thick ice films. Instead, we followed the widely accepted approach that different ice phases exhibit characteristic peak positions and spectral shapes. Two complementary methods were employed to analyze the crystallinity of the ice films.

First, an isosbestic point – where all spectra intersect – was observed during the isothermal crystallization process, indicating that the overall absorbance can be described as a linear combination of two phases: ASW and CI. The crystallinity was therefore extracted by decomposing each spectrum into ASW and CI components, as shown in **Fig. 2**. This method is widely adopted for analyzing the crystallinity of ice films (***Phys. Rev. Lett.** 92, 236101 (**2004**); **J. Phys. Chem. Lett.**, 7, 541 – 547 (**2016**); **Science** 369, 1490–1492 (**2020**)*).

Second, we monitored the temporal evolution of the IRRAS absorbance intensity at ~2465 cm^{-1} (~3300 cm^{-1} for H_2O), which is also widely used for analyzing the crystallinity of ice films (***J. Phys. Chem. A** 115, 5908–5917 (**2011**); **J. Phys. Chem. C** 122, 24164–24170 (**2018**)*). The corresponding data are presented in **Supplementary Fig. 4.**

In response to the reviewer's suggestion, we have expanded the discussion slightly in the revised manuscript to further clarify the complexity of the ν_{OD} band and the rationale behind our analytical approach.

Revision in the manuscript:

Page 2, line 83-88:

……water molecules primarily adopt configurations such as DDAA, DDA, DAA, and DA, depending on their roles as hydrogen-bond donor (D) and acceptor (A). Considerable efforts have been devoted to identifying the subcomponents contributing to the broad ν_{OD} stretching band observed in IRRAS spectra [34,35]; however, full deconvolution remains challenging, as proton disorder and variations in O-O distances or O-D bond lengths lead to substantial spectral broadening [34,35]. Nevertheless, upon crystallization, the dominant ν_{OD} band redshifts from ~2535 cm^{-1} in ASW to ~2504 cm^{-1} in CI, consistent with stronger, more ordered hydrogen bonding [36]. These most intense peaks are therefore used as characteristic signatures of ASW and CI, respectively [36].

Page 4, line 122-123:

As crystallization progressed, the ν_{OD} bands gradually red-shifted, and all spectra intersected at an isosbestic point. This feature indicates that the overall absorbance can be approximated as a linear combination of two phases, ASW and CI, under the assumption of a two-state (ASW/CI) mixture model (**Supplementary Fig. 2**). Such analysis has been widely and successfully applied to characterize structural transitions in various amorphous and crystalline molecular films [24,37,40,44]. Using this approach, the crystallinity was extracted by……

4. Are there evidences that the bands associated with 2500 and 2700 cm-1 are not coupled?

Author Reply:

We thank the reviewer for this insightful question. Both our experimental observations and previous studies indicate that the bands around 2500 cm^{-1} (bulk ν_{OD} stretch) and 2700 cm^{-1} (surface $\nu_{free\text{-}OD}$ stretch) in amorphous and crystalline ice are, to a good approximation, spectroscopically decoupled, i.e., any vibrational coupling between them is weak (***J. Chem. Phys.*** *102, 8328 – 8341 (**1995**); **J. Chem. Phys.** 132, 204505 (**2010**); **J. Chem. Phys.** 140, 134503 (**2014**)*). The structural evolution of the bulk hydrogen-bonding network can slightly modify the local environment of the surface $\nu_{free\text{-}OD}$, but the reverse effect, changes in $\nu_{free\text{-}OD}$ perturbing the bulk ν_{OD} band, is negligible.

(1) In IRRAS spectra, the broad ν_{OD} region (2100 – 2800 cm^{-1}) arises predominantly from a superposition of the fundamental ν_1 (symmetric stretch) and ν_3 (asymmetric stretch), with a minor contribution from the overtone $2\nu_2$ (bend). The ν_1 and ν_3 modes are strongly coupled because the hydrogen-bond network converts molecular stretching vibrations into collective, delocalized normal modes. Hydrogen bonding reduces the ν_1-ν_3 energy separation, enhances transition dipole-dipole interactions, and leads to substantial mode mixing, so that the IR-active OD stretching band in ice is best viewed as a coupled vibrational manifold rather than separate ν_1 and ν_3 peaks. In contrast, the $\nu_{free\text{-}OD}$ feature corresponds to a localized, non-fully-hydrogen-bonded OD oscillator at the ice-vacuum interface, which is only weakly mixed with the bulk-like modes.

(2) In ASW, the bulk ν_{OD} band remains nearly unchanged over a broad temperature range in the absence of crystallization, whereas the surface $\nu_{free\text{-}OD}$ feature varies significantly with temperature (**Fig. 1a**). This clear difference in temperature dependence shows that changes in $\nu_{free\text{-}OD}$ do not influence the bulk ν_{OD} band; instead, ν_{OD} primarily reflects the bulk hydrogen-bond network, while $\nu_{free\text{-}OD}$ reflects the surface hydrogen-bond topology.

(3) During isothermal crystallization, the main peak of the bulk ν_{OD} band red-shifts by ~30 cm^{-1} and narrows as hydrogen bonding becomes stronger and more ordered (**Fig. 2a**). In contrast, the $\nu_{free\text{-}OD}$ feature undergoes only a small shift (~1 – 2 cm^{-1}) and moderate narrowing, associated with the reorganization of the surface hydrogen-bond environment (**Supplementary Fig. 15**). These correlated but clearly different spectral

changes indicate that bulk reorganization can subtly influence the surface environment, yet the two bands evolve largely independently, consistent with weak coupling between bulk and surface modes.

(4) Early studies of ice surfaces have shown that the $\nu_{\text{free-OH/OD}}$ modes are spatially localized at the outermost molecular layers and exhibit only weak vibrational coupling (on the order of a few wavenumbers) to the bulk hydrogen-bonded network (***J. Chem. Phys.**, 94, 812 (**1991**)*; ***J. Chem. Phys.**, 94, 4091 (**1991**); **J. Chem. Phys.**, 95, 1378 (**1991**)*). In particular, simulations of ice surfaces reproduce a distinct, narrow $\nu_{\text{free-OH/OD}}$ band that can be assigned to surface-localized oscillators, in contrast to the broad, strongly coupled bulk band (***J. Chem. Phys.** 102, 8328 – 8341 (**1995**); **J. Chem. Phys.** 145, 044703 (**2016**)*). These results support the interpretation that the ν_{OD} and $\nu_{\text{free-OD}}$ bands correspond to physically distinct vibrational populations, with only minor dynamical coupling between them.

Taken together, these arguments justify our treatment of the 2500 cm^{-1} (bulk ν_{OD}) and 2700 cm^{-1} (surface $\nu_{\text{free-OD}}$) bands as effectively uncoupled for the purposes of our analysis, while fully acknowledging that, at a microscopic level, a small residual intermolecular coupling between surface and subsurface oscillators may still exist.

5. line 92: "...intensity is strongly temperature dependent...". This is only true for ASW and not for CI.

Author Reply:

We thank the reviewer for pointing this out. We agree that the statement "……$\nu_{\text{free-OD}}$ intensity is strongly temperature dependent" applies primarily to ASW, whereas in CI films the temperature dependence is much weaker and manifests only as a shallow minimum near 155 K. We have revised the text accordingly.

Revision in the manuscript:

Page 3, line 91-100:

As shown in the enlarged views in **Fig. 1a** and **1c**, the temperature dependence of the $\nu_{\text{free-OD}}$ intensity is pronounced for ASW but much weaker for CI. In ASW films (**Fig. 1b**), spectra collected after prolonged annealing (~10 min, solid diamonds) differ noticeably from those obtained after flash annealing followed by rapid cooling (< 1 min, hollow diamonds), with both protocols controlled to avoid bulk crystallization. The shaded region between the two datasets defines the range of $\nu_{\text{free-OD}}$ intensities accessible under different thermal histories. Overall, $\nu_{\text{free-OD}}$ decreases with increasing temperature up to ~160 K, consistent with progressive reorganization of the surface HBN. In contrast, CI films (**Fig. 1d**) exhibit only a weak temperature dependence: the $\nu_{\text{free-OD}}$ intensity displays a shallow minimum near 155 K, a behavior also reproduced by our molecular dynamics (MD) simulations (inset, **Fig. 1d**).

6. Crystallinity was extracted by decomposing spectra into ASW and CI components using fitting protocols. Please state limitations and eventual drawbacks of those fitting

protocols. To the best of my knowledge, all fits have a non-negligible associated error.

Author Reply:

We thank the reviewer for raising this important point. We agree that the decomposition of the ν_{OD} spectra into ASW and CI components inevitably introduces uncertainties. Nevertheless, this fitting protocol has been widely and successfully applied to analyze structural transitions in various amorphous and crystalline molecular films, including water (e.g., ***Phys. Rev. Lett.*** *92, 236101* ***(2004)***; ***Appl. Surf. Sci.*** *256, 1124 – 1127* ***(2009)***; ***J. Phys. Chem. Lett.*** *7, 541 – 547* ***(2016)***; ***Science*** *369, 1490 – 1492* ***(2020)***; ***Proc. Natl. Acad. Sci.*** *118, e2022884118* ***(2021)***) and other cryogenic films such as amorphous acetonitrile (***J. Chem. Phys.*** *154, 144703* ***(2021)***).

The primary limitation of this approach stems from the assumption of a two-state (ASW/CI) mixture model, in which each intermediate spectrum is represented as a linear combination of ASW and CI reference spectra. This assumption inherently neglects the existence of possible "intermediate" structures that arise during nucleation and crystal growth. According to classical crystallization theory, the region surrounding a growing crystalline nucleus cannot be strictly categorized as either ASW or CI. As a result, such transition structures are not captured by the two-state model, leading to increased fitting residuals, especially during the periods with the highest crystallization rates. As shown in **Supplementary Fig. 2**, the fitting residual can reach ~2.3% for *p*-IRRAS and ~7.0% for *s*-IRRAS during the periods of rapid crystallization, reflecting the influence of such intermediate structures.

We also note that Geissler and co-workers demonstrated that the presence of isosbestic points does not necessarily imply a two-state transformation, as similar features may emerge in systems dominated by temperature-dependent continuous spectral broadening (***J. Am. Chem. Soc.*** *127, 14930 – 14935* ***(2005)***; ***Proc. Natl. Acad. Sci.*** *102, 14171 – 14174* ***(2005)***). However, these theoretical considerations were developed for liquid water over broad temperature variations (275 – 355 K). In the present study, crystallization is performed isothermally, where temperature-induced inhomogeneous broadening is negligible. Under these conditions, the two-state approximation remains appropriate and has been extensively validated in previous analyses of ASW crystallization.

We validated the crystallinity using two independent approaches: (i) full spectral decomposition into ASW and CI components (**Fig. 2**), and (ii) monitoring the absorbance change at 2465 cm^{-1}, a widely used crystallinity indicator (**Supplementary Fig. 4**). Both methods produce consistent crystallization curves within the expected experimental uncertainties.

In summary, we thank the reviewer again for raising this important point. As the reviewer correctly noted, the fitting procedure introduces small but unavoidable errors and uncertainties. We now explicitly discuss these limitations in both the main manuscript and the Supplementary Information.

Revision in the manuscript:

Page 4, line 122-123:

As crystallization progressed, the ν_{OD} bands gradually red-shifted, and all spectra

intersected at an isosbestic point. This feature indicates that the overall absorbance can be approximated as a linear combination of two phases, ASW and CI, under the assumption of a two-state (ASW/CI) mixture model (**Supplementary Fig. 2**). Such analysis has been widely and successfully applied to characterize structural transitions in various amorphous and crystalline molecular films [24,37,40,44].

Revision in the Supplementary Information:

We have added one figure (**Supplementary Fig. 2**) and more discussions in the revised **Supplementary Information.**

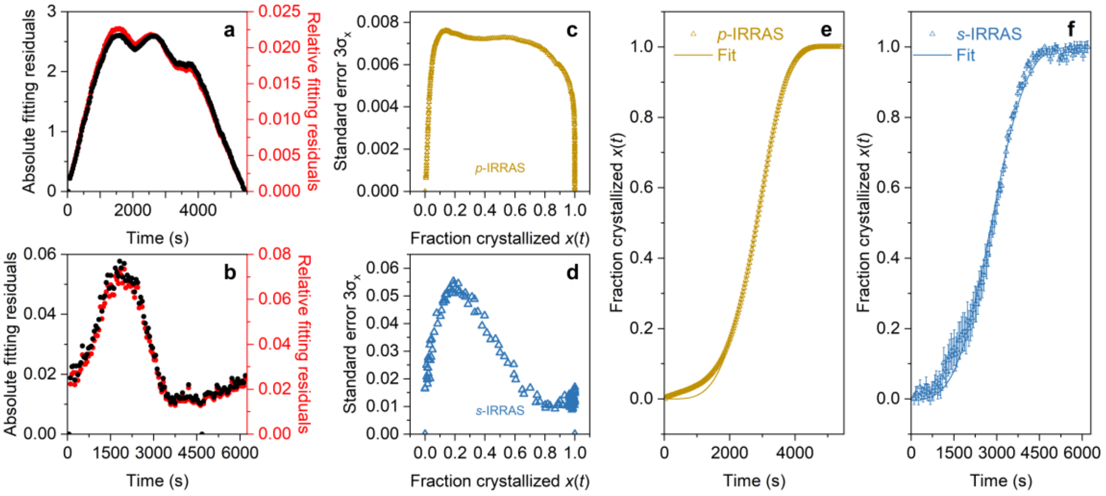

Supplementary Fig. 2. Estimation of the uncertainties in $x(t)$ and n shown in Fig. 2 of the main text. (**a, b**) Absolute fitting residuals and relative fitting residuals of $x(t)$ for the spectra shown in **Fig. 2a** and **2b**, respectively. (**c, d**) Standard errors of $x(t)$ obtained from the spectral fittings of each intermediate spectrum using the two-state (ASW/CI) mixture model. (**e, f**) Time-dependent fraction crystallized $x(t)$ with error bars determined from the corresponding standard errors, together with the Avrami fits used to extract the Avrami exponent n.

Each intermediate spectrum was modeled as a linear combination of the ASW and CI reference spectra, i.e., $(1 - x(t))$ASW + $(x(t))$CI, where the first and last spectra in the crystallization sequence were taken as the ASW and CI references, respectively. The first spectrum corresponds to the fully amorphous state ($x = 0$), while the last spectrum corresponds to the fully crystalline state ($x = 1$). For each intermediate spectrum, the crystallinity $x(t)$ was determined by least-squares regression. The uncertainty of $x(t)$ (reported as a 3σ standard error) was calculated from the variance of the fitting residuals. It is important to note that the signal-to-noise ratio of the IRRAS measurements is sufficiently high such that the additional uncertainty associated with numerical integration of the ν_{OD} peak area is negligible. The uncertainty of the Avrami exponent n was determined by fitting the upper and lower bounds of the crystallinity, $x(t) \pm 3\sigma(t)$.

The decomposition of the ν_{OD} spectra into ASW and CI components inevitably introduces uncertainties. Nevertheless, this fitting protocol has been widely and successfully applied to analyze structural transitions in various amorphous and

crystalline molecular films, including water [14-18] and other cryogenic films such as amorphous acetonitrile [19].

The primary limitation of this approach stems from the assumption of a two-state (ASW/CI) mixture model, in which each intermediate spectrum is represented as a linear combination of ASW and CI reference spectra. This assumption inherently neglects the possible existence of intermediate structures that may form during nucleation and crystal growth. According to classical crystallization theory, the interfacial region surrounding a growing crystalline nucleus cannot be strictly categorized as either ASW or CI. Consequently, such transition structures are not captured by the two-state model, leading to increased fitting residuals, particularly during the periods of rapid crystallization. As shown in **Supplementary Fig. 2a** and **2b**, the relative fitting residuals can reach ~2.3% for *p*-IRRAS and ~7.0% for *s*-IRRAS during these stages, reflecting the influence of such intermediate structures.

We also note that Geissler and co-workers demonstrated that the presence of isosbestic points does not necessarily imply a two-state transformation, as similar features may emerge in systems dominated by temperature-dependent continuous spectral broadening [20,21]. However, these theoretical considerations were developed for liquid water over a broad temperature range (275 – 355 K). In the present study, crystallization is performed isothermally, where temperature-induced inhomogeneous broadening is negligible. Under these conditions, the two-state approximation remains appropriate and has been extensively validated in previous analyses of ASW crystallization.

7. In the same lines, when fitting x(t) via the Avrami eq. the Authors find n=4.1 and 3.2 from p- and s-IRRAS experiments. Which are the error bars?

Author Reply:

We thank the reviewer for this important question. In the revised **Fig. 2c**, we have included the uncertainties for n, and we have added a new supplementary figure (**Supplementary Fig. 2**) to illustrate the fitting residuals, the standard errors, and the error bars.

As discussed in our response to **Comment 6**, the dominant source of uncertainty in the extracted crystallinity $x(t)$ arises from the assumptions inherent in the two-state (ASW/CI) mixture model used for spectral decomposition. Because this approach does not explicitly account for intermediate or transition structures at the ASW/CI interface during nucleation and growth, the fitting residuals increase during the period of highest crystallization rate. The uncertainty in $x(t)$ was estimated as three times the standard deviation (3σ) of the fitted values. It is important to note that the signal-to-noise ratio of our IRRAS measurements is sufficiently high, so the additional uncertainty associated with numerical integration of the ν_{OD} peak area is negligible.

Thus, to quantify the overall uncertainty in the Avrami exponent n, we directly fitted the upper and lower bounds of the crystallinity fraction, $x(t) \pm 3\sigma(t)$. This analysis yields Avrami exponents of $n = 4.1 \pm 0.1$ (*p*-IRRAS) and $n = 3.4 \pm 0.5$ (*s*-IRRAS).

Because the uncertainty in $x(t)$ is small, particularly for the *p*-IRRAS data, the corresponding error bars are nearly indistinguishable at the scale of **Fig. 2c** in the main manuscript. Therefore, **Fig. 2c** remains unchanged. For completeness, we now provide enlarged versions of **Fig. 2c** with explicit error bars as **Supplementary Figs. 2e and 2f** in the revised Supplementary Information.

Revision in the manuscript:

Page 4: Fig. 2c has been modified to include uncertainties.

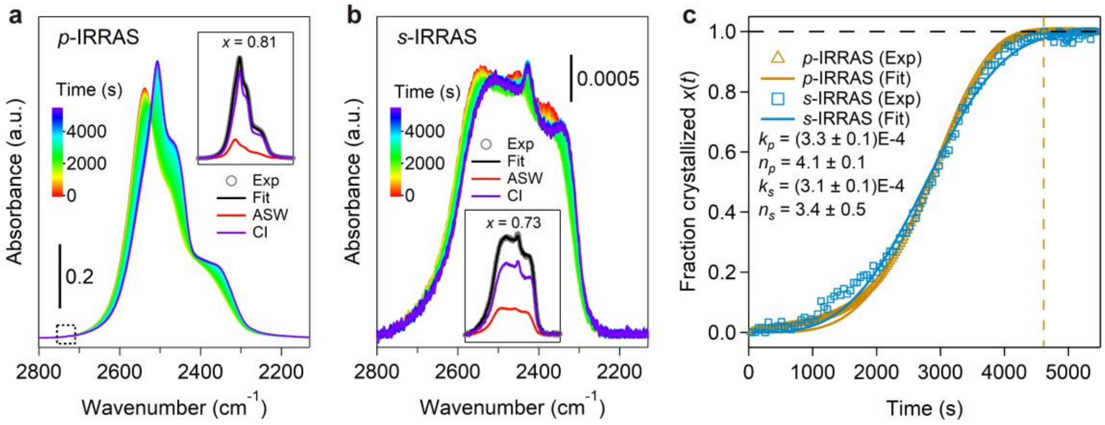

Page 4: line 137-138:

Fitting $x(t)$ yielded Avrami exponents of $n = 4.1 \pm 0.1$ (*p*-IRRAS) and $n = 3.4 \pm 0.5$ (*s*-IRRAS), indicating……

Revision in the Supplementary Information:

We have added one figure (**Supplementary Fig. 2**) and more discussions in the revised **Supplementary Information.**

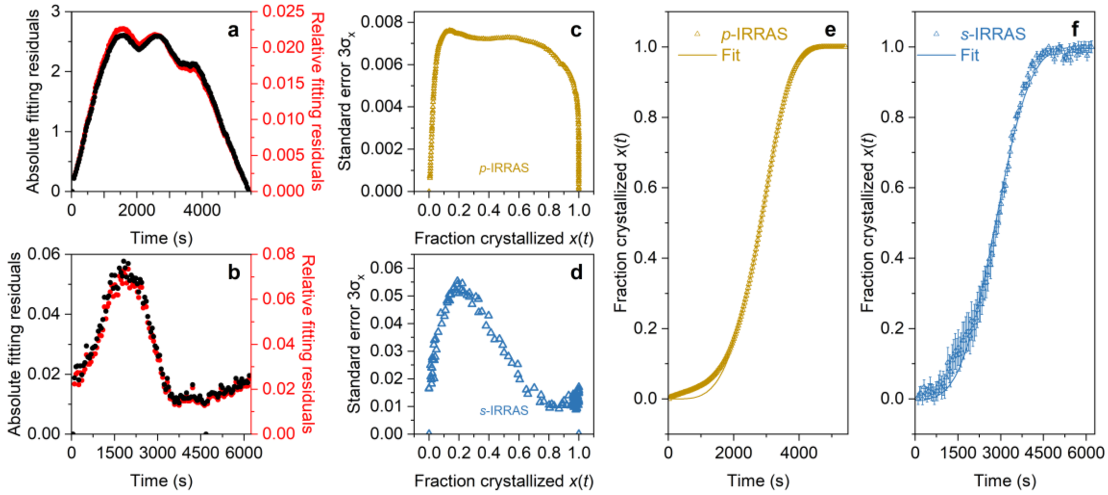

Supplementary Fig. 2. Estimation of the uncertainties in $x(t)$ and n shown in Fig. 2 of the main text. (**a, b**) Absolute fitting residuals and relative fitting residuals of $x(t)$ for the spectra shown in **Fig. 2a** and **2b**, respectively. (**c, d**) Standard errors of $x(t)$ obtained from the spectral fittings of each intermediate spectrum using the two-state (ASW/CI) mixture model. (**e, f**) Time-dependent fraction crystallized $x(t)$ with error bars determined from the corresponding standard errors, together with the Avrami

fits used to extract the Avrami exponent n.

Each intermediate spectrum was modeled as a linear combination of the ASW and CI reference spectra, i.e., $(1 - x(t))$ASW + $(x(t))$CI, where the first and last spectra in the crystallization sequence were taken as the ASW and CI references, respectively. The first spectrum corresponds to the fully amorphous state ($x = 0$), while the last spectrum corresponds to the fully crystalline state ($x = 1$). For each intermediate spectrum, the crystallinity $x(t)$ was determined by least-squares regression. The uncertainty of $x(t)$ (reported as a 3σ standard error) was calculated from the variance of the fitting residuals. It is important to note that the signal-to-noise ratio of the IRRAS measurements is sufficiently high such that the additional uncertainty associated with numerical integration of the ν_{OD} peak area is negligible. The uncertainty of the Avrami exponent n was determined by fitting the upper and lower bounds of the crystallinity, $x(t) \pm 3\sigma(t)$.

The decomposition of the ν_{OD} spectra into ASW and CI components inevitably introduces uncertainties. Nevertheless, this fitting protocol has been widely and successfully applied to analyze structural transitions in various amorphous and crystalline molecular films, including water [14-18] and other cryogenic films such as amorphous acetonitrile [19].

The primary limitation of this approach stems from the assumption of a two-state (ASW/CI) mixture model, in which each intermediate spectrum is represented as a linear combination of ASW and CI reference spectra. This assumption inherently neglects the possible existence of intermediate structures that may form during nucleation and crystal growth. According to classical crystallization theory, the interfacial region surrounding a growing crystalline nucleus cannot be strictly categorized as either ASW or CI. Consequently, such transition structures are not captured by the two-state model, leading to increased fitting residuals, particularly during the periods of rapid crystallization. As shown in **Supplementary Fig. 2a** and **2b**, the relative fitting residuals can reach ~2.3% for *p*-IRRAS and ~7.0% for *s*-IRRAS during these stages, reflecting the influence of such intermediate structures.

We also note that Geissler and co-workers demonstrated that the presence of isosbestic points does not necessarily imply a two-state transformation, as similar features may emerge in systems dominated by temperature-dependent continuous spectral broadening [20,21]. However, these theoretical considerations were developed for liquid water over a broad temperature range (275 – 355 K). In the present study, crystallization is performed isothermally, where temperature-induced inhomogeneous broadening is negligible. Under these conditions, the two-state approximation remains appropriate and has been extensively validated in previous analyses of ASW crystallization.

8. Line 141: "experimental data exeeded the Avrami predictions,..." this should be better explained with reference to the respective data/plots.

Author Reply:

We thank the reviewer for this helpful suggestion. In the revised manuscript, we have clarified the description and expanded the discussion.

In **Fig. 2c**, the experimentally extracted crystallinity $x(t)$ displays a slight overshoot relative to the Avrami prediction during the earliest stage of crystallization. This phenomenon, where the experimental crystallinity temporarily exceeds the Avrami fit, has been widely reported across multiple experimental modalities, including transmission electron microscopy (TEM) (***Nat. Commun.*** *15, 908 (**2024**)*), isothermal desorption measurements (***Cryst. Growth Des.*** *25, 978 – 985 (**2025**)*), temperature-programmed desorption (TPD) and IRRAS (***J. Phys. Chem. A*** *115, 5908 – 5917 (**2011**)*).

Conversely, other IRRAS studies have documented the opposite behavior, where the crystallinity initially falls below the Avrami prediction. Such deviations depend sensitively on the specific crystallization scenario, for example, the crystallization occurring at interfaces (***Phys. Rev. Lett.*** *92, 236101 (**2004**)*), studies employing thin ASW films (***Phys. Chem. Chem. Phys.*** *22, 1963 – 1973 (**2020**)*), crystallization performed at temperatures with extremely slow kinetics (***J. Phys. Chem. A*** *115, 5908 – 5917 (**2011**)*), or differences in film morphology such as porous *vs.* compact ASW (***Cryst. Growth Des.*** *25, 978 – 985 (**2025**)*).

Furthermore, both the sign and magnitude of the deviation are known to depend on the Avrami exponent n (***J. Chem. Phys.*** *126, 181103 (**2007**); **Metall. Mater. Trans. B*** *49, 3616 – 3680 (**2018**); **J. Chem. Phys.*** *158, 141001 (**2023**)*).

Based on our ongoing studies of crystallization-front dynamics, we attribute the initial excess of the experimental $x(t)$ relative to the Avrami model in **Fig. 2c** to a pre-ordering regime in which the increase in the number of nuclei (domains) initially dominates over their volumetric growth. This regime is highly sensitive to crystallization temperature and to the microstructure of the ASW films (e.g., thickness, porosity). Because the classical Avrami formalism does not explicitly account for such pre-ordering, deviations can manifest either as an overshoot or undershoot depending on the system.

We have now revised the manuscript to highlight that these deviations represent a well-documented phenomenon in crystallization kinetics and constitute an area that merits further investigation.

Revision in the manuscript:

Page 4, line 141-143:

During early stages of crystallization, the experimental data deviate from the Avrami prediction, a phenomenon widely reported across multiple characterization methods [36,43,52]. The magnitude and even the direction of these deviations depend sensitively on crystallization temperature and on ASW film properties such as thickness and porosity [36,38,40,43]. These observations suggest the presence of a pre-ordering phase preceding bulk crystallization, a regime that merits further investigation [5,53].

9. The Authors employ different substrates (i.e., Ru(0001) and Cu(111)), which have different impacts on the temperature and timescales. Why? This should be clarified

more extensively.

Author Reply:

We thank the reviewer for this valuable suggestion. We have expanded the discussion to clarify both the origin of the substrate-dependent crystallization temperatures and timescales and the motivation for employing different substrates.

It is well established that different substrates can lead to ASW films with different structural properties, such as morphology, porosity, and interfacial order, due to variations in substrate-water interactions and deposition dynamics (e.g., ***Cryst. Growth Des.*** *25, 978 – 985* ***(2025)****;* ***Phys. Chem. Chem. Phys.*** *22, 1963 – 1973* ***(2020)****;* ***J. Chem. Phys.*** *126, 181103* ***(2007)***). As comprehensively reviewed by Tonauer et al. (***J. Chem. Phys.*** *158, 141001* ***(2023)***), the crystallization kinetics of vapor-deposited ASW are highly sensitive to these structural characteristics. Consequently, both the absolute crystallization temperature and the associated timescale naturally vary from one substrate to another. These substrate-induced differences affect both nucleation and growth rates, but they do not alter the central qualitative conclusion of our study: a transient excess of surface dangling OD bonds consistently appears during ASW crystallization, provided that the kinetic competition between the crystallization rate k and the surface stabilization rate r_{stbl} satisfies the conditions discussed in the main text.

Moreover, even on the same substrate, small variations in the ASW deposition temperature can lead to noticeable differences in bulk crystallization kinetics, as shown in **Supplementary Fig. 9c**. This observation demonstrates that subtle differences in the initial ASW structure can result in significantly different crystallization behaviors.

Our primary motivation for employing substrates with different surface chemistries and crystallographic orientations is to demonstrate that the transient formation of excess dangling OD bonds during ASW crystallization is a universal phenomenon rather than a substrate-specific effect. Consistent qualitative behavior is observed on both Ru(0001) and Cu(111) (**Fig. 3a** and **Supplementary Fig. 6d**). Additional measurements performed on Au(100) likewise show the same transient excess of dangling OD bonds (**Supplementary Figs. 7 – 8**), further supporting the generality of our findings.

We have added clarifying text addressing these points in both the main manuscript and the Supplementary Information.

Revision in the manuscript:

Page 2, line 73-76:

Thick ASW and CI films were prepared by vapor deposition onto Ru(0001), Cu(111), and Au(100) substrates under UHV (base pressure ~1×10^{-10} torr) [28]. Using multiple substrates allows us to demonstrate that the transient formation of excess dangling OH bonds during ASW crystallization is a universal surface phenomenon, independent of substrate hydrophilicity or crystallographic orientation, as detailed below and in the **Supplementary Information**.

Revision in the Supplementary Information:

(1) We have added one section in the **Supplementary Text.**

4. Use of different substrates in the study of excess dangling OD bonds during ASW crystallization

To establish the universality of excess dangling OD bond formation during ASW crystallization, ASW films were also prepared on Cu(111) and Au(100) substrates (**Supplementary Figs. 1** and **6 – 9**). All substrates exhibit the same qualitative behavior, demonstrating that the phenomenon is independent of substrate hydrophilicity or crystallographic orientation.

Nevertheless, different substrates generate ASW films with different structural properties, such as the morphology, porosity, and interfacial order, due to differences in substrate-water interactions and deposition dynamics [7-9]. As extensively summarized by Tonauer et al.,[10] crystallization kinetics in vapor-deposited ASW are highly sensitive to these structural properties. Consequently, both the crystallization temperature and the characteristic timescale vary across substrates and deposition conditions. Although these substrate-induced differences influence nucleation and growth rates, they do not alter the central qualitative conclusion of this work: a transient excess of surface dangling OD bonds consistently emerges during ASW crystallization, provided that the competition between the crystallization rate k and the stabilization rate r_{stbl} satisfies the conditions discussed in the main text.

(2) **Supplementary Fig. 1** has been revised to include the spectra for the ice films on Au(100) substrate.

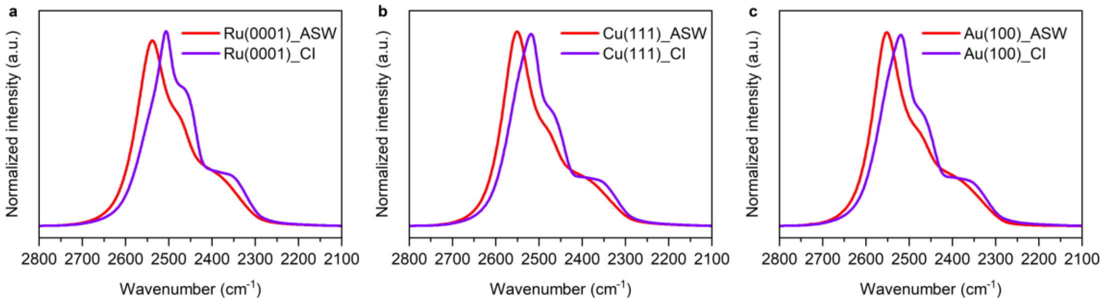

Supplementary Fig. 1. IRRAS spectra in the OD stretching region (ν_{OD}) for ASW and CI films. The spectra were recorded after depositing 100 L D_2O onto (**a**) Ru(0001), (**b**) Cu(111), and (**c**) Au(100) substrates. ASW films were deposited at 100 K on Ru(0001) and Au(100), and at 105 K on Cu(111), respectively. CI films were obtained by annealing the ASW films at elevated temperatures to induce crystallization.

(3) We have added more detailed results from analogous studies on the Au(100) substrate as **Supplementary Figs. 7 – 9.**

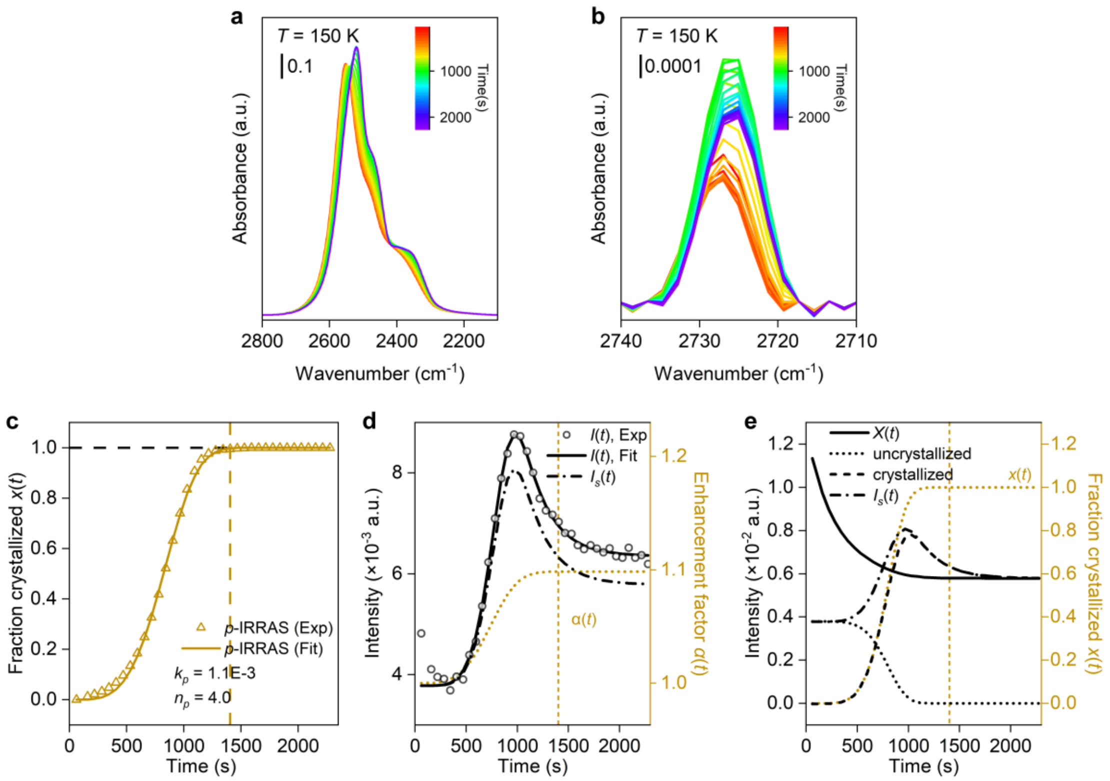

Supplementary Fig. 7. Time-resolved IRRAS analysis of crystallization kinetics for ~100 L ASW films deposited on Au(100) at 100 K. (**a**) *p*-polarized IRRAS spectra in the OD stretching region (ν_{OD}) recorded during isothermal crystallization at 150 K. The initial (red) and final (purple) spectra correspond to the fully amorphous ($x = 0$) and fully crystalline states ($x = 1$), respectively. (**b**) Evolution of the surface-specific free OD mode ($\nu_{\text{free-OD}}$), associated with dOD, during the crystallization process. (**c**) Time-dependent fraction crystallized $x(t)$ extracted from the spectral deconvolution of (**a**) and fitted with the Avrami equation. The dashed vertical line marks the completion of bulk crystallization. (**d**) Temporal evolution of the integrated intensity of the $\nu_{\text{free-OD}}$ mode at 150 K, fitted with the model $I(t) = I_s(t) \cdot \alpha(t)$, as described in the main text. (**e**) Extraction of the surface dOD contribution $I_s(t)$ by accounting for surface stabilization effects, modeled by a logistic decay function $X(t)$.

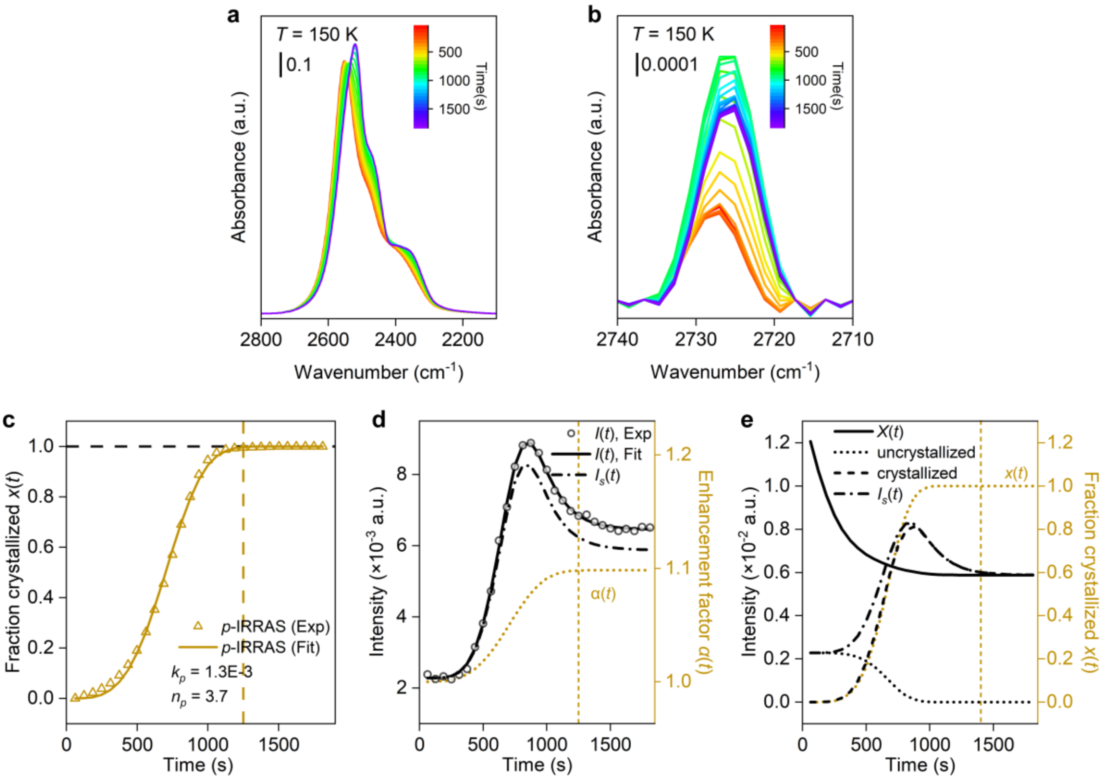

Supplementary Fig. 8. Time-resolved IRRAS analysis of crystallization kinetics for ~100 L ASW films deposited on Au(100) at 105 K. (**a**) p-polarized IRRAS spectra in the OD stretching region (ν_{OD}) recorded during isothermal crystallization at 150 K. The initial (red) and final (purple) spectra correspond to the fully amorphous ($x = 0$) and fully crystalline states ($x = 1$), respectively. (**b**) Evolution of the surface-specific free OD mode ($\nu_{free\text{-}OD}$), associated with dOD, during the crystallization process. (**c**) Time-dependent fraction crystallized $x(t)$ extracted from the spectral deconvolution of (**a**) and fitted with the Avrami equation. The dashed vertical line marks the completion of bulk crystallization. (**d**) Temporal evolution of the integrated intensity of the $\nu_{free\text{-}OD}$ mode at 150 K, fitted with the model $I(t) = I_s(t) \cdot \alpha(t)$, as described in the main text. (**e**) Extraction of the surface dOD contribution $I_s(t)$ by accounting for surface stabilization effects, modeled by a logistic decay function $X(t)$.

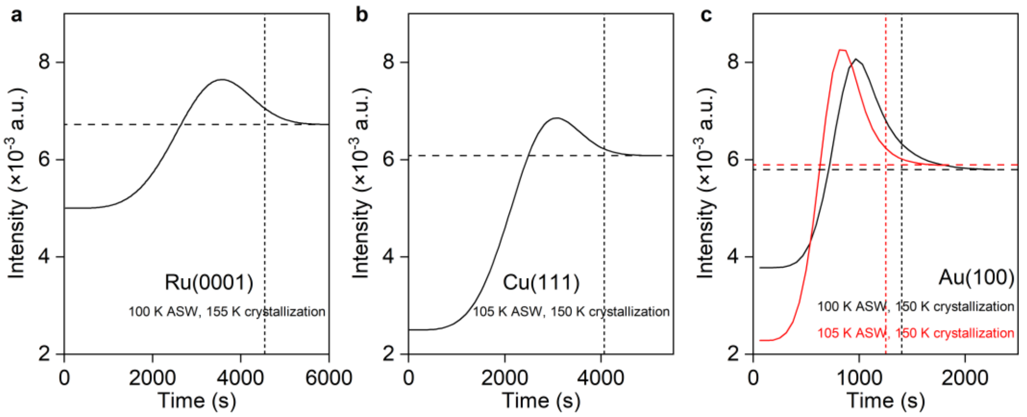

Supplementary Fig. 9. Summary of the experimentally derived surface-specific

component $I_s(t)$, which is directly proportional to the dOD population, during bulk crystallization. (**a**) ASW film grown on Ru(0001) at 100 K and rapidly annealed to 155 K for isothermal IRRAS crystallization measurements. (**b**) ASW film grown on Cu(111) at 105 K and rapidly annealed to 150 K for isothermal IRRAS crystallization measurements. (**c**) ASW films grown on Au(100) at 100 K and 105 K and rapidly annealed to 150 K for isothermal IRRAS crystallization measurements. The dashed vertical line indicates the completion of bulk crystallization.

10. Is there any pH-dependent effect on the process, as it might be expected?

Author Reply:

We thank the reviewer for this insightful question. We did not explore pH-dependent effects in the present study, as such effects lie beyond the scope of our current work. However, previous investigations have indeed shown that proton concentration can significantly influence the crystallization kinetics of ASW.

In particular, ASW films doped with small amounts of HCl, which dissociates into excess protons (H^+) and Cl^-, exhibit accelerated crystallization (***J. Phys. Chem. C*** *122, 24164 – 24170 (**2018**)*). By contrast, Na^+ ions slow down crystallization, analogous to the freezing-point depression observed in electrolyte-containing water. These studies demonstrate that excess protons lower the activation energy for crystallization to a value comparable to that of crystal growth, shifting the kinetic bottleneck from nucleation to growth. Similar effects on crystallization have also been reported for HNO_3-doped ASW (***J. Phys. Chem. C*** *111, 10438 – 10447 (**2007**)*).

Importantly, although such dopants modify the nucleation rate (i.e., either promoting or inhibiting nucleation), the overall crystallization kinetics still follow Avrami behavior. In the context of our work, the transient formation of excess dOD arises from the competition between the crystallization rate k (which includes both nucleation and growth contributions) and the surface stabilization rate r_{stbl}. If r_{stbl} does not overwhelmingly exceed k, a condition also discussed in the main text, excess dOD formation may still occur in doped or pH-modified ASW films. Thus, pH-dependent (or more broadly, dopant-dependent) modulation of excess dOD formation is plausible, although quantifying its magnitude requires future dedicated studies.

In response to the reviewer's question, we have added a brief discussion of this point in the revised manuscript.

Revision in the manuscript:

Page 9, line 302-303:

These non-equilibrium features alter the physicochemical landscape of ice surfaces by increasing the population of highly undercoordinated, and therefore highly reactive, surface sites [45,56,57]. Previous studies have also shown that introducing acids (HCl or HNO_3) or salts (NaCl) into ASW can modify the crystallization rate k, and may therefore influence both the magnitude and duration of excess dOD formed during the ASW-to-CI transition [46,58].

11. Line 238: DOB fractions are estimated (Fig. 4a-c). Please indicate the limitations and potential draback of those indirect estimates.

Author Reply:

We thank the reviewer for raising this important point. We agree that the dOD fractions presented in **Fig. 4a – c** are indirect estimates and therefore subject to several inherent limitations. These arise both from the calibration procedure and from the assumptions required to connect the IRRAS signal to the actual surface dOD population. We summarize the main limitations below:

(1) Indirect referencing to NC-AFM measurements. As discussed in the manuscript, *directly linking* $\nu_{free\text{-}OD}$ *intensity* $I_S(t)$ *to the absolute dOD fraction* $f(t)$ *remains challenging*. Our calibration relies on previously reported NC-AFM measurements (***Nature*** *630, 375 – 380 (**2024**)*). The limitation of this approach is that the two techniques operate under slightly different conditions: NC-AFM images are acquired at 4 K after rapid quenching from the annealing temperature (e.g., 152 K), yielding a quasi-static structural snapshot, whereas IRRAS spectra are obtained *in situ* at elevated temperatures, where the surface hydrogen-bond network is highly dynamic and the dOD population is time-averaged. These differences may introduce small deviations between the inferred and actual dOD fractions.

(2) Comparison with SFG/MD studies. We also compared our IRRAS-derived estimates with SFG measurements performed at 150 – 250 K under ambient pressure, where free OH intensities were converted into population fractions using MD-based correlations (***Phys. Rev. Lett.*** *119, 133003 (**2017**)*). These SFG-derived values are themselves indirect and rely on simulation-dependent calibration. Our dOD estimates qualitatively agree with the SFG/MD studies, although the minimum fraction reported in SFG occurs at a higher temperature (~200 K). This discrepancy likely reflects differences between UHV IRRAS conditions (this work) and ambient-pressure SFG experiments.

(3) Assumption of linear proportionality between IR intensity and dOD population. Our approach assumes a linear relationship between the integrated $\nu_{\text{free-OD}}$ intensity and the dOD population. Although this assumption is commonly used in IRRAS and SFG analyses (e.g., ***Phys. Rev. Lett.*** *119, 133003 (**2017**)*; ***Surf. Sci. Rep.*** *46, 1 – 308 (**2002**)*), it may not strictly apply under all structural conditions. As discussed in the manuscript, *this approximation neglects effects of bond orientation and intermolecular coupling on signal intensity, but remains valid given the dynamic disorder and vibrational activity of surface water molecules*. In this work, however, the peak positions and FWHM of the $\nu_{\text{free-OD}}$ remain nearly constant throughout crystallization, and MD simulations indicate minimal changes in dOD orientation (**Supplementary Figs. 14 – 16**), supporting the validity of the linear approximation for the systems and temperature ranges investigated here.

In response to the reviewer's comment, we have now added a statement in the revised manuscript explicitly acknowledging these limitations and the semi-quantitative nature of the dOD fraction estimates.

Revision in the manuscript:
Page 7, line 238-244:
Assuming a linear relation between $f(t)$ and $I_s(t)$, the temporal evolution of dOD fraction can be estimated (red lines in **Fig. 4a – c**). These values are semi-quantitative, as the calibration relies on indirect referencing to NC-AFM and the linear approximation neglects possible effects of dipole orientation and intermolecular coupling on the IRRAS intensity. Nevertheless, this approach remains reasonable given the dynamic disorder and vibrational activity of surface water molecules. Similar to SFG, IRRAS probes time-averaged dOD populations. Peak position and full width at half maximum (FWHM) analyses indicate little change in dOD orientation (**Supplementary Figs. 14** and **15**), supported by MD simulations (**Supplementary Fig. 16**).

12. MD simulations. How free OH groups were identified/labeled rigorously?
Author Reply:
We thank the reviewer for this important question. In our MD simulations, free OH groups were identified using a rigorous and widely accepted geometric criterion for hydrogen bonding (***Nature*** *379, 55 – 57* ***(1996)***), and extensively applied in subsequent studies of liquid water, ice, and interfacial hydrogen-bond networks (***J. Phys. Chem. B****, 116, 31, 9467 – 9481* ***(2012)***; ***Phys. Rev. Lett.*** *119, 133003* ***(2017)***; ***J. Chem. Theory Comput.****, 14, 357 – 364* ***(2018)****;* ***Phys. Rev. Lett.****, 121, 246101* ***(2018)***). The procedure is described in detail below.

(1) Definition of hydrogen bonds (HBs). We adopted the commonly used geometric criterion established by Luzar and Chandler (**Supplementary Fig. 22**), in which a hydrogen bond is considered formed if both of the following conditions are satisfied: (i) the distance between the donor and acceptor oxygen atoms is shorter than the first minimum of the O-O radial distribution function (i.e. d < 3.3 Å, **Supplementary Fig. 22b**); (ii) the angle between the hydroxyl group of the donor molecule and the vector connecting the donor oxygen to the acceptor oxygen is smaller than the first minimum in the H-O…O angle distribution function (i.e. α < 37°, **Supplementary Fig. 22c**). These geometric cutoffs are consistent with the definitions used in numerous MD studies of bulk water, ice, and water interfaces (***Science****, 311, 832 – 835* ***(2006)***; ***Phys. Rev. Lett.*** *119, 133003* ***(2017)***; ***J. Chem. Phys.****, 161, 184701* ***(2024)***; ***J. Chem. Theory Comput.****, 20, 9107 – 9115* ***(2024)***).

(2) Identification of HBs. For each hydroxyl group, all oxygen atoms within a 3.3 Å radius of the donor oxygen atom were first labeled as potential acceptors. Then, the H-O…O angle was computed for each potential acceptor. A HB was deemed formed if the angle criterion (<37°) was satisfied; otherwise, no hydrogen bond was assigned.

(3) Labeling free OH groups. The number of hydrogen bonds associated with each hydroxyl group was counted. A hydroxyl group was classified as a free OH (i.e., dangling OH) if it did not form any hydrogen bonds according to the above criteria.

This procedure ensures an unambiguous and reproducible identification of free OH groups and is fully consistent with prior MD simulations of ice and interfacial water

(***J. Phys. Chem. B***, *116, 31, 9467 – 9481* (***2012***); ***J. Chem. Theory Comput.***, *14, 357 – 364* (***2018***); ***Phys. Rev. Lett.***, *121, 246101* (***2018***)).

We have expanded the description of this methodology in the revised manuscript and added the corresponding supporting figures in the Supplementary Information to improve clarity and reproducibility.

Revision in the manuscript:

Page 11, line 393-399:

Hydrogen bonds (HBs) in the MD simulations were identified using a commonly accepted geometrical criterion (**Supplementary Fig. 22**) [66]: (i) the distance between the donor and acceptor oxygen atoms is less than 3.3 Å, and (ii) the angle between the donor O-H bond and the O···O vector (H-O···O) is less than 37°. From the trajectory data, each OH group was examined against all oxygen atoms within 3.3 Å. A free OH was defined as an OH group that did not satisfy the HB criterion with any neighboring molecule. The surface free OH fraction was calculated as the ratio of non-hydrogen-bonded OH groups to the total number of surface OH groups.

Revision in the Supplementary Information:

We have added a new figure (**Supplementary Fig. 22**) to the Supplementary Information.

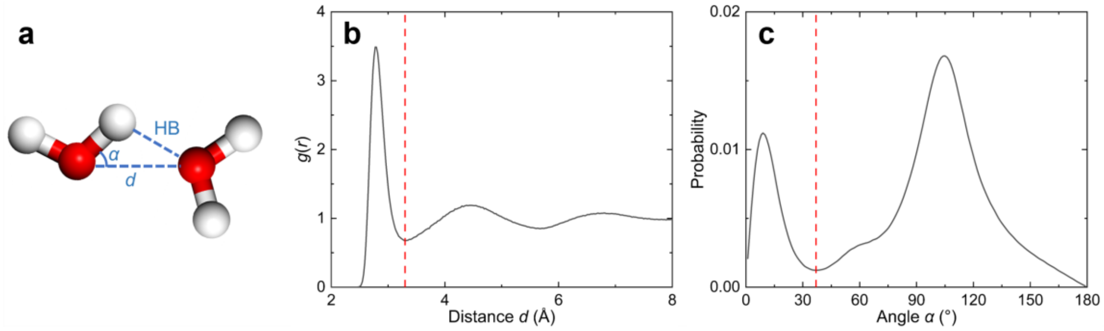

Supplementary Fig. 22. Definition of hydrogen bonds (HB). (**a**) Schematic illustration of a hydrogen bond. (**b**) and (**c**) Criteria used for HB identification in the MD simulations. (**b**) O-O radial distribution function. (**c**) H-O…O angle distribution function. The red dashed lines mark the cutoff values used for HB identification.

13. MD simulations. More importantly, the rigorous identification of the ice-water instantaneous surface is everything but trivial! How the ice surface was identified to count the free OH groups?

Author Reply:

We thank the reviewer for raising this important question. In our MD simulations, we focus on the water-vacuum surface rather than the ice-water interface. Below, we describe (1) the method actually used in this work to identify the instantaneous water-vacuum surface for counting free OH groups, and (2) for completeness, a rigorous method for identifying the ice-water interface using Steinhardt order parameters.

(1) Identification of the water-vacuum surface (method used in this work). To

rigorously determine the instantaneous outer surface where free OH groups reside, we computed the probability density profile of water molecules along the surface-normal direction $\rho(z)$, i.e., along z axis (**Supplementary Fig. 12a** and **b**). The $\rho(z)$ profile was fitted using a standard hyperbolic-tangent interface model (***Phys. Chem. Chem. Phys.**, 23, 13292 – 13299 (**2021**)*):

$$\rho(z) = \rho_0 \left(\tanh\left(\frac{z - z_1}{\delta_1}\right) - \tanh\left(\frac{z - z_2}{\delta_2}\right) \right)$$

where ρ_0 is the average probability density of bulk liquid water, z_1 and z_2 are the center positions of the bottom and top water-vacuum interface, δ_1 and δ_2 represent their interfacial widths, respectively. Therefore, water molecules with z-coordinates less than $z_1 + \delta_1$ or greater than $z_2 - \delta_2$ were identified as surface molecules. Free OH groups were counted only among these surface molecules. The procedure for identifying free OH groups is described above (see our response to Comment 12).

This method provides a well-defined, dynamically fluctuating instantaneous surface and is well suited for quantifying free OH groups exposed to vacuum.

(2) Identification of the ice-water interface (not used here, but provided for completeness). Although our analysis does not rely on the ice-water interface, we also outline here a rigorous method based on the Steinhardt order parameter q_l (***Phys. Chem. Chem. Phys.**, 13, 19807 – 19813 (**2011**)*), which is widely used to classify molecules as "ice-like" or "liquid-like". The q_l parameter for molecule i is defined as:

$$q_l(i) = \frac{1}{N_b(i)} \sum_{j=1}^{N_b(i)} \frac{\boldsymbol{q_l}(i)\boldsymbol{q_l^*}(j)}{|\boldsymbol{q_l}(i)||\boldsymbol{q_l^*}(j)|}$$

where $\boldsymbol{q_l}(i) = [q_{l,-l}(i), q_{l,-l+1}(i), \ldots, q_{l,l}(i)]$ is given by:

$$q_{l,m}(i) = \frac{1}{N_b(i)} \sum_{j=1}^{N_b(i)} Y_{lm}(\theta_{ij}, \varphi_{ij})$$

Here, $N_b(i)$ is the number of neighbor water molecules for molecule i within a cutoff radius of 3.3 Å, Y_{lm} are spherical harmonic functions, and θ_{ij} and φ_{ij} denote the azimuthal and polar angles of the displacement vector between the ith and jth molecules, respectively.

The q_l parameter is widely utilized to classify water molecules as either ice or liquid, and **Fig. R1** shows the q_6 probability distributions of pure liquid and ice systems at 260 K, which exhibit two distinct peaks. The threshold for distinguishing liquid or ice is defined as the intersection point of the distribution curves (i.e. 0.47 at 260 K). Molecules with q_6 less than 0.47 are identified as liquid-like molecules, while with q_6 greater than 0.47 are identified as ice-like molecules. Using this criterion, an instantaneous ice-water interface can be identified. We include this description only to demonstrate methodological rigor; it was not required for the present analysis, which exclusively focused on the water-vacuum surface.

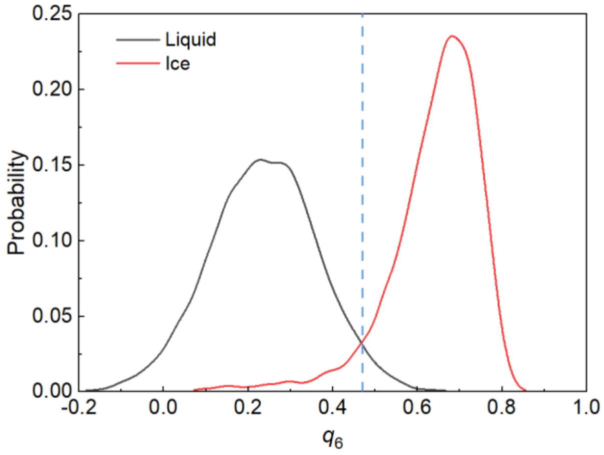

Fig. R1 (for reply). Probability distributions of q_6 order parameters for pure liquid and ice systems. The blue dashed line indicates the threshold used to distinguish liquid-like and ice-like molecules.

In response to the reviewer's comment, we have expanded the description of the surface-identification methodology in the revised manuscript and added corresponding supporting figures in the Supplementary Information to improve clarity and reproducibility.

Revision in the manuscript:

Page 11, line 390:

To rigorously determine the instantaneous position of the outer ice surface where free OH groups reside, we computed the probability density profile of oxygen atoms along the surface-normal direction and fitted it using a standard hyperbolic-tangent interface model [65] (**Supplementary Fig. 12a** and **12b**). NVT simulations at 250 K and 260 K were……

Revision in the Supplementary Information:

(1) We added one section in the **Supplementary Text.**

8. Identification of the water-vacuum interface

To rigorously determine the instantaneous position of the outer surface where free OH groups reside, we computed the probability density profile of oxygen atoms along the surface-normal direction, i.e., along z-axis, yielding $\rho(z)$ (**Supplementary Fig. 12a** and **12b**). The $\rho(z)$ profile was fitted using a standard hyperbolic-tangent interface model [12]:

$$\rho(z) = \rho_0 \left(\tanh\left(\frac{z - z_1}{\delta_1}\right) - \tanh\left(\frac{z - z_2}{\delta_2}\right) \right) \tag{6}$$

where ρ_0 is the average probability density of bulk liquid water, z_1 and z_2 are the center positions of the bottom and top water-vacuum interface, δ_1 and δ_2 represent the corresponding interfacial thicknesses. Water molecules with z-coordinates less than $z_1 + \delta_1$ or greater than $z_2 - \delta_2$ were identified as surface molecules. Free OH groups were counted exclusively within this surface region.

This procedure provides a well-defined, instantaneous surface that fluctuates

dynamically and is appropriate for quantifying free OH groups exposed to vacuum.

(2) We have included the figures related to the water-vacuum interface in **Supplementary Fig. 12.**

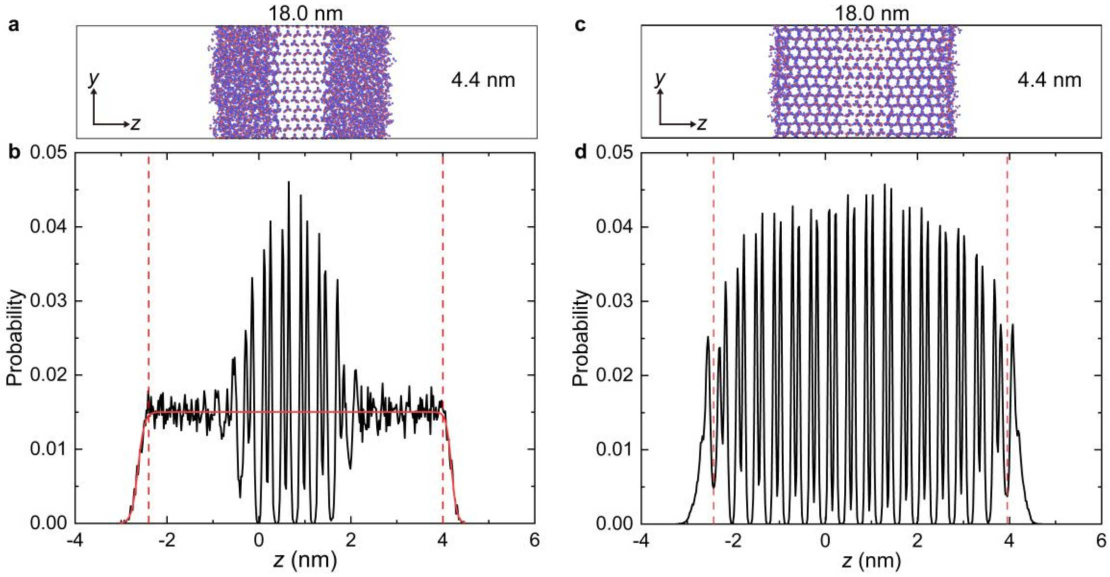

Supplementary Fig. 12. System configuration and identification of the water-vacuum (a, b) and ice-vacuum (c, d) interfaces. (**a**) Representative simulation snapshot of the liquid-ice coexistence system at 260 K. (**b**) Probability density profile of oxygen atoms along the *z*-axis. The red solid line shows the fit to the density profile, and the red dashed lines denote the positions of the bottom and top surfaces. (**c**) Representative simulation snapshot of the ice slab at 260 K. (**d**) Probability density profile of oxygen atom positions along the *z*-axis. The red dashed lines denote the boundaries of the bottom and top ice layers, used to define the extent of the surface region.

14. Supplementary Fig. 3c does not report any data from MD simulation, as erronously stated in the Methods section.

Author Reply:

We thank the reviewer for pointing out this mistake. The original statement incorrectly referred to "**Supplementary Fig. 3c** and **Fig. 8**". This has now been corrected to "**Fig. 3c** and **Supplementary Fig. 11**" in the revised manuscript.

Revision in the manuscript:

Page 11, line 390:

……a simulation cell of 4.05 × 4.4 × 18.0 nm^3 (**Fig. 3c** and **Supplementary Fig. 11**).

15. If possible, please provide a template LAMMPS input file to ensure reproducibility.

Author Reply:

We thank the reviewer for this helpful suggestion. To facilitate reproducibility, we

now provide a template LAMMPS input file together with a post-processing script used for hydrogen-bond identification. These files have been included in the **Supplementary Information.**

LAMMPS input file:

```
# initiallization
units               real
atom_style          full
boundary        p p p
# set temperature
variable        T equal 260
read_data           relax.data
# force field parameter for Tip4p/ice
pair_style          lj/cut/tip4p/long 1 2 1 1 0.1577 10
kspace_style  pppm/tip4p 1.0e-4
pair_coeff          1 1 0.21084 3.1668
pair_coeff          1 2 0.0 0.0
pair_coeff          2 2 0.0 0.0
bond_style          harmonic
bond_coeff          1 1000.0 0.9572
angle_style         harmonic
angle_coeff         1 100.0 104.52
velocity            all create $T 1000
# keep water molecules rigid
Fix             fixshake all shake 0.0001 20 0 b 1 a 1
# set timestep as 2 fs
timestep        2.0
# set NVT ensemble
fix             1 all nvt temp $T $T $(100*dt)
# output thermo file and dump file
thermo              1000
dump                1 all custom 10000 dump/$T.*.dump id type x y z
run             30000000
```

HB identification file implemented in Matlab:

```
clear;clc;
% HB criterion
R = 3.3;      % O-O distance
alpha = 37;   % H-O...O angle
% read LAMMPS output files
filepath='F:\Freezing\Crystallization\250\wwhbonds\';
dumpall=dir(strcat(filepath,'260.*.dump'));
N = length(dumpall);
```

```
file_numbers = zeros(1, N);
% sort the output files by time
for i = 1:NormalizeDataTask
        file_numbers(i) = str2double(regexp(dumpall(i).name, '250\.(\d+)\.dump', 'tokens', 'once'));
end
[~, sort_indices] = sort(file_numbers);
sorted_files = dumpall(sort_indices);

for k = 1:N
        % read the data from the output files
        file=strcat(filepath,sorted_files(k).name);
        dump = fopen(file,'r');
        while feof(dump) == 0
                id = fgetl(dump);
                if (strncmpi(id,'ITEM: TIMESTEP',numel('ITEM: TIMESTEP')))
                        timestep=str2num(fgetl(dump));
                else
                        if (strncmpi(id,'ITEM: NUMBER OF ATOMS',numel('ITEM: NUMBER OF ATOMS')))
                                Natoms=str2num(fgetl(dump));
                        else
                                if (strncmpi(id,'ITEM: BOX BOUNDS',numel('ITEM: BOX BOUNDS')))
                                        x_bound(:)=str2num(fgetl(dump));
                                        y_bound(:)=str2num(fgetl(dump));
                                        z_bound(:)=str2num(fgetl(dump));
                                else
                                        if (strcmpi(id(1:11),'ITEM: ATOMS'))
                                                for i=1:1:Natoms
                                                        atom_data(i,:)=str2num(fgetl(dump));
                                                end
                                        end
                                end
                        end
                end
        end
        atom_data=sortrows(atom_data);
        xl=x_bound(2)-x_bound(1);
        yl=y_bound(2)-y_bound(1);
        zl=z_bound(2)-z_bound(1);

        % select data of oxygen and hydrogen atoms
        data_Oxygen=atom_data(atom_data(:,2)==1,1:5);
```

```
data_Hydrogen=atom_data(atom_data(:,2)==2,1:5);
n_O=size(data_Oxygen,1);
n_H=size(data_Hydrogen,1);

% determine all potential oxygen acceptor around each oxygen atom
donor_A=zeros(10,5,n_O);
for i=1:n_O
    nO=0;
    for j=1:n_O
        if (j~=i)
            d_x = data_Oxygen(i,3) - data_Oxygen(j,3);
            d_y = data_Oxygen(i,4) - data_Oxygen(j,4);
            d_z = data_Oxygen(i,5) - data_Oxygen(j,5);
            [d_x,d_y,d_z]=PBC(d_x,d_y,d_z,xl,yl,zl);
            distance_0=sqrt(d_x^2+d_y^2+d_z^2);
            if (distance_0<R)
                nO=nO+1;
                donor_A(nO,:,i)=data_Oxygen(j,:);
            end
        end
    end
end

% calculate the angle between hydroxyl group and potential acceptor oxygen
n_hbonds=0;
for i=1:n_O
    Donor(i,1)=0;
    for ii=1:2
        dx_OH=data_Hydrogen(2*i+ii-2,3)-data_Oxygen(i,3);
        dy_OH=data_Hydrogen(2*i+ii-2,4)-data_Oxygen(i,4);
        dz_OH=data_Hydrogen(2*i+ii-2,5)-data_Oxygen(i,5);
        [dx_OH,dy_OH,dz_OH]=PBC(dx_OH,dy_OH,dz_OH,xl,yl,zl);
        O_H=[dx_OH, dy_OH, dz_OH];
        n_OO(i)=sum(donor_A(:,1,i)~=0);
        for jj=1:n_OO(i)
            dx_OO=donor_A(jj,3,i) - data_Oxygen(i,3);
            dy_OO=donor_A(jj,4,i) - data_Oxygen(i,4);
            dz_OO=donor_A(jj,5,i) - data_Oxygen(i,5);
            [dx_OO,dy_OO,dz_OO]=PBC(dx_OO,dy_OO,dz_OO,xl,yl,zl);
            O_O = [dx_OO, dy_OO, dz_OO];
            sita = (180/pi)*acos(dot(O_H,O_O)/(norm(O_H)*norm(O_O)));
            % determine whether the angle is less than 37°
            if (sita < alpha)
                n_hbonds= n_hbonds+ 1;
```

```
                    Donor(i,1)=Donor(i,1)+1;
                    dx_HO=donor_A(jj,3,i)-data_Hydrogen(2*i+ii-2,3);
                    dy_HO=donor_A(jj,4,i)-data_Hydrogen(2*i+ii-2,4);
                    dz_HO=donor_A(jj,5,i)-data_Hydrogen(2*i+ii-2,5);
                    [dx_HO,dy_HO,dz_HO]=PBC(dx_HO,dy_HO,dz_HO,xl,yl,zl);
                    length=sqrt(dx_HO^2+dy_HO^2+dz_HO^2);
                    % output the HB information
                    Hbonds_info(n_hbonds,1) = data_Oxygen(i,1);
                    Hbonds_info(n_hbonds,2) = data_Hydrogen(2*i+ii-2,1);
                    Hbonds_info(n_hbonds,3) = donor_A(jj,1,i);
                    Hbonds_info(n_hbonds,4) = length;
                    Hbonds_info(n_hbonds,5) = sita;
                end
            end
        end
    end

    % count the HB number formed of each hydroxyl group
    for i=1:n_H
        HB(i,1)=0;
        for j=1:n_hbonds
            if (Hbonds_info(j,2)==data_Hydrogen(i,1))
                HB(i,1)=HB(i,1)+1;
            end
        end
    end

    % output matrix
    D=zeros(n_H,2);
    D(:,1)=data_Hydrogen(:,1);
    D(:,2)=HB;
    % output filepath
    filename = ['F:\Freezing\Crystallization\250\wwnumber\', (sorted_files(k).name)];
    [fid,message] = fopen(filename,"wt");
    for ii=1:n_H
        for jj=1:2
            fprintf(fid,'%f\t',D(ii,jj));
        end
        fprintf(fid,'\r\n');
    end
    fclose(fid);
    clearvars -except k R alpha filepath sorted_files N;
end
```

```
% periodic boundary function
function [dx,dy,dz] = PBC(dx,dy,dz,xl,yl,zl)
    dx=dx-xl*round(dx/xl);
    dy=dy-yl*round(dy/yl);
    dz=dz-zl*round(dz/zl);
end
```

Reviewer #3 (Remarks to the Author):

1. The manuscript "Formation of excess dangling OH bonds during crystallization of amorphous solid water" by authors Li et al describes an experimental and molecular dynamics study on the crystallization dynamics of deuterated amorphous water ice. The dangling OH (OD) bond, as measured in IRRAS spectra of ices grown on a cold substrate under UHV conditions, is used to probe crystallization, as is highly standard in such experiments. While the subject of the study would potentially be interesting for the physical chemistry of ices, a lack of coherence among results presented in the manuscript, lack of experimental detail provided, and an incoherence of the conclusions drawn compared to the extensive literature in the field mean that this manuscript would require major revisions, including reinterpretation of the dataset, before it could be reviewed for its scientific conclusions and potential novelty.

Author Reply:

We thank the reviewer for the thoughtful and detailed evaluation of our manuscript and for recognizing the potential interest of this work for the physical chemistry of water ices. We appreciate the reviewer's concerns regarding the coherence of the results, the level of experimental detail, and the consistency of our conclusions with the existing literature. Below, we address each of these points in a clear and structured manner.

(1) "Lack of coherence among results presented in the manuscript". We understand that this concern primarily relates to the inconsistency between (i) CI films grown directly at 140 – 150 K and (ii) ASW films that remain amorphous for a finite time when annealed to 150 – 155 K. This issue is addressed in detail in our response to **Comment 3**, where we clarify the roles of deposition temperature, deposition rate, and crystallization kinetics. We show that these observations are fully consistent when kinetic effects are properly considered.

(2) "Lack of experimental detail provided". We agree that additional methodological details were required. As addressed in our responses to **Comments 3** and **4**, the revised manuscript and Supplementary Information now provide comprehensive descriptions of: (a) ASW film preparation conditions and their crystallization protocols; (b) IRRAS acquisition parameters (numbers of scans, scan speed, spectral resolution, and acquisition time); (c) the definition and implementation of time-resolved IRRAS measurements; (d) background correction procedures, including local baseline correction in the $\nu_{\text{free-OD}}$ region, and data-processing methods; (e) validation of the reliable extraction of the extremely weak $\nu_{\text{free-OD}}$ signal. All relevant source data are now provided to ensure full transparency and reproducibility.

(3) "Incoherence of the conclusions compared to the extensive literature". We understand that this concern is mainly associated with the reviewer's question regarding the relatively long crystallization times observed at 155 K. This issue is addressed in detail in our response to **Comments 2** and **3**, where we demonstrate that the observed kinetics are consistent with prior studies when film thickness, substrate effects, and isotopic substitution (D_2O *vs* H_2O) are taken into account.

We thank the reviewer again for the careful reading and constructive critique. After revising the manuscript to incorporate these clarifications, we believe that our

conclusions are now clearly supported by the data. In particular, the transient excess of dangling OD groups during the ASW-to-CI transformation is directly evidenced by our IRRAS measurements, which simultaneously monitor bulk crystallization kinetics and surface structural evolution. The observed bulk crystallization behavior is fully consistent with established literature. Moreover, the proposed mechanism, based on the competition between the crystallization rate k and the surface stabilization rate r_{stbl}, successfully explains the experimental observations and is further supported by the MD simulations. Together, these results reveal the emergence of a dynamic, metastable interfacial phase and provide mechanistic insight into low-temperature ice surface chemistry relevant to astrochemical and atmospheric processes.

2. Decades of spectroscopic studies of water (H2O) ices deposited under HV and UHV conditions have shown that the transition from metastable amorphous ice to crystalline ice CI occurs rapidly and at temperatures below 150 K [see e.g. Hagen, Tielens & Greenberg Chem Phys 1981 56 367]. Additional information can be obtained from techniques including mass spectrometry. For example, the exact measured temperature of the 'volcano desorption' from mixed ices, measured via gas phase mass spectrometry and attributed to molecules ejected from the ice upon crystallization, varies as a function of the temperature ramp rate, but is generally observed around 140 K for slow heating [Smith et al PRL 1997 79 909, Collings et al MNRAS 2004 354 1133]. The energies of phase changes of deuterated water (D2O, used in this study) are very similar [Smith et al J Phys Chem A 2011 115 5908].

Author Reply:

We thank the reviewer for these valuable comments and for highlighting the extensive literature reporting ASW crystallization below 150 K under HV and UHV conditions. We fully agree that numerous studies, particularly those employing TPD, isothermal desorption, and various spectroscopic techniques, have reported rapid ASW-to-CI transitions below ~150 K (including the work of Hagen, Tielens & Greenberg, ***Chem. Phys.*** *56, 367 – 379 (**1981**)*). Several of these studies are already cited in our manuscript (e.g., ***J. Phys. Chem. C****, 122, 24164 – 24170 (**2018**); **Phys. Chem. Chem. Phys.** 22, 1963 – 1973 (**2020**); **Cryst. Growth Des.** 25, 978 – 985 (**2025**)*).

(1) However, as summarized in a recent review by Tonauer et al. (***J. Chem. Phys.*** *158, 141001 (**2023**)*), ASW crystallization temperatures and rates vary widely depending on substrate, film thickness, porosity, deposition conditions, and isotopic composition. Accordingly, a broad range of crystallization temperatures has been reported in the literature. Several groups have observed crystallization occurring at temperatures above 150 K for vapor-deposited D_2O films on metal substrates. For example, Smith et al. reported crystallization at 150 – 160 K for 5 – 56 ML D_2O films on Ru(0001) using isothermal desorption measurements (***Surf. Sci.*** *367, L13 – L18 (**1996**)*). Kondo et al. observed crystallization at 152 – 160.5 K for ~50 ML D_2O/Ru(0001) using helium atom scattering and IRRAS, where crystallization at 153 K required ~3500 s (***J. Chem. Phys.*** *127, 094703 (**2007**)*). Moreover, D_2O is known to crystallize approximately 5 K higher than H_2O under comparable conditions (***J. Phys.***

***Chem. A** 115, 5908–5917 (**2011**)*). These results are consistent with the temperatures observed in our work.

(2) The reviewer correctly notes that molecular "volcano desorption" occurs upon crystallization in mixed ices (***Phys. Rev. Lett.** 79, 909 – 912 (**1997**)*; ***Mon. Not. R. Astron. Soc**., 354, 1133 – 1140 (**2004**)*). For example, Smith et al. reported volcano desorption temperatures of 156 K (H_2O) and 161 K (D_2O) for CCl_4 trapped in ~30 ML water ices during the ASW-to-CI transition, and they further demonstrated that increasing ASW film thickness shifts the volcano temperature to higher values (e.g. from 160 K for 15 ML D_2O to 162 K for 60 ML D_2O). These results clearly demonstrate that crystallization in vapor-deposited ASW can occur at temperatures higher than 155 K under certain conditions.

However, it is important to emphasize that volcano desorption temperatures depend strongly on the identity and concentration of guest molecules. In some cases, guest species can substantially accelerate crystallization (e.g., ***Phys. Chem. Chem. Phys.**, 19, 17677 – 17684 (**2017**)*). Moreover, volcano desorption does not correspond to the completion of crystallization: desorption typically ceases before the "crystallization front" has propagated through the entire film. Thus, volcano temperatures usually reflect the onset of crystallization.

(3) It is also important to recognize that different experimental techniques probe different aspects of the crystallization process. TPD, isothermal desorption, and helium atom scattering are surface-sensitive and predominantly probe the crystallization occurring at or near the ice-vacuum interface (***J. Chem. Phys.** 127, 094703 (**2007**)*). In contrast, IRRAS probes the full film thickness, and is therefore more sensitive to bulk crystallization, which can proceed more slowly than surface crystallization under certain conditions (***Surf. Sci.** 652, 350 – 354, (**2016**)*). Consequently, differences in the apparent crystallization temperatures measured by these techniques are expected.

(4) In our own measurements, the crystallization kinetics exhibit a substrate dependence: for ~100 nm ASW/Ru(0001), crystallization at 155 K completes in ~4600 s; for ~100 nm ASW/Cu(111), crystallization at 150 K requires ~4100 s; and for ~100 nm ASW/Au(100) (**newly added experiments, Supplementary Figs. 7 – 8**), crystallization at 150 K completes within ~1400 s. All experiments were performed using identical procedures, and the observed differences arise from substrate-induced variations in ASW morphology and porosity, consistent with previous reports (***Phys. Chem. Chem. Phys.**, 24, 26200 – 26210 (**2022**)).*

Although the crystallization temperature and kinetics vary with substrate and with initial ASW film structure, our principal conclusion remains robust: a transient excess of dangling OD groups is consistently observed during the ASW-to-CI transition on all substrates studied, Ru(0001), Cu(111), and Au(100) (**Fig. 3**, and **Supplementary Figs. 6 – 8**). This substrate independence strongly supports the universality of the phenomenon.

In response to the reviewer's comment, we have expanded the relevant discussion in the revised manuscript to further clarify the differences in crystallization temperatures.

Revision in the manuscript:

Page 2, line 73-76:

Thick ASW and CI films were prepared by vapor deposition onto Ru(0001), Cu(111), and Au(100) substrates under UHV (base pressure ~1×10^{-10} torr) [28]. Using multiple substrates allows us to demonstrate that the transient formation of excess dangling OH bonds during ASW crystallization is a universal surface phenomenon, independent of substrate hydrophilicity or crystallographic orientation, as detailed below and in the **Supplementary Information**.

Revision in the Supplementary Information:

(1) We have added one section in the **Supplementary Text.**

4. Use of different substrates in the study of excess dangling OD bonds during ASW crystallization

To establish the universality of excess dangling OD bond formation during ASW crystallization, ASW films were also prepared on Cu(111) and Au(100) substrates (**Supplementary Figs. 1** and **6 – 9**). All substrates exhibit the same qualitative behavior, demonstrating that the phenomenon is independent of substrate hydrophilicity or crystallographic orientation.

Nevertheless, different substrates generate ASW films with different structural properties, such as the morphology, porosity, and interfacial order, due to differences in substrate-water interactions and deposition dynamics [7-9]. As extensively summarized by Tonauer et al.,[10] crystallization kinetics in vapor-deposited ASW are highly sensitive to these structural properties. Consequently, both the crystallization temperature and the characteristic timescale vary across substrates and deposition conditions. Although these substrate-induced differences influence nucleation and growth rates, they do not alter the central qualitative conclusion of this work: a transient excess of surface dangling OD bonds consistently emerges during ASW crystallization, provided that the competition between the crystallization rate k and the stabilization rate r_{stbl} satisfies the conditions discussed in the main text.

(2) We have added more detailed results from analogous studies on the Au(100) substrate as **Supplementary Figs. 7 – 9.**

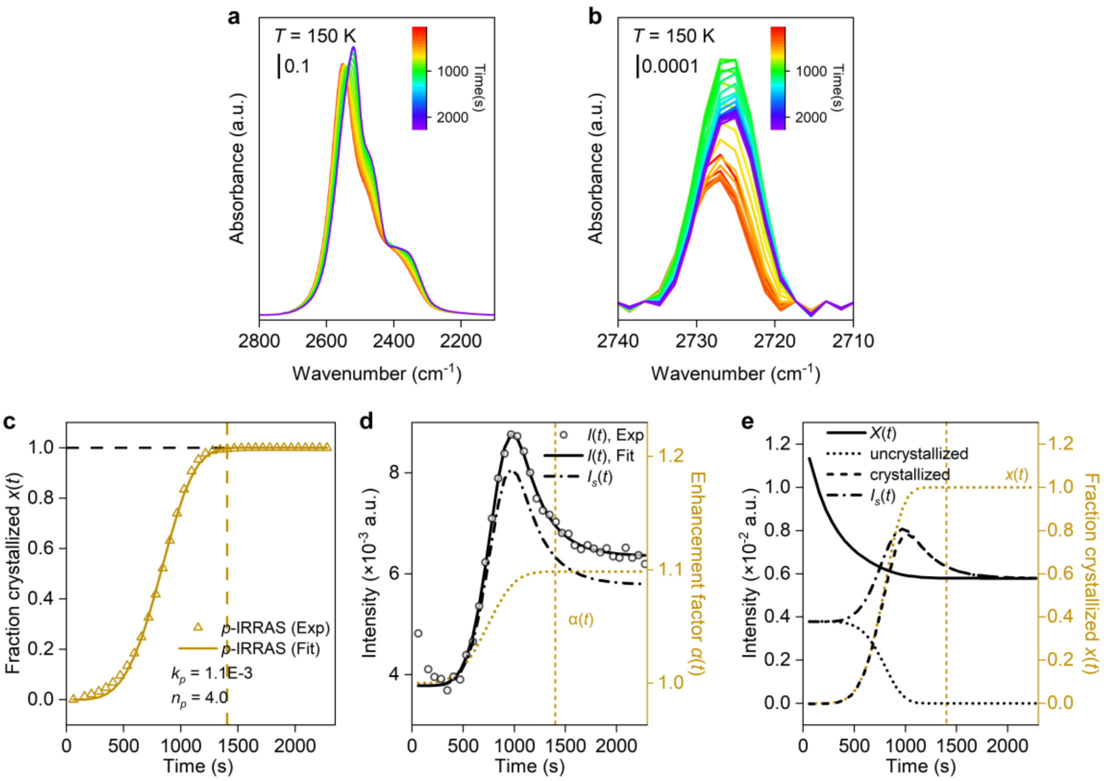

Supplementary Fig. 7. Time-resolved IRRAS analysis of crystallization kinetics for ~100 L ASW films deposited on Au(100) at 100 K. (**a**) *p*-polarized IRRAS spectra in the OD stretching region (ν_{OD}) recorded during isothermal crystallization at 150 K. The initial (red) and final (purple) spectra correspond to the fully amorphous ($x = 0$) and fully crystalline states ($x = 1$), respectively. (**b**) Evolution of the surface-specific free OD mode ($\nu_{free\text{-}OD}$), associated with dOD, during the crystallization process. (**c**) Time-dependent fraction crystallized $x(t)$ extracted from the spectral deconvolution of (**a**) and fitted with the Avrami equation. The dashed vertical line marks the completion of bulk crystallization. (**d**) Temporal evolution of the integrated intensity of the $\nu_{free\text{-}OD}$ mode at 150 K, fitted with the model $I(t) = I_s(t) \cdot \alpha(t)$, as described in the main text. (**e**) Extraction of the surface dOD contribution $I_s(t)$ by accounting for surface stabilization effects, modeled by a logistic decay function $X(t)$.

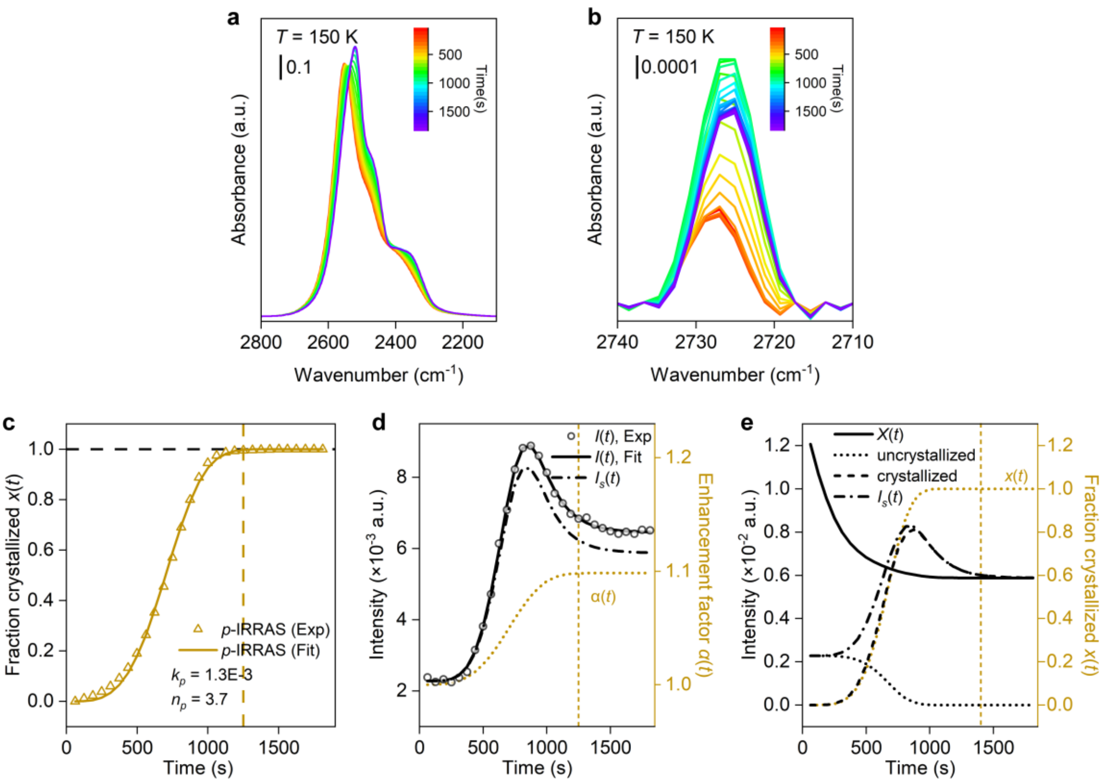

Supplementary Fig. 8. Time-resolved IRRAS analysis of crystallization kinetics for ~100 L ASW films deposited on Au(100) at 105 K. (**a**) *p*-polarized IRRAS spectra in the OD stretching region (ν_{OD}) recorded during isothermal crystallization at 150 K. The initial (red) and final (purple) spectra correspond to the fully amorphous ($x = 0$) and fully crystalline states ($x = 1$), respectively. (**b**) Evolution of the surface-specific free OD mode ($\nu_{free\text{-}OD}$), associated with dOD, during the crystallization process. (**c**) Time-dependent fraction crystallized $x(t)$ extracted from the spectral deconvolution of (**a**) and fitted with the Avrami equation. The dashed vertical line marks the completion of bulk crystallization. (**d**) Temporal evolution of the integrated intensity of the $\nu_{free\text{-}OD}$ mode at 150 K, fitted with the model $I(t) = I_s(t) \cdot \alpha(t)$, as described in the main text. (**e**) Extraction of the surface dOD contribution $I_s(t)$ by accounting for surface stabilization effects, modeled by a logistic decay function $X(t)$.

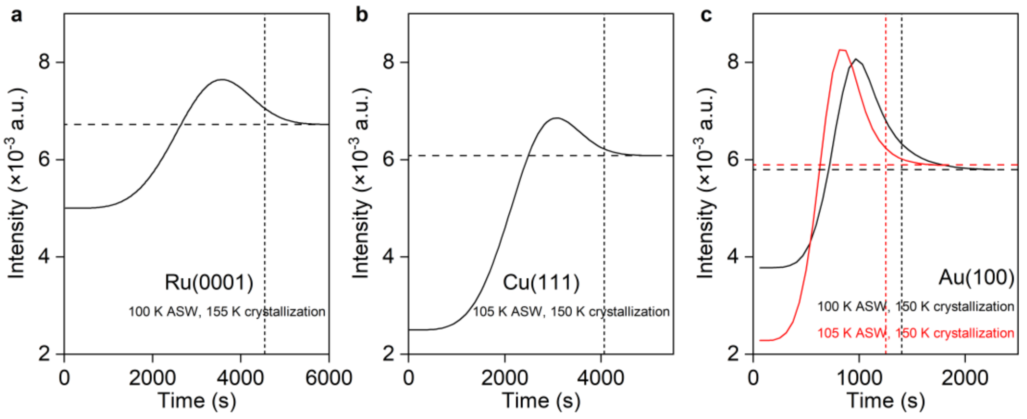

Supplementary Fig. 9. Summary of the experimentally derived surface-specific

component $I_s(t)$, which is directly proportional to the dOD population, during bulk crystallization. (**a**) ASW film grown on Ru(0001) at 100 K and rapidly annealed to 155 K for isothermal IRRAS crystallization measurements. (**b**) ASW film grown on Cu(111) at 105 K and rapidly annealed to 150 K for isothermal IRRAS crystallization measurements. (**c**) ASW films grown on Au(100) at 100 K and 105 K and rapidly annealed to 150 K for isothermal IRRAS crystallization measurements. The dashed vertical line indicates the completion of bulk crystallization.

3. Unless some crucial detail is missing from the Methods section, such as the various experiments on ASW and CI being performed at orders of magnitude different pressures or deposition times, an ice deposited at 100 K and heated to 155 K will crystallize on a timescale significantly more rapid than the >1hour long experiments presented here. The authors have stated that under their experimental conditions, an ice deposited at 140 to 150 K has CI structure. Thus, the conclusion that ices remain amorphous upon timescales on the order of 30-60 minutes at 155 K, and therefore that their crystallization can be measured via IRRAS on that timescale, is not well supported by the data in the current manuscript.

Author Reply:

We thank the reviewer for these valuable comments. In the revised manuscript, we have now added substantially more detail on the experimental procedures and conditions used in our IRRAS measurements. Below we clarify each of the reviewer's concerns.

(1) Experimental details of ASW film preparation and crystallization. All ASW films were grown by vapor-depositing D_2O onto the substrates at a chamber pressure of 2×10^{-8} Torr for 5000 s (base pressure is $\sim1\times10^{-10}$ torr), corresponding to an exposure of ~100 L. The substrate temperatures during deposition were 100 K for Ru(0001), 105 K for Cu(111), and 100 – 110 K for Au(100). In our experimental setup, an exposure of ~0.24 L corresponds to the formation of one ice bilayer (BL) of D_2O (see **Supplementary Text**). Using a nominal bilayer thickness of 0.28 nm, an exposure of 100 L corresponds to a film thickness of ~117 nm.

All crystallization experiments were performed *in situ* under UHV conditions, with a chamber base pressure of $\sim1\times10^{-10}$ torr. During isothermal crystallization, very slow desorption of water molecules caused the chamber pressure to rise slightly to $\sim5\times10^{-10}$ Torr, consistent with the expected desorption behavior of thick ASW films.

In response to the reviewer's comment, we have now added more detail on the experimental procedures of ASW film preparation and crystallization.

Revision in the manuscript:

Page 9, line 316-318:

Typical annealing temperatures were approximately 1300 K for Ru(0001), 770 K for Cu(111), and 800 K for Au(100), yielding atomically clean and well-ordered surfaces.

Page 9, line 325-326:

Deposition was carried out at a pressure of 2×10^{-8} Torr for 5000 s, corresponding to ~100 L exposure (see **Supplementary Text**). ASW films were deposited at 100 K on Ru(0001), at 105 K on Cu(111), and at 100 – 110 K on Au(100) substrates. CI films were grown at elevated substrate temperatures ranging from 140 K to 150 K. Isothermal crystallization experiments were performed *in situ* under UHV conditions. During crystallization, the chamber pressure increased slightly from $\sim1\times10^{-10}$ torr to $\sim5\times10^{-10}$ Torr, consistent with the slow, expected desorption behavior of thick ice films.

(2) IRRAS measurement procedures. After deposition, the ASW films were rapidly annealed (5 – 7 K/s) to the target crystallization temperature (155 K for Ru(0001), 150 K for Cu(111) and Au(100)) and then maintained isothermally. IRRAS acquisition began immediately once the target temperature was reached and continued until crystallization had completed and the signal had fully stabilized. Consecutive spectra were recorded at 1 s intervals, as set in the OPUS software. The spectral acquisition parameters were as follows: for Ru(0001), *p*-IRRAS were collected using 30 scans at a 20 kHz scan speed and 2 cm^{-1} resolution (~27 s per spectrum), while *s*-IRRAS were collected using 60 scans under the same speed and resolution (~54 s per spectrum). For Cu(111) and Au(100), the spectra were recorded using 256 scans at a 40 kHz scan speed and 4 cm^{-1} resolution (~61 s per spectrum).

In response to the reviewer's comment, we have now added more detail on the IRRAS measurement procedures.

Revision in the manuscript:
Page 10, line 338-343:

IRRAS spectra were recorded using both *p*- and *s*-polarized light. Time-resolved isothermal measurements were performed by rapidly annealing the ice films to the target temperatures (5 – 7 K/s) and maintaining them isothermally while continuously acquiring IRRAS spectra, with a 1 s interval between consecutive spectra, as set in the OPUS software. The spectral acquisition parameters were as follows: for Ru(0001), *p*-IRRAS spectra were collected using 30 scans at a 20 kHz scan speed and 2 cm^{-1} resolution (~27 s per spectrum), while *s*-IRRAS spectra were collected using 60 scans under the same speed and resolution (~54 s per spectrum). For Cu(111) and Au(100), the spectra were recorded using 256 scans at a 40 kHz scan speed and 4 cm^{-1} resolution (~61 s per spectrum). The sample temperature was stabilized within ± 0.05 K throughout all measurements. Analysis of the vibrational features, including baseline correction (see **Supplementary Text**), peak position, integrated intensity, and FWHM, was carried out using OPUS and Origin software. The high signal-to-noise ratio of our custom IRRAS setup ($\sim10^{-5}$) enables reliable detection of the $\nu_{\text{free-OD}}$ band, which is intrinsically extremely weak compared with the bulk ν_{OD} stretching mode [30].

(3) Why crystallization at 155 K can require >1 hour for our films. The reviewer suggests that crystallization at 155 K should occur rapidly. In fact, the crystallization times we observed (~4600 s for 100 nm D_2O ASW films on Ru(0001)) are consistent with the literature once film thickness, substrate, and morphology are

properly taken into account. For example, Kondo et al. reported crystallization times of ~3500 s at 153 K and ~1500 s at 156 K for 50 ML (~14 nm) D_2O/Ru(0001) films (***J. Chem. Phys.*** *126, 181103 (**2007**)*; ***J. Chem. Phys.*** *127, 094703 (**2007**)*). Our films are roughly 7 times thicker (~100 nm), and it is well established that crystallization time increases substantially with film thickness (***J. Chem. Phys.*** *150, 214703 (**2019**)*). Thus, a crystallization time of ~4600 s at 155 K can be reasonable.

In addition to Ru(0001), we also examined ~100 nm ASW films on Cu(111) and Au(100). These measurements show substrate-dependent bulk crystallization kinetics: for 100 nm ASW/Cu(111), crystallization at 150 K requires ~4100 s; and for ~100 nm ASW/Au(100) (**newly added experiments**, **Supplementary Figs. 7 – 8**), crystallization at 150 K completes within ~1400 s. All experiments were performed using identical procedures. The observed differences arise from substrate-induced variations in ASW morphology and porosity, consistent with previous reports (***Phys. Chem. Chem. Phys.***, *24, 26200 – 26210 (**2022**))*.

(4) Clarification regarding CI films grown at 140 – 150 K *vs.* the ability of ASW films to remain amorphous at 150 – 155 K. The reviewer notes that ice films deposited at 140 – 150 K are crystalline, which is correct under slow deposition rates. Beyond temperature effect, the structure of vapor-deposited ice depends strongly on deposition rate, flux direction, and surface mobility, which has been extensively documented (e.g., ***Science***, *283, 1505 – 1507 (**1999**)*; ***J. Chem. Phys.***, *110, 5489 – 5492, (**1999**)*; ***J. Geophys. Res.***, *33, 351 – 361 (**2001**)*; ***Atmos. Chem. Phys***., *18, 3419 – 3431 (**2018**)*). In general, low substrate temperature and high deposition rate result in porous ASW, while higher substrate temperature and low deposition rate result in compact ASW or CI. In our experiments, the deposition rate is very low (~100 nm over 5000 s). At this slow rate, D_2O deposited at 140 K indeed grows as CI (**Fig. R2a**).

However, this does not contradict our observation that ASW initially deposited at 100 K can remain amorphous when subsequently heated to 150 K or 155 K for isothermal crystallization. In our experiments, ASW films were deposited at 100 K and then rapidly annealed to the target temperatures. At 150 K, crystallization proceeds very slowly: our measurements show that the film remains predominantly amorphous for ~5000 s (**Figs. 4d** and **Supplementary Fig. 20a**). This behavior is also supported by our kinetic modeling, which predicts that at 150 K, crystallization requires ~23000 s (**Fig. 4h**), whereas at 155 K, crystallization completes in ~4600 s. Thus, it is entirely reasonable that during the initial tens of minutes at 150 K, or the first few minutes at 155 K, the films remain amorphous, in full agreement with our IRRAS observations (**Fig. R2b**). Such a strong temperature dependence of ASW crystallization is well documented in both astrochemical and surface-science studies (***Astrophys. J.***, *473, 1104 – 1113 (**1996**)*).

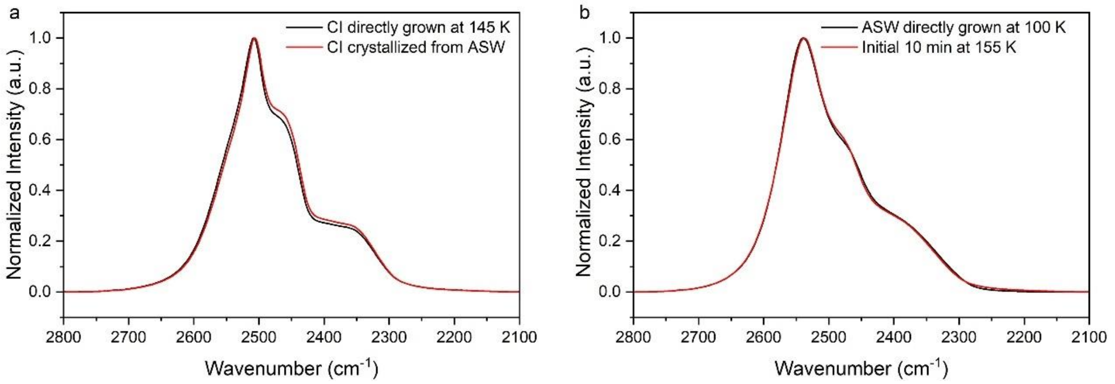

Fig. R2 (for reply). IRRAS spectra show that (**a**) CI films grown directly at 145 K are essentially identical to CI films obtained by crystallization of ASW films annealed at 155 K, and (**b**) ASW films deposited at 100 K retain their bulk amorphous character during the first 10 mins after being heated to 155 K.

4. It is recommended that authors revisit their interpretation in the light of studies such as those cited above and others such as [Dohnalek et al J Chem Phys 2000 112 5932]. Some issues that would need to be addressed include the following. The IR absorption band upon which the conclusions are drawn is of very low intensity. Experimental data, including full IR spectra, background correction procedures, and local correction around the DOB peak position should be exhaustively illustrated. The use of the term "time-resolved" with respect to the repeated measurement of IR spectra is undefined in the methods section. Details such as how many scans per spectrum are acquired and how long each spectrum takes to acquire are not provided. Full details of the method and the methodologies applied must be given. From the discussion and references provided, it is unclear how the authors draw the conclusion that ice surface restructuring at 155 K "may govern the reactivity of icy surfaces in astrophysical and planetary environments" as stated in the abstract.

Author Reply:

We thank the reviewer for these constructive comments. In response, we have substantially expanded and clarified several aspects of the experimental methodology, data processing procedures, and scientific interpretation in the manuscript. Below, we address each of the reviewer's concerns point-by-point.

(1) Low intensity of the $\nu_{free\text{-}OD}$ band. We fully agree that the $\nu_{free\text{-}OD}$ band is extremely weak compared with the bulk ν_{OD} stretching band. For example, for a 100 nm CI film, the intensity of the $\nu_{free\text{-}OD}$ band is only ~0.006% of that of the bulk ν_{OD} stretch. This is because $\nu_{free\text{-}OD}$ originates exclusively from the surface-specific OD groups, where the ν_{OD} stretch reflects the response of the entire ice film. Owing to this intrinsically low intensity, most previous IR studies have focused primarily on bulk crystallization signatures rather than surface-specific vibrational modes.

Nevertheless, due to the exceptionally high signal-to-noise ratio of our custom-built IRRAS setup (with a relative detection limit on the order of ~10^{-5}, for example, demonstrated previously in measurements of CO adsorption on oxide surfaces with ~8

min acquisition time per spectrum (***Precis. Chem.**, 6, 337 – 347 (**2025**)*)), we are able to reliably detect and quantitatively track small but systematic variations in the $\nu_{\text{free-OD}}$ band during crystallization. Importantly, this capability allows us to monitor the evolution of both bulk and surface structures simultaneously within the same experiment.

In response to the reviewer's comment, we have expanded the relevant methodological description in the revised manuscript.

Revision in the manuscript:

Page 9, line 343:

The high signal-to-noise ratio of our custom IRRAS setup (~10^{-5}) enables reliable detection of the $\nu_{\text{free-OD}}$ band, which is intrinsically extremely weak compared with the bulk ν_{OD} stretching mode [30].

(2) Additional methodological details, full IR spectra, background correction procedures, local correction around the $\nu_{\text{free-OD}}$ region, and clarification of "time-resolved IRRAS. We thank the reviewer for these valuable suggestions regarding the completeness and clarity of the experimental methodology.

Representative full IR spectra of both ASW and CI films are now provided in the Supplementary Information, allowing direct inspection of the raw spectral features over the entire $\nu_{\text{free-OD}}$ region (**Supplementary Text**). In addition, all source data for the full IR spectra are made available.

The baseline correction procedure is now described in detail. Specifically, the ν_{OD} stretching region (2100 – 2800 cm^{-1}) is corrected using a rubberband algorithm with 64 anchor points, whereas the $\nu_{\text{free-OD}}$ region is corrected using 10 anchor points. The rubberband algorithm treats the baseline as a convex "envelope" that follows the lower edge of the spectrum, making it robust against broad overlapping absorption bands and largely insensitive to peak shape variations. This procedure yields clean, baseline-flattened spectra and improves the reliability of peak integration and quantitative analysis. To demonstrate the robustness of the analysis, we additionally provide comparisons using more anchor points, all of which lead to consistent qualitative trends and conclusions, as shown in **Supplementary Fig. T2**.

The reviewer correctly notes that the term "time-resolved" was insufficiently defined in the original manuscript. This point has now been clarified in the revised Methods section. In the present work, time-resolved IRRAS refers to the continuous acquisition of successive IR spectra immediately after the sample reaches the target temperatures. The interval between two consecutive spectra is 1 s. The spectral acquisition parameters are now explicitly stated. For Ru(0001), *p*-IRRAS spectra were collected using 30 scans at a scan speed of 20 kHz and a spectral resolution of 2 cm^{-1} (~27 s per spectrum), while *s*-IRRAS were collected using 60 scans under the same conditions (~54 s per spectrum). For Cu(111) and Au(100), the spectra were recorded using 256 scans at a scan speed of 40 kHz and a resolution of 4 cm^{-1} (~61 s per spectrum).

Revision in the manuscript:

Page 10, line 339-343:

Time-resolved isothermal measurements were performed by rapidly annealing the ice films to the target temperatures (5 – 7 K/s) and maintaining them isothermally while continuously acquiring IRRAS spectra, with a 1 s interval between consecutive spectra, as set in the OPUS software. The spectral acquisition parameters were as follows: for Ru(0001), *p*-IRRAS spectra were collected using 30 scans at a 20 kHz scan speed and 2 cm^{-1} resolution (~27 s per spectrum), while *s*-IRRAS spectra were collected using 60 scans under the same speed and resolution (~54 s per spectrum). For Cu(111) and Au(100), the spectra were recorded using 256 scans at a 40 kHz scan speed and 4 cm^{-1} resolution (~61 s per spectrum). The sample temperature was stabilized within ± 0.05 K throughout all measurements. Analysis of the vibrational features, including baseline correction (see **Supplementary Text**), peak position, integrated intensity, and FWHM, was carried out using OPUS and Origin software.

Revision in the Supplementary Information:

We have added one section in the **Supplementary Text.**

2. Background correction procedures applied to the IRRAS spectra

The rubberband algorithm was employed for background correction because it treats the baseline as a convex “envelope” that follows the lower edge of the spectrum, making it robust against broad overlapping absorption bands and largely insensitive to variations in peak shape. This procedure yields clean, baseline-flattened spectra and improves the reliability of peak integration and quantitative analysis. To assess the influence of the rubberband parameters, we additionally provide comparisons using a larger number of anchor points, which yield consistent trends and conclusions**.**

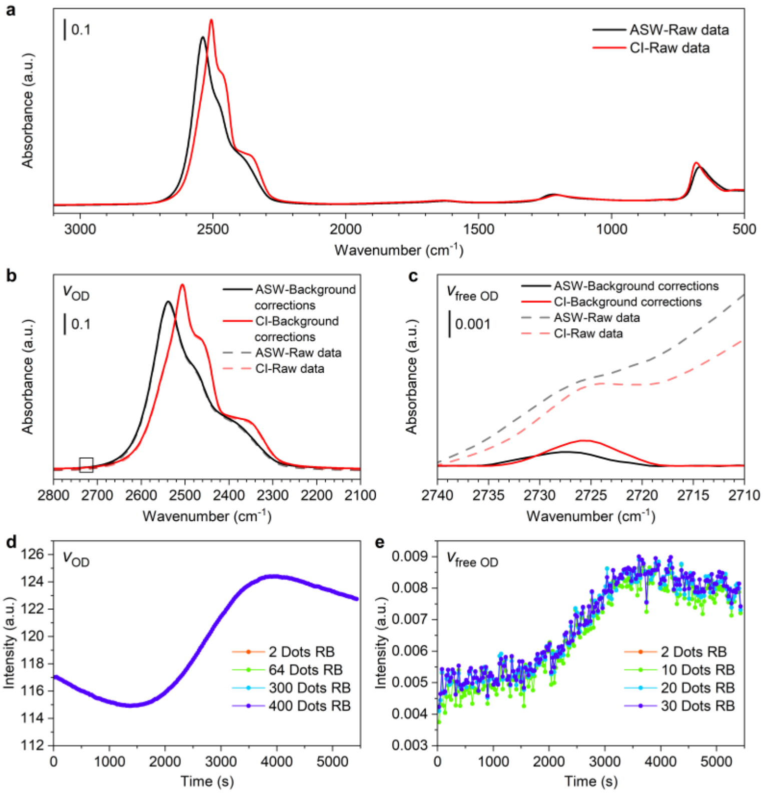

Supplementary Fig. T2. (**a**) Full IRRAS spectra of thick ASW and CI films, showing the stretching, bending and librational vibrational regions. (**b, c**) IRRAS spectra of the ν_{OD} and $\nu_{free\text{-}OD}$ regions for thick ASW and CI films before and after background corrections using the rubberband algorithm with 64 and 10 anchor points, respectively. (**d, e**) Comparison of the integrated peak areas of the ν_{OD} and $\nu_{free\text{-}OD}$ bands obtained using the rubberband algorithm with different numbers of anchor points. The spectra used in (**d**) and (**e**) are identical to those shown in **Fig. 2a** and **Fig. 3a** of the main manuscript.

(3) Interpretation of the conclusion that ice surface restructuring during crystallization may govern the reactivity of icy surfaces in astrophysical and planetary environments. We thank the reviewer for raising this important point. We have now revised the manuscript to provide a clearer and more rigorous justification for this statement.

First, dangling OH groups on ice surfaces are widely recognized as catalytically active sites that facilitate key surface reactions relevant to astrochemistry and planetary science. Prior studies, including laboratory experiments and theoretical simulations, have demonstrated that dOH groups promote reactions such as H_2 formation (***Astrophys. J.*** *682, L69* ***(2008)****;* ***J. Chem. Phys.*** *129, 044501* ***(2008)***), CO_2 formation (***Astrophys. J.*** *567, 651* ***(2002)****;* ***J. Phys. Chem. A*** *115, 4867 – 4873* ***(2011)****;* ***J. Phys. Chem. Lett****. 3, 2482 – 2486 (*2012*)*), and the generation of simple organics including H_2CO and CH_3OH (***Astrophys. J.*** *668, 1001* ***(2007)****;* ***Chem. Rev.*** *113, 8783–8839* ***(2013)***). These reactivities correlate strongly with the density of undercoordinated surface sites.

Second, ice surfaces above ~140 K are known to undergo rapid reorientation and continuous hydrogen-bond breaking and reforming, producing a dynamic yet thermodynamically regulated surface. Both experiments and simulations (***J. Chem. Phys.*** *94, 4091* ***(1991)****;* ***Phys. Rev. Lett.*** *119, 133003* ***(2017)***) demonstrate that the average number of dangling OH groups is temperature-dependent and reaches a quasi-equilibrium value at each temperature.

In our work, we find that this quasi-equilibrium is transiently disrupted during ASW-to-CI crystallization. Taking D_2O/Ru(0001) as an example, the equilibrium CI surface contains ~42% dOD, whereas during isothermal crystallization we observe a clear maximum of ~48%, corresponding to an excess dOD population of ~6%. This excess arises exclusively during the non-equilibrium restructuring of the surface hydrogen-bond network and disappears once crystallization and surface stabilization are complete. These transiently formed dOD represent highly undercoordinated and therefore highly reactive sites.

Finally, in astrophysical and planetary environments, ASW is the dominant form of interstellar ice, but CI has also been detected, and CI is widely believed to form from ASW during episodic heating events (***Science*** *311, 1425 – 1428* ***(2006)****;* ***Nature*** *641, 608 – 611* ***(2025)***). Such transitions typically occur at higher temperatures, e.g. above 140 – 160 K, in which we observe the transient excess dOD population (**Fig. 4h**). Because dangling OH groups are critical reactive motifs in interstellar grain-surface chemistry (e.g., reactions leading to H_2, CO_2, H_2CO, CH_3OH, and other key species), a

temporary surge in their concentration during crystallization may enhance specific chemical pathways that are inefficient on fully amorphous or fully crystalline surfaces.

In response to the reviewer's comment, we have expanded the relevant discussion in the revised manuscript to clarify the underlying mechanism and its astrochemical implications.

Revision in the manuscript:

Page 9, line 302-306:

These non-equilibrium features alter the physicochemical landscape of ice surfaces by increasing the population of highly undercoordinated, and therefore highly reactive, surface sites [45,56,57]. Previous studies have also shown that introducing acids (HCl or HNO_3) or salts (NaCl) into ASW can modify the crystallization rate k, and may therefore influence both the magnitude and duration of excess dOD formed during the ASW-to-CI transition [46,58]. These findings have important implications for astrochemical environments. In interstellar and planetary ices, where ASW is the dominant phase but episodic heating can trigger crystallization, transient increases in dOH may temporarily enhance the surface reactivity of icy grains, promoting the synthesis of key species such as H_2, CO_2, H_2CO, CH_3OH, and others, and potentially opening or accelerating reaction pathways that are inefficient on fully amorphous or fully crystalline ice surfaces [59,60].

General response:

During the revision process, we made two minor clarifications and refinements to the originally submitted manuscript that were not explicitly requested by the reviewers. These include the updates to the reference MD data in **Fig. 1d** and a refined treatment of the surface stabilization function $r_{stbl}(T)$ used to simulate excess dOD formation in ASW/Ru(0001) (**Fig. 4h** and **4i**). Importantly, these refinements lead only to minor quantitative changes and do not affect the qualitative trends or the main conclusions of the study.

(1) The reference molecular dynamics (MD) data (hollow squares shown in the inset of **Fig. 1d**) taken from the literature (*Phys. Rev. Lett.* 119, 133003 (2017)) have been re-digitized and updated.

Revised version Original version

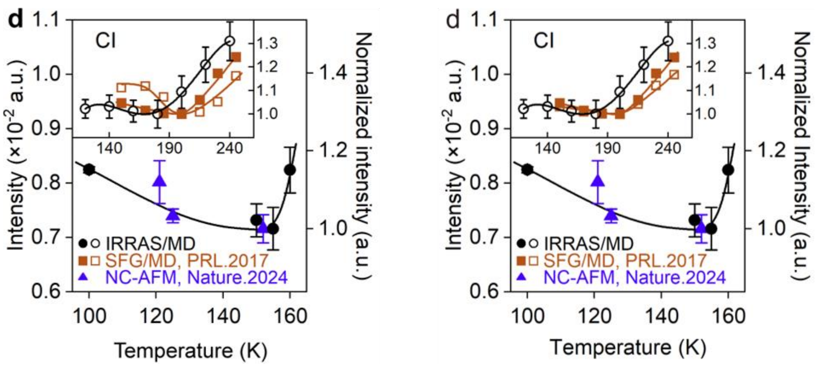

(2) A minor refinement was applied to the fitting procedure used to determine the stabilization function $r_{stbl}(T)$ (**Fig. 3e**), which was subsequently used as an input parameter to simulate excess dOD formation (**Fig. 4h** and **4i**). In the revised analysis, the prefactor and activation energy associated with surface stabilization were extracted from a linear regression of $\text{Ln } r_{stbl} = \text{Ln } A - E_{stbl}/RT$. This approach is more appropriate under the assumption that the uncertainty in r_{stbl} is dominated by multiplicative errors, as is typical for kinetic rate parameters spanning several orders of magnitude. Using this approach, we obtained updated parameters of $A = 2.1\times10^{24}$ and $E_{stbl} = 77.4$ kJ/mol. In the originally submitted version, the parameters (5.8×10^{24} and 79.8 kJ/mol) were obtained from a direct least-squares fit of $r_{stbl} = A\exp(-E_{stbl}/RT)$, which implicitly assumes additive errors in r_{stbl} and is therefore less suitable for the present dataset.

A quantitative comparison further supports the revised fitting approach. The fitting accuracy was evaluated using the root-mean-square error in the logarithmic domain, defined as $\text{RMSE}_{\text{Ln } r_{stbl}} = \sqrt{\frac{1}{n}\sum_{i=1}^{n}\left(\text{Ln } r_{pred,i} - \text{Ln } r_{stbl,i}\right)^2}$, where $\text{Ln } r_{pred,i}$ and $\text{Ln } r_{stbl,i}$ denote the predicted and reference stabilization rates at temperature T_i, respectively. The logarithmic-domain fit yields a smaller error ($\text{RMSE}_{\text{Ln } r_{stbl}} = 0.64$) than the direct fit in the original rate domain ($\text{RMSE}_{\text{Ln } r_{stbl}} = 0.89$), indicating that the

revised approach provides a more balanced and robust description of r_{stbl} across the full temperature range.

Importantly, despite the improved fitting performance, both fitting approaches yield comparable activation energies, and the refinement does not affect the qualitative conclusions of the model. The updated parameters lead to only minor quantitative changes in the simulated curves shown in **Fig. 4h** and **4i**, while the overall trends and the main conclusions of this work remain unchanged. We have added a brief additional section in the **Supplementary Information** that provides a more detailed discussion of **Fig. 4i**.

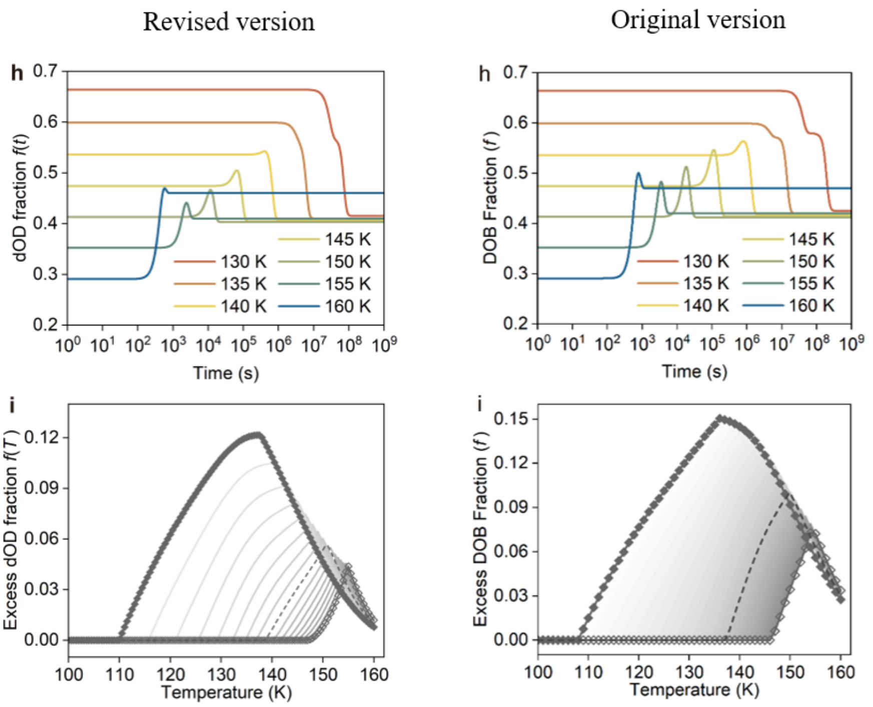

Revision in the Supplementary Information:

We added one section in the **Supplementary Text** to provides a more detailed discussion of **Fig. 4i**.

9. General conditions for the formation of excess dOD during the ASW-to-CI transition

The surface structure of ASW films can vary significantly depending on their thermal history, whereas the surface structure of fully crystallized CI films is considerably more defined and reproducible. This distinction has been demonstrated both by our IRRAS measurements and by previous SFG studies on the temperature-dependent surface structure of CI films [13].

The excess dOD population is defined as the maximum transient dOD intensity observed during the ASW-to-CI transition relative to an appropriate reference dOD intensity. Specifically, when the initial dOD population of the ASW film is lower than that of the final CI surface at the isothermal crystallization temperature, the excess dOD is defined as the difference between the maximum transient dOD intensity and the final CI dOD intensity. Conversely, when the initial ASW dOD population exceeds that of

the final CI surface, the excess dOD is defined as the difference between the maximum transient dOD intensity and the initial ASW dOD intensity.

As discussed in the main text, the emergence of excess dOD during the ASW-to-CI transition is governed by the kinetic competition between surface stabilization and crystallization. Pronounced excess dOD formation occurs when the ratio of the surface stabilization rate to the crystallization rate, $\frac{r_{stbl}}{k}$, falls below a critical threshold. Specifically, excess dOD formation is observed when $\frac{r_{stbl}}{k} < \sim 260$ at 150 K (**Supplementary Fig. 19a**), $\frac{r_{stbl}}{k} < \sim 180$ at 155 K (**Fig. 4g**), and $\frac{r_{stbl}}{k} < \sim 80$ at 160 K (**Supplementary Fig. 19b**). These differences arise from the distinct temperature dependences of the stabilization rate $r_{stbl}(T)$ and the crystallization rate $k(T)$, which are further influenced by the detailed structural properties of the initial ASW films.

Several key features can be extracted from **Fig. 4i** (see also **Supplementary Fig. T4**). First, the magnitude of excess dOD formation during the ASW-to-CI transition spans a broad range, primarily bounded by the solid and hollow diamonds. This wide range mainly reflects variations in the initial surface configurations of the ASW films.

Second, for a given initial ASW surface configuration, each curve in **Fig. 4i** represents the evolution of the dOD population during isothermal crystallization over a range of temperatures. The maximum excess dOD occurs at temperatures for which the initial dOD fraction of the ASW film is closest to that of the final CI surface.

Third, when examined at a fixed isothermal crystallization temperature, the magnitude of the transient excess dOD depends strongly on the initial surface configuration of the ASW film. Based on the relative magnitudes of the initial ASW and final CI surface dOD populations, three temperature regimes can be identified. Approximate boundaries at ~138 K and ~155 K are inferred from **Supplementary Fig. T4** as empirical divisions between distinct regimes of excess dOD behavior; these temperatures correspond to the maxima in the excess dOD fraction associated with the ASW films exhibiting specific surface configurations, as indicated by the solid and hollow diamonds. In the low-temperature regime (< ~138 K), where the initial ASW dOD population always exceeds that of the final CI surface, ASW films with higher initial dOD fractions exhibit smaller excess dOD formation during crystallization. In the intermediate temperature regime (138 – 155 K), where the initial ASW dOD population becomes comparable to that of the final CI surface, the maximum excess dOD occurs when the initial ASW and final CI dOD populations are approximately equal. In the high-temperature regime (> ~155 K), where the initial ASW dOD population is always lower than that of the final CI surface, ASW films with higher initial dOD fractions exhibit larger excess dOD formation during crystallization.

Finally, at very low temperatures crystallization becomes exceedingly slow; for example, at ~130 K crystallization may require up to about 10^8 s, and below ~110 K crystallization is kinetically suppressed, resulting in the absence of excess dOD formation. At the opposite extreme, at sufficiently high temperatures both crystallization and surface stabilization proceed rapidly, again suppressing the

formation of excess dOD.

In conclusion, the formation of excess dOD during the ASW-to-CI transition can be regarded as a universal phenomenon, provided that the kinetic competition between crystallization and surface stabilization satisfies the conditions outlined in the main text.

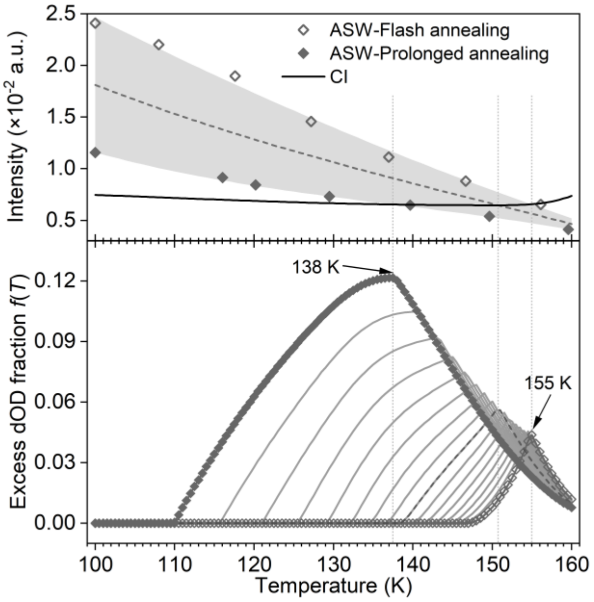

Supplementary Fig. T4. Illustration of the magnitude of transient excess dOD formed during the ASW-to-CI transition as a function of isothermal crystallization temperature and the detailed initial surface configuration of the ASW film.

Point-by-point responses to the reviewers' comments

Reviewer comments are shown in **blue**; author responses are shown in **black**; revisions to the manuscript are highlighted in **red**.

Reviewer #2 (Remarks to the Author):

The Authors, in my opinion, made a thorough review of the article and SI. Thus, the manuscript can now be accepted as it stands.

Author Reply:

We sincerely thank the reviewer for the positive comments and the constructive suggestions that have helped improve the manuscript.

Reviewer #4 (Remarks to the Author):

I was asked to review the authors responses to previous reviewer 3.

I am an IR spectroscopist with a particular focus on liquid water and its interactions with other materials. As such, I am familiar with the liquid water literature, but not as familiar with the extensive ice literature. It seems that most of the comments from all reviewers concerns particular disagreements of the present manuscript and this extensive literature.

Since I am not as familiar with this literature, I will refrain from commenting on this. Although I would say that it seems like the authors have done an extensive review of the literature. This includes the papers of Geissler on the isosbestic point of water, where I will agree with the authors that it is likely different for the present study.

However, although the authors now have provided further specific details on how the experiments were performed, the background subtraction method is not adequately described.

The added text now simply states that the "The rubberband algorithm was employed for background correction because it treats the baseline as a convex "envelope" that follows the lower edge of the spectrum, making it robust against broad overlapping absorption bands and largely insensitive to variations in peak shape. This procedure yields clean, baseline-flattened spectra and improves the reliability of peak integration and quantitative analysis. To assess the influence of the rubberband parameters, we additionally provide comparisons using a larger number of anchor points, which yield consistent trends and conclusions."

However, this is not a description of what the method is or how it affects the data. I am not familiar with the method and there is no reference for it.

Is this an in-build function in the spectrometer or a home-built routine? In either way the method needs to be adequately described and validated.

Simply stating that "This procedure yields clean, baseline-flattened spectra and improves the reliability of peak integration and quantitative analysis.", does not make it true.

Author Reply:

We thank the reviewer for the thoughtful evaluation of our revised manuscript and response, and for raising the important issue concerning the description and validation of the baseline subtraction method. We agree that the procedure should be described clearly, as inappropriate baseline correction may distort spectral features and affect quantitative analysis.

The rubberband baseline correction used in this work is not a home-built routine

but the standard implementation provided in the OPUS software package (Bruker Optics GmbH), which was used to process the IRRAS spectra. This method is widely employed in vibrational spectroscopy for removing slowly varying background signals (*ACS Appl. Mater. Interfaces* 2012, 4, 8, 3916 – 3922; *J. Phys. Chem. C* 2014, 118, 22, 12000 – 12009; *Anal. Methods,* 2019,11, 5195 – 5202).

The method estimates the baseline by constructing the lower convex envelope of the measured spectrum. Conceptually, the procedure can be visualized as stretching an elastic band underneath the spectrum such that it touches the lowest points of the data; the resulting curve defines the estimated baseline.

To illustrate this procedure more clearly, a schematic diagram of the rubberband baseline correction is provided in **Fig. R1**. In this method, the original spectrum is first divided into N segments, where N corresponds to the user-defined parameter "Number of baseline points." Within each segment, the lowest intensity point is selected as a sampling point (red markers), representing candidate baseline points. A convex-hull algorithm is then applied to these points to determine the lower envelope of the spectrum, which defines the rubberband baseline (orange line). This baseline is subsequently subtracted from the original spectrum (blue line) to obtain the baseline-corrected spectrum (green curve). The diagram therefore illustrates how candidate baseline points are determined from local minima in each segment and how the convex hull of these points defines the baseline.

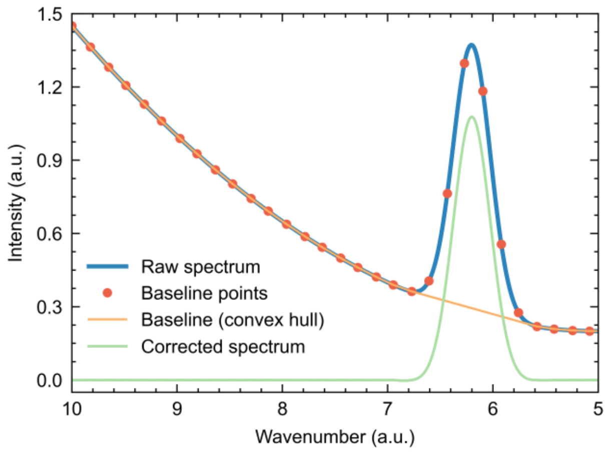

Fig. R1. Schematic illustration of the rubberband baseline correction procedure.

In the OPUS implementation, the parameter referred to as the "Number of baseline points" controls the density of baseline points used to construct the rubberband baseline across the spectrum. This parameter effectively determines how finely the spectral range is segmented when calculating the convex envelope. According to the OPUS documentation, recommended values typically range from 10 to 200, with a default value of 64. Increasing this value increases the density of baseline points and can provide a more detailed baseline representation for spectra with complex background variations.

However, the effective influence of this parameter also depends on both the number of data points in the spectrum and the spectral shape. First, setting a "Number

of baseline points” value larger than 200 or smaller than 10 produces essentially the same baseline as using the boundary values of 200 or 10, respectively. Second, when the raw spectrum is relatively smooth, as is the case for the ν_{OD} band in our data, though not for the $\nu_{free\text{-}OD}$ band, the choice of “Number of baseline points” has only a minor influence on the resulting baseline. Importantly, the positions of these baseline points are determined automatically by the algorithm based on the spectral shape, rather than being manually selected by the user.

An advantage of this approach is that the baseline is determined from the global geometric structure of the spectrum, rather than from local fitting of individual peak shapes. Consequently, slowly varying background contributions (e.g., broad absorption bands or instrumental drift) are captured by the baseline envelope, while sharper vibrational features remain largely unaffected. This reduces the risk of distorting narrow spectral peaks compared with polynomial or aggressive automated fitting procedures.

To further validate the robustness of the method, we performed additional tests by varying the number of baseline points used in the convex-hull construction. These tests show that the resulting baseline changes only slightly (see **Supplementary Fig. T2d-e**). This confirms that the conclusions drawn from the spectral analysis are not sensitive to the specific parameters used in the baseline correction.

Relevant descriptions of this method can be found in: M. Pirzer, K. Sawatski, *Method and device for correcting a spectrum*, US Patent 7359815B2 (2008); S. Wartewig. *IR and Raman Spectroscopy: Fundamental Processing,* Wiley-VCH (2003).

In the revised manuscript and Supplementary Information, we have expanded the description of the background subtraction procedure, clarified how the algorithm is implemented, and added relevant references together with additional validation tests.

Revision in the Supplementary Information:

We have added more discussions in the revised **Supplementary Text 2.**

The rubberband baseline correction used in this work is not a home-built routine but the standard implementation provided in the OPUS software package (Bruker Optics GmbH), which was used to process the IRRAS spectra. This method is widely employed in vibrational spectroscopy for removing slowly varying background signals [5-7].

The method estimates the baseline by constructing the lower convex envelope of the measured spectrum. Conceptually, the procedure can be visualized as stretching an elastic band underneath the spectrum such that it touches the lowest points of the data; the resulting curve defines the estimated baseline.

In the OPUS implementation, the parameter referred to as the “Number of baseline points” controls the density of baseline points used to construct the rubberband baseline across the spectrum. This parameter effectively determines how finely the spectral range is segmented when calculating the convex envelope. According to the OPUS documentation, recommended values typically range from 10 to 200, with a default value of 64. Increasing this value increases the density of baseline points and can provide a more detailed baseline representation for spectra with complex background

variations.

However, the effective influence of this parameter also depends on both the number of data points in the spectrum and the spectral shape. First, setting a "Number of baseline points" value larger than 200 or smaller than 10 produces essentially the same baseline as using the boundary values of 200 or 10, respectively. Second, when the raw spectrum is relatively smooth, as is the case for the ν_{OD} band in our data, though not for the $\nu_{free\text{-}OD}$ band, the choice of "Number of baseline points" has only a minor influence on the resulting baseline. Importantly, the positions of these baseline points are determined automatically by the algorithm based on the spectral shape, rather than being manually selected by the user.

An advantage of this approach is that the baseline is determined from the global geometric structure of the spectrum, rather than from local fitting of individual peak shapes. Consequently, slowly varying background contributions (e.g., broad absorption bands or instrumental drift) are captured by the baseline envelope, while sharper vibrational features remain largely unaffected. This reduces the risk of distorting narrow spectral peaks compared with polynomial or aggressive automated fitting procedures.

To further validate the robustness of the method, we performed additional tests by varying the number of baseline points used in the convex-hull construction. These tests show that the resulting baseline changes only slightly (see **Supplementary Fig. T2d-e**). This confirms that the conclusions drawn from the spectral analysis are not sensitive to the specific parameters used in the baseline correction.

Further details of the rubberband baseline method can be found in Refs. 8 and 9.

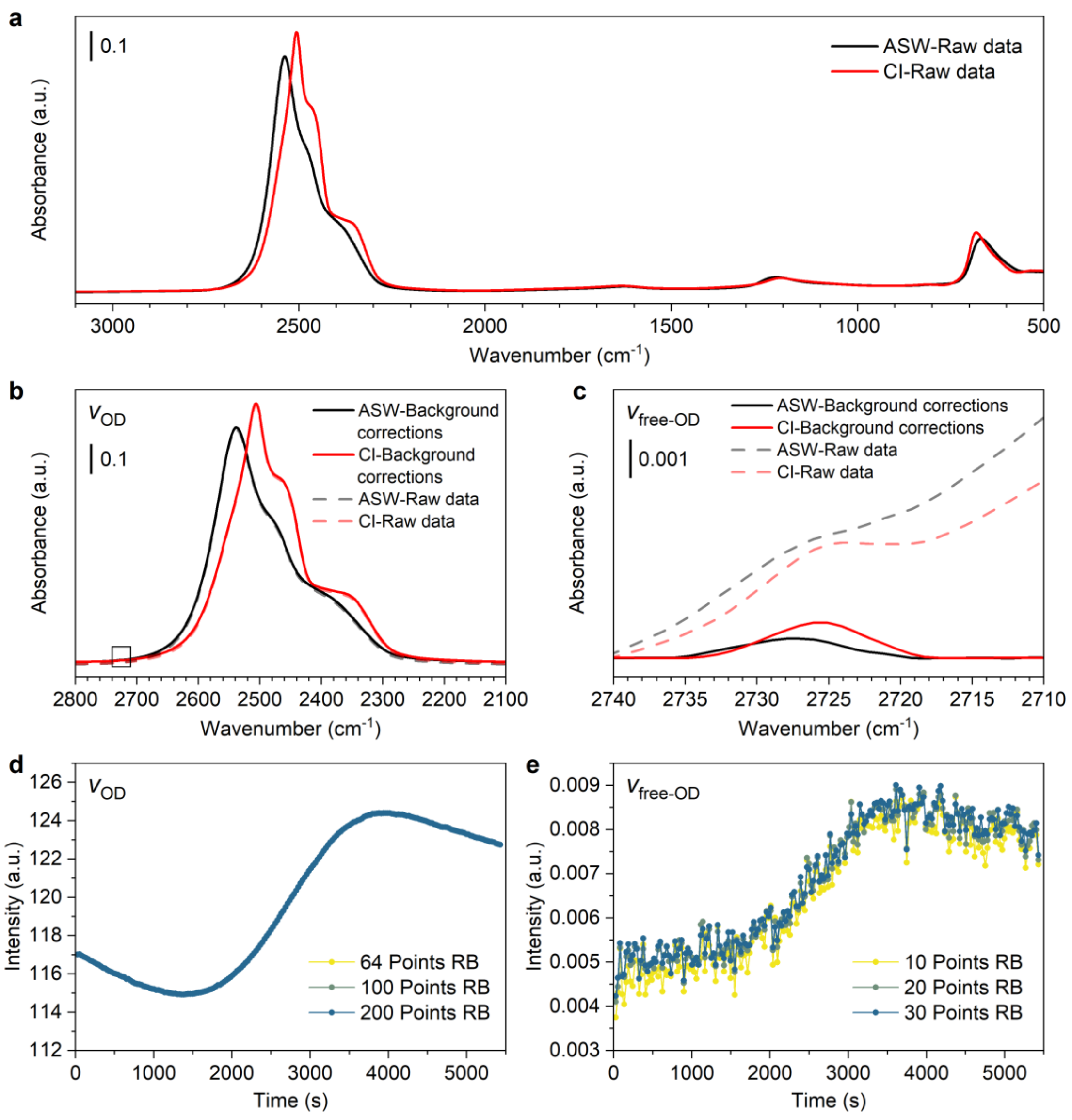

Supplementary Fig. T2. (**a**) Full IRRAS spectra of thick ASW and CI films, showing the stretching, bending and librational vibrational regions. (**b, c**) IRRAS spectra of the ν_{OD} and $\nu_{free\text{-}OD}$ regions for thick ASW and CI films before and after background corrections using the rubberband algorithm with 64 and 10 baseline points, respectively. (**d, e**) Comparison of the integrated peak areas of the ν_{OD} and $\nu_{free\text{-}OD}$ bands obtained using the rubberband algorithm with different numbers of baseline points. The spectra used in (**d**) and (**e**) are identical to those shown in **Fig. 2a** and **Fig. 3a** of the main manuscript.

However, from the plot in Figure T2(c), it does look like the method works well by eye, but this can be misleading. How does the method distinguish the smooth underlying absorption band of the hydrogen-bonded OH from the free-OH? What does the number of dots mean? How are these dots chosen and how does this affect the data?

Author Reply:

We thank the reviewer for these important questions.

The ν_{OD} band forms a broad and smoothly varying absorption background that extends over several hundred cm^{-1}, whereas the $\nu_{free\text{-}OD}$ band appears as a narrow and well-defined peak at the high-frequency edge of this band. The rubberband (convex-hull) algorithm determines the baseline as the lower convex envelope of the spectrum. Because the baseline points correspond to local minima in the spectrum, the resulting baseline typically follows the smooth wings of the ν_{OD} band rather than the maximum of the $\nu_{free\text{-}OD}$ peak. Consequently, the baseline passes below the free OD feature, allowing its intensity to be preserved after subtraction (**Supplementary Fig. T2c**).

The meaning of the "dots" (i.e., the baseline points used in the rubberband algorithm) and their influence on the rubberband baseline are described above in the explanation of the rubberband algorithm. In brief, these points define the number and distribution of sampling points used to construct the convex envelope across the spectral range. Increasing the number of baseline points allows the baseline to more closely follow slow variations in the spectral background, whereas too few points may lead to an overly coarse baseline representation. In our analysis, the chosen parameter range has a negligible effect on the results of the data analysis (**Supplementary Fig. T2d-e**).

Would you get a similar result with a simple two-point linear baseline subtraction? This would be more convincing. Is this the same as 2 dots in the rubberband method?

I am in general very skeptical about automated and/or complex baseline subtractions as these can significantly perturb the data.

Author Reply:

We thank the reviewer for this helpful suggestion and for raising this important concern. To address this point, we performed an additional test using a simple two-point linear baseline subtraction using spectral regions on either side of the peak (see **Supplementary Fig. T3**). For the broad ν_{OD} absorption band, this simple approach yields peak intensities very similar to those obtained with the rubberband method.

However, in the $\nu_{free\text{-}OD}$ region the underlying background exhibits noticeable curvature. In this case, a two-point linear baseline cannot adequately capture the shape of the background and may introduce distortions in the extracted peak intensity. By contrast, the rubberband method estimates the baseline as the lower convex envelope of the spectrum, with baseline points ("dots") determined automatically from local minima using a convex-hull algorithm. This allows the baseline to follow the slowly varying background more accurately.

Importantly, these automatically determined baseline points are not equivalent to manually selecting two points for a linear baseline. Instead, they correspond to multiple local minima across the spectrum that collectively constrain the baseline shape, as described above in the explanation of the rubberband algorithm, which has now been added to **Supplementary Text. 2**. Furthermore, in the OPUS implementation, setting the "Number of baseline points" parameter to values smaller than 10 (e.g., 2), produces results equivalent to those obtained with 10 baseline points (**Supplementary Fig. T3d-e**).

Revision in the Supplementary Information:

Page 3, line 78: A new figure has been added to illustrate the difference between the rubberband baseline method and the two-point linear baseline subtraction.

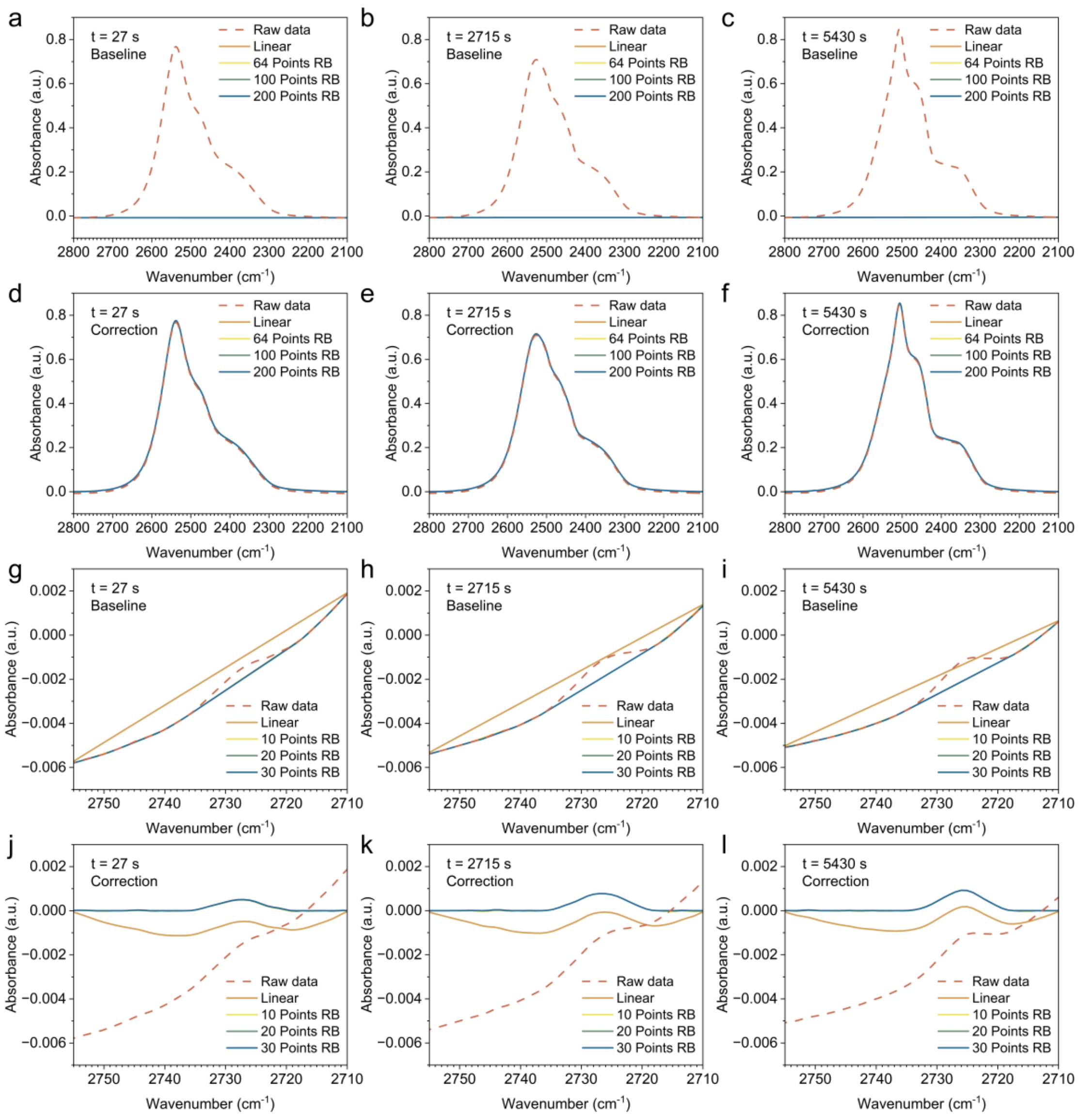

Supplementary Fig. T3. Raw and baseline-corrected IRRAS spectra of ice films at three representative phase-transition time points (before, during, and after crystallization). (**a – c**) Raw spectra in the ν_{OD} region with the different baseline treatments. (**d – f**) Baseline-corrected spectra in the ν_{OD} region. (**g – i**) Raw spectra in the $\nu_{free\text{-}OD}$ region with the different baseline treatments. (**j – l**) Baseline-corrected spectra in the $\nu_{free\text{-}OD}$ region.

Reviewer 3 also had comments on the "lack of coherence among the results" and "incoherence of the conclusions". The authors ascribe this to specific observations, but I would think they were mean general in nature. When I read the short discussion starting line 312, I find the second paragraph starting line 326 rather clear but I cannot follow the first paragraph. The authors need reword this discussion to make it clearer.

Author Reply:

We thank the reviewer for this helpful suggestion. Accordingly, we have revised and reworded this section to enhance the clarity and logical flow of the discussion. The updated text is shown below.

Revision in the manuscript:

Page 9, line 313-325:

Surface stabilization is thermodynamically driven by the minimization of surface free energy through molecular reorganization. In the absence of bulk crystallization (e.g., in pure ASW or fully CI), the process is governed by the free energy difference (ΔG) between the initial and final surface configurations at a given temperature. Consequently, the stabilization rate r_{stbl} depends on both ΔG and temperature. During the ASW-to-CI transition, however, surface restructuring occurs concurrently with bulk crystallization. The surface dynamics are therefore governed by two coupled processes: the crystallization rate k and the stabilization rate r_{stbl}. For isothermal crystallization at 155 K following a rapid temperature jump from 150 K, the initial value of ΔG is estimated to be 973 J/mol from the measured desorption rates of ASW and CI films (see **Supplementary Text** and **Supplementary Figs. 20** and **21**) [36]. As crystallization proceeds and the crystalline fraction increases, ΔG progressively decreases, reducing the thermodynamic driving force for further surface restructuring. Under UHV conditions, thermal desorption is unavoidable and becomes increasingly significant at elevated temperatures, potentially leading to a regime in which desorption dominates the surface dynamics. Consequently, the transient surface structure during the ASW-to-CI transformation is governed by the interplay among crystallization, surface stabilization, and thermal desorption, a regime requiring further exploration.
